# Fungi associated with Norway spruce and its bark beetles detoxify spruce defense compounds by conjugation to α-ribose

**DOI:** 10.1093/ismejo/wrag230

**Published:** 2026-09-04

**Authors:** Shivani Kiran Nimbkar, Yoko Nakamura, Baoyu Hu, Benke Hong, Beate Rothe, Michael Reichelt, Maximilian Lehenberger, Guilin Hu, Julián D Restrepo-Leal, Xingcong Jiang, Jonathan Gershenzon, Ruo Sun

**Affiliations:** Department of Biochemistry, Max Planck Institute for Chemical Ecology, Jena 07745, Germany; Department of Natural Product Biosynthesis, Max Planck Institute for Chemical Ecology, Jena 07745, Germany; Nuclear Magnetic Resonance (NMR)/Biosynthesis Group, Max Planck Institute for Chemical Ecology, Jena 07745, Germany; Department of Biochemistry, Max Planck Institute for Chemical Ecology, Jena 07745, Germany; Department of Natural Product Biosynthesis, Max Planck Institute for Chemical Ecology, Jena 07745, Germany; Zhejiang Key Laboratory of Precise Synthesis of Functional Molecules, Department of Chemistry, School of Science and Research Center for Industries of the Future, Westlake University, Hangzhou 310030, China; Department of Biochemistry, Max Planck Institute for Chemical Ecology, Jena 07745, Germany; Department of Biochemistry, Max Planck Institute for Chemical Ecology, Jena 07745, Germany; Department of Biochemistry, Max Planck Institute for Chemical Ecology, Jena 07745, Germany; Department of Biochemistry, Max Planck Institute for Chemical Ecology, Jena 07745, Germany; Kunming Institute of Botany, Chinese Academy of Sciences, Kunming 650201, China; Department of Biochemistry, Max Planck Institute for Chemical Ecology, Jena 07745, Germany; Centre for Excellence in Molecular Plant Sciences, Institute of Plant Physiology and Ecology, Chinese Academy of Sciences, Shanghai 200000, China; Department of Biochemistry, Max Planck Institute for Chemical Ecology, Jena 07745, Germany; Faculty of Forestry and Wood Sciences, Czech University of Life Sciences Prague, Praha-Suchdol 16521, Czech Republic; Department of Biochemistry, Max Planck Institute for Chemical Ecology, Jena 07745, Germany

**Keywords:** plant chemical defense, detoxification, tree associated fungi, *Ips typographus* symbionts, phenolic compounds, Norway spruce tree

## Abstract

Fungi readily colonize the inner bark (phloem) of spruce and other conifers despite these tissues having a high concentration of antifungal defense metabolites. These compounds include stilbenes, flavonoids, and other phenolic substances, mostly present as glucosides. Yet, the underlying biochemical mechanisms by which fungi resist conifer phenolics remain largely unresolved. Using untargeted metabolomics, structural elucidation and biological and biochemical assays, we investigated how and why fungi metabolize the major phenolics of Norway spruce (*Picea abies*). Various fungi, including those associated with bark beetles, were found to hydrolyze stilbene glucosides to their corresponding aglucones. Two basidiomycetes, the saprotroph *Coprinellus radians* and *Cylindrobasidium ipidophilum*, a symbiont of the Eurasian spruce bark beetle *Ips typographus*, then converted the stilbene aglucones into α-ribofuranosylated derivatives, revealing a previously unrecognized pathway in tree-colonizing fungi. Ribosylation markedly reduced the antifungal activity of the aglucones and stabilized them against hydrolysis by fungal and *I. typographus* enzymes, preventing regeneration of the toxic aglucones. Ribosylation was also correlated with increased growth on spruce bark-containing medium. Hence, the ability to overcome major conifer bark defenses by conversion of toxic stilbene aglucones to non-toxic α-ribosides may explain the successful colonization of this tissue by fungi, some of which support *I. typographus* attack.

## Graphical Abstract

**Graphical Abstract:**
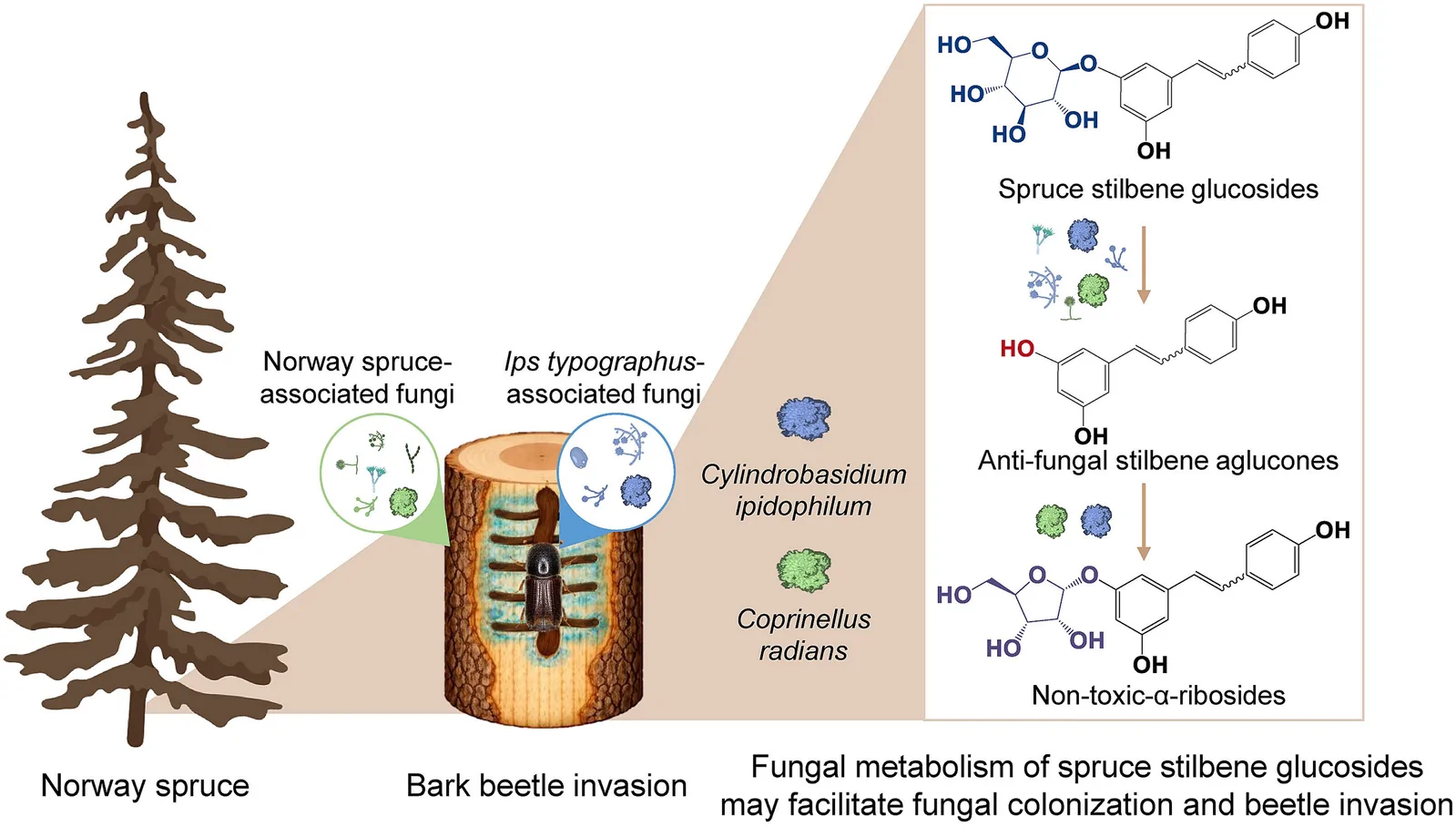

The tree and fungi in the graphic abstract were created in BioRender. Sun, R. (2026) https://BioRender.com/ybfn6h1, and the bark beetle and beetle galleries image in the graphic abstract was generated using ChatGPT (OpenAI).

## Introduction

Bark beetle outbreaks have increased dramatically in recent years due to rising global temperatures [1]. Although the majority of these species colonize trees that are weak or recently killed, a few attack healthy trees and have caused extensive damage in forests throughout the world, especially conifer forests. Conifer bark beetles are invariably associated with free-living fungal symbionts that are introduced into the tree upon beetle invasion [2–5]. After proliferation, these fungi are thought to provide beetles with nutrients, detoxify tree defense compounds and help to kill the tree [6, 7]. Despite the ecological importance of these fungal symbionts, however, it is still not clear how they persist in tree tissue, which may contain high concentrations of anti-fungal defenses [7–9]. Such compounds may shape the size and composition of the fungal community, especially during bark beetle attack, when defense accumulation is enhanced.

Conifer bark beetles are typically associated with fungi known as ophiostomatoids (orders *Ophiostomatales* and *Microascales*), which are sometimes referred to as blue-stain fungi. For example, the well-studied Eurasian spruce bark beetle *Ips typographus* is known to associate with a core microbiome [10], containing ophiostomatoids such as *Grosmannia penicillata* and *Endoconidiophora polonica* [11, 12]. In addition, there are potential core microbiome members, such as the agaricalean basidiomycete *Cylindrobasidium ipidophilum*, which is a saprotrophic fungus [13] that frequently sporulates in *I. typographus* galleries [13–15], but their roles in beetle–fungus–tree interactions have not been explored. Besides bark beetle symbionts, there are many other fungi living in conifer trees, including plant pathogens, saprotrophs, and asymptomatic endosymbionts [16, 17]. It is important to understand how these fungi tolerate tree antifungal defenses as well because they may compete with bark beetle symbionts and affect the success of beetle attack. However, little is known about this group aside from information about a few well-known pathogens [18].

Bark beetles attacking conifers feed and raise their brood on the phloem (inner bark). In this tissue, conifers accumulate a diverse suite of phenolic defense compounds that strongly inhibit fungal growth [19–22]. These defenses are dominated by stilbenes and flavonoids that accumulate at high concentrations in specialized parenchyma cells of the phloem [23, 24]. Fungi could resist phenolics by avoidance of high concentrations, rapid excretion, target site insensitivity or metabolic transformation. Among bark beetle-associated fungi, most studies have focused on the metabolic transformations of a single species, the *I. typographus* associate *E. polonica*, which has been shown to metabolize phenolics by hydrolysis, oxidative cleavage and dimerization [25, 26]. However, the extent to which other fungal symbionts of bark beetles metabolize phenolic compounds remains largely unknown.

Fungi employ a variety of metabolic transformations to overcome plant chemical defenses [27]. Among these, glycosyltransferases catalyze the conjugation of sugars to lipophilic defenses thereby reducing their toxicity, enhancing water solubility and promoting excretion [28–33]. For instance, the rice sheath blight pathogen *Rhizoctonia solani* detoxifies the rice flavonoid sakuranetin via xylosylation [34], and the entomopathogenic fungus *Beauveria bassiana* converts phenolic aglucones into non-toxic methyl-glycosylated derivatives [35]. These examples illustrate how sugar conjugation represents a versatile fungal strategy for neutralizing plant chemical defenses; yet, the occurrence of glycosylation-based pathways in forest fungi remains underexplored.

Here, we investigate the metabolism of phenolic defenses by a variety of fungi colonizing the European conifer Norway spruce (*Picea abies*), a dominant forest tree of northern and central Europe. Some are symbionts of *I. typographus*–associated taxa, while others are not associated directly with bark beetles, but are pathogens, saprotrophs and endophytes isolated from Norway spruce without bark beetle invasion (referred to as Norway spruce–associated fungi). These include the pathogen *Heterobasidion annosum* (Russulales), the saprotroph *Coprinellus radians* (Agaricales), and the endophyte *Biscogniauxia nummularia* (Xylariales). Although these are not directly associated with *I. typographus*, they may interact with the symbiotic fungi of the beetle, and so influence the outcome of invasion. Using a combination of untargeted and targeted metabolomics analyses, we examined the fate of phenolic compounds during fungal colonization of spruce tissues. We show that these fungi not only hydrolyze phenolic glucosides, but two basidiomycetes convert the resulting aglucones into non-toxic α-ribosylated derivatives that resist enzymatic hydrolysis. Our results extend fungal phenolic metabolism beyond deglycosylation and oxidative degradation, and highlight the metabolic versatility that may underpin fungal success in environments with abundant anti-fungal defense compounds.

## Materials and methods

### Fungal cultures

Fungal isolates are listed in the Supplementary Methods. Genomic DNA of fungi was extracted using the DNeasy Blood & Tissue Kit (Qiagen, Hilden, Germany). Fungal strains were identified by sequencing the internal transcribed spacer (ITS) regions, following cloning and polymerase chain reaction amplification as previously described [36]. The primer pairs used for ITS amplification are listed in Table S1. The ITS rRNA gene sequences of the fungi provided in Supplementary File 1. Taxonomic assignment was performed using the UNITE 10.0 database. The ITS rRNA gene sequence of the *G. penicillata* isolate CMW43738 was obtained from the NCBI database. Phylogenetic analysis of the ITS rRNA gene regions from these isolates was conducted by aligning nucleic acid sequences using MUSCLE. An Unweighted Pair Group Method with Arithmetic Mean (UPGMA) tree was generated in Geneious Prime, with branch labels representing consensus support (%).

### Norway spruce phloem samples after *Ips typographus* and fungal infection

Norway spruce phloem samples, including uninfected tissue, fungal-infected tissue, the phloem at the margin of *I. typographus* galleries, and phloem at the main beetle galleries, were collected from spruce logs infested by beetles obtained from our laboratory colony. The detailed collection method and extraction method is listed in the Supplementary Methods. Extracts were analyzed for phenolic compounds using liquid chromatography–tandem mass spectrometry (LC–MS/MS).

The Norway spruce phloem samples used to compare the phenolic content of healthy trees and bark beetle-infested trees were collected from the Kranichfeld forest (Thuringia, Germany) with details given in the Supplementary Methods.

### Astringin synthesis and nuclear magnetic resonance characterization

Astringin was synthesized according to a previously described method [37] and the spectroscopic data were in accordance with the reported values. Nuclear magnetic resonance (NMR) spectra were measured on a 400 MHz Bruker Avance III HD spectrometer (Bruker Biospin GmbH, Rheinstetten, Germany). For spectrometer control and data processing Bruker TopSpin ver. 3.6.1 was used. The detailed method is listed in the Supplementary Methods.

### Targeted metabolite analyses using liquid chromatography–tandem mass spectrometry

Targeted analyses of phenolic compounds were conducted using an Agilent HP1260 series HPLC instrument (Agilent Technologies, Böblingen, Germany) coupled to a Triple Quad 6500+ system (SCIEX, MA, USA). Compounds were separated using an Agilent Zorbax Eclipse XDB-C18 column (50 × 4.6 mm × 1.8 μm, Agilent Technologies, Wilmington, DE, USA) with mobile phase A (0.05% formic acid in Milli-Q water) and mobile phase B (acetonitrile). The elution profile is described in the Supplementary Methods. Detection of each compound was achieved through multiple reaction monitoring (MRM) of specific parent-to-product ion transitions for each compound. Detailed parameters are described in Table S2. Analyst 1.6.3 (SCIEX) and MultiQuant 3.0.3 software (SCIEX) were used for data acquisition and processing, respectively. Quantification of individual compounds was based on external standard calibration curves, with the sources of external standards listed in Table S3.

### Samples from fungi on spruce bark-containing agar

To analyze metabolic changes in spruce bark-containing medium after fungal incubation, we used potato dextrose agar (PDA) containing 1%–5% spruce bark (SB-PDA, detailed in the Supplementary Methods), with fungi incubated at 25°C for 4 to 12 days. These cultures were compared to those grown on PDA medium without spruce bark and to those grown on SB-PDA medium without fungus. The harvested mycelia were freeze-dried, weighed, and extracted using 80% methanol. The harvested medium was weighed and extracted using 80% methanol. Five replicates were performed for each treatment. Extracts were subjected to untargeted metabolomic analysis using an ultra-high performance liquid chromatography system coupled to a quadrupole time-of-flight mass spectrometer (UHPLC–qTOF–MS).

### Untargeted metabolomic analyses using ultra-high performance liquid chromatography system coupled to a quadrupole time-of-flight mass spectrometer

Metabolite extracts were analyzed using an UHPLC system (Thermo Dionex Ultimate 3000, MA, USA) coupled to a qTOF-MS system (Bruker timsTOF, Bremen, Germany). The UHPLC was equipped with a Zorbax Eclipse XDB-C18 reversed-phase column (1.8 μm, 2.1 × 100 mm; Agilent Technologies, Böblingen, Germany). The LC and mass spectrometry settings are detailed in the Supplementary Methods. Data processing was performed using MetaboScape 5.3 software (Bruker, Bremen, Germany) and MetaboAnalyst 6.0 (https://dev.metaboanalyst.ca/).

### Tracking fungal biotransformation using ^13^C_6_-labeled resveratrol

To trace the metabolic fate of resveratrol in *C. radians* and *C. ipidophilum*, fungi were inoculated on PDA medium supplemented with 100 μM resveratrol or 100 μM [^13^C_6_] resveratrol (Sigma, Taufkirchen, Germany), labeled in the monohydroxylated ring. Sample extractions are detailed in Supplementary Methods. Subsequently, the extracted compounds were analyzed using untargeted UHPLC–qTOF–MS.

### Compound isolation and nuclear magnetic resonance analyses

The structures of stilbene metabolites produced by fungi were confirmed through isolation, chromatographical purification, and NMR analysis. Resveratrol-3-*O*-α-ribofuranoside, resveratrol-3,5-*O*-α-biribofuranoside, isorhapontigenin-3-*O*-α-ribofuranoside, and piceatannol-11-*O*-α-ribofuranoside were isolated from fungal cultures and confirmed by NMR measurements as described in the Supplementary Methods.

### Metabolism of phenolic compounds by fungi

*C. radians* and *C. ipidophilum* were inoculated on PDA supplemented with glucosides astringin, piceid or isorhapontin. Plates containing 0.1% DMSO served as negative controls. Sample extractions are detailed in the Supplementary Methods. Metabolites from both the fungal biomass and medium were extracted and analyzed using UHPLC–qTOF–MS and LC–MS/MS.

To further test the metabolism of the stilbene compounds by Norway spruce- and *I. typographus*–associated fungi, as well as additional basidiomycete species, fungi were incubated with 200 μM of piceid. This experiment is described in the Supplementary Methods in more detail.

### Growth of fungi on stilbene metabolites

The effect of stilbene compounds on the growth of *C. radians*, *C. ipidophilum*, *and H. annosum* was assessed using glucosides (piceid and isorhapontin), aglucones (resveratrol and isorhapontigenin), and ribosides (resveratrol-3-*O*-α-ribofuranoside and isorhapontigenin-3-*O*-α-ribofuranoside). Compounds were applied at concentrations of 0, 50, 100, 200, 400, and 800 μM in PDA medium. Four replicates were performed for each treatment. Fungi were incubated on the plates (35 × 10 mm) at 25°C for 4 days for *C. radians* and *H. annosum*, and 6 days for *C. ipidophilum*. Images were taken for area analysis using ImageJ software.

IC_50_ values were determined by nonlinear curve fitting using a biphasic dose–response function. The concentrations of compounds were log₁₀-transformed, and percent inhibition was calculated based on fungal growth area at different concentrations relative to the control group, using the following formula:


\begin{align*} \mathrm{Inhibition} \left(\%\right)\!=&\,100\times \left(\mathrm{Area}\ \mathrm{of}\ \mathrm{control}\ \mathrm{group}\hbox{--} \mathrm{Area}\ \mathrm{of}\ \mathrm{treatment}\right)/\\ &\mathrm{Area}\ \mathrm{of}\ \mathrm{control}\ \mathrm{group} \end{align*}

### *In vitro* enzyme assay of crude extracted proteins

Proteins were extracted from *C. radians* and *C. ipidophilum* cultures, as well as *I. typographus* pupa as described in the Supplementary Methods. The deglycosylation enzyme assays were modified from a previously published method [38], and consisted of 20 μg of protein from each sample incubated with 500 μM of stilbene glucosides (piceid or isorhapontin) or stilbene ribosides (resveratrol-3-*O*-α-ribofuranoside or isorhapontigenin-3-*O*-α-ribofuranoside) in 200 μL phosphate buffer (50 mM, pH 6.4) at 30°C for 2 h. Negative controls were prepared by boiling protein samples at 95°C for 10 min to inactivate enzyme activity. Reactions were terminated by transferring 10 μL of the mixture into 190 μL of pure methanol and freezing at −20°C. The samples were analyzed by LC–MS/MS.

### Growth of fungi on spruce bark-containing medium and their biotransformation

The effects of spruce bark on the growth of *C. radians*, *C. ipidophilum*, and *Pleurotus ostreatus* were assessed using SB-PDA containing 0%, 0.5%, 1%, or 2% (w/v) spruce bark. Five biological replicates were performed for each treatment. Fungi were incubated on 60 × 15 mm plates at 25°C for 6 days (*C. radians* and *P. ostreatus*) or 8 days (*C. ipidophilum*). Colony images were acquired and analyzed using ImageJ software to determine fungal growth.

The formation of piceatannol-11-*O*-α-ribofuranoside, isorhapontigenin-3-*O*-α-ribofuranoside, and resveratrol-3-*O*-α-ribofuranoside was analyzed by LC–MS/MS as described above. Fungal mycelia were collected, freeze-dried, weighed, and extracted prior to metabolite analysis.

### Statistical analyses

Details are in the Supplementary Methods.

## Results

### Degradation of phenolics in Norway spruce phloem during fungal and *Ips typographus* colonization

The phloem (inner bark) of Norway spruce (*P. abies*) contains high concentrations of antifungal phenolics, predominantly stored as glucosides [38]. Despite these chemical defenses, certain species of fungi are known to readily colonize the phloem of Norway spruce and spread extensively through phloem tissues. These include tree pathogens, saprophytes and endophytes (Fig. 1a) as well as *I. typographus*–vectored ectosymbiotic species that proliferate in galleries excavated by beetles during colonization (Fig. 1b).

**Figure 1:**
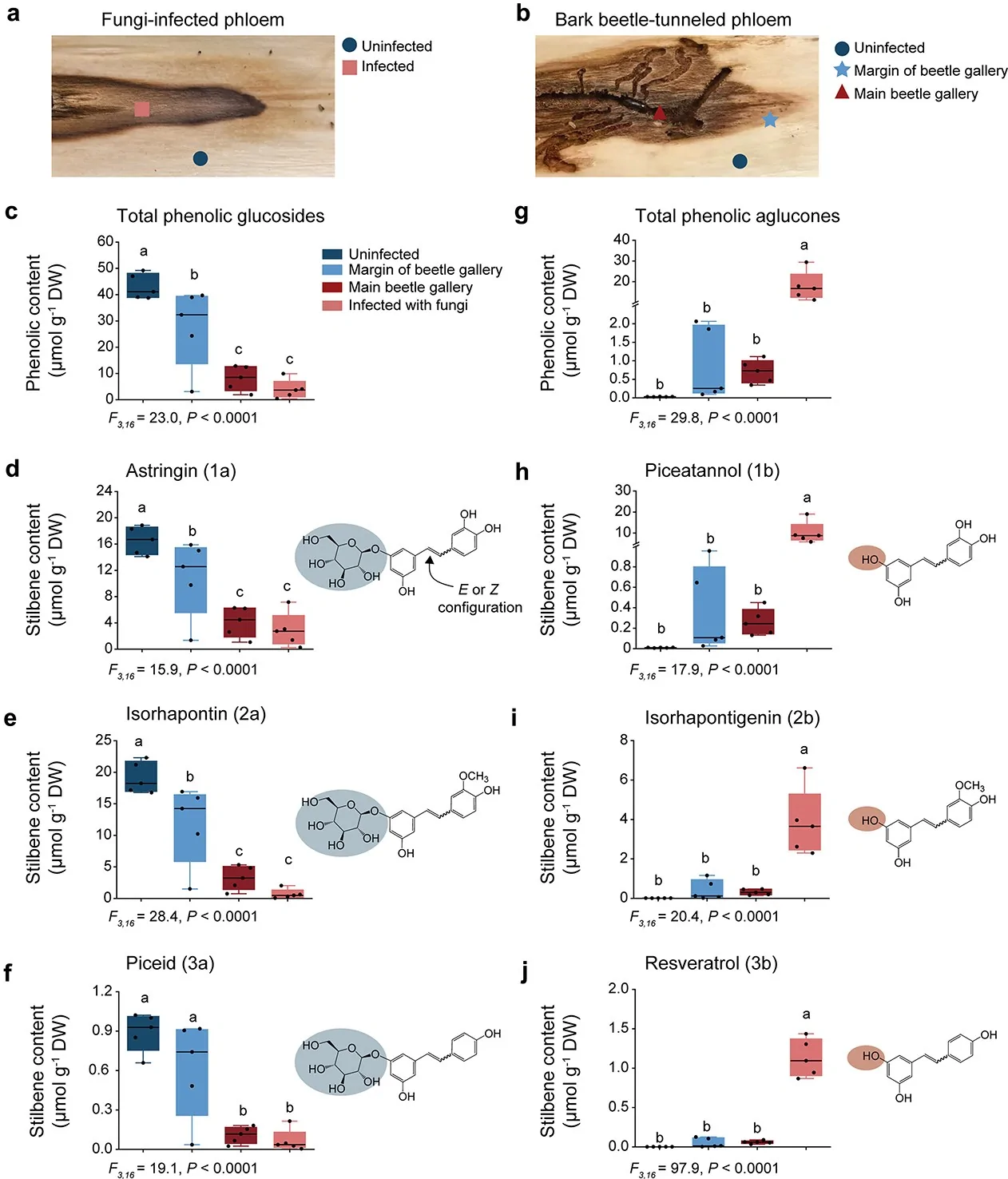
Phenolic glucosides in the phloem of Norway spruce are degraded following fungal colonization and infestation by bark beetles and their associated fungi. (a and b) Representative images of Norway spruce phloem after fungal infection (a) and bark beetle (*Ips typographus*) infestation with associated fungi (b). Photographs were taken of spruce logs collected in the field for our laboratory bark beetle culture that were either protected from attack by bark beetles (a, fungal growth from species already present in the log) or attacked by bark beetles (b, fungal growth from beetle-colonized fungi plus those already in the log). Sampling locations for phenolic analyses include uninfected phloem, infected phloem, margin of beetle gallery, and main beetle gallery. (c–f) Quantification of total amount of phenolic glucosides (c), as well as the phenolic glucosides astringin (d), isorhapontin (e), and piceid (f) in the phloem samples (*n* = 5). The total amount of phenolic glucosides includes the amounts of astringin, isorhapontin, piceid, taxifolin-3′-*O*-glucoside, and quercetin-3′-*O*-glucoside (Fig. S1a). (g–j) Quantification of total amount of phenolic aglucones (g), as well as piceatannol (h), isorhapontigenin (i), and resveratrol (j) in the phloem samples (*n* = 5). The total amount of phenolic aglucones includes the amounts of piceatannol, isorhapontigenin, resveratrol, taxifolin, and quercetin (Fig. S1b). The amounts of *E-* and *Z-* isomers of each stilbene were combined, e.g. compound **1a** represents the combined quantity of ***E*-1a** and ***Z*-1a**. The *Z*-isomer was usually present at 5%–10% of the *E*-isomer [38]. In the chemical structures, glucose and aglucone moieties are highlighted with light blue and red ovals, respectively. Box plots represent the range of 25 to 75%, with center lines indicating median values and whiskers extending to 1 time the interquartile range. Statistical significance among groups was determined by one-way ANOVA followed by Fisher’s LSD tests (*P* < .05) in c-j. Different lowercase letters indicate statistically significant differences.

To learn whether fungal proliferation can alter these phloem defenses, we analyzed the major phenolic compounds using LC–MS/MS. The total concentration of phenolic glucosides, including stilbenes and flavonoids, in the phloem was significantly reduced in the main galleries of *I. typographus* and their margins compared with uninfected controls (due presumably to the combined effects of the bark beetles and their associated fungi) (Fig. 1c). These compounds were also markedly reduced in phloem tissue that was infected by Norway spruce–associated fungi alone (without any fungi introduced by *I. typographus* invasion). Specifically, the stilbene glucosides astringin (**1a**), isorhapontin (**2a**), and piceid (**3a**) were markedly depleted in *I. typographus* galleries and in phloem colonized by Norway spruce–associated fungi alone (Fig. 1d–f). Similarly, the major flavonoid glucosides, taxifolin-3′-*O*-glucoside (**4a**) and quercetin-3′-*O*-glucoside (**5a**), were consistently reduced phloem infected by Norway spruce–associated fungi alone and after attack by *I. typographus* and their associated fungi (Fig. S1a). In contrast, the corresponding aglucones accumulated in phloem colonized by Norway spruce–associated fungi alone. Total phenolic aglucone levels, including piceatannol (**1b**), isorhapontigenin (**2b**), resveratrol (**3b**), taxifolin (**4b**), and quercetin (**5b**), were significantly higher in tissues infected with Norway spruce–associated fungus compared with uninfected phloem (Fig. 1g–j and Fig. S1b). Aglucones were also readily detectable in phloem from main beetle galleries and gallery margins, but their concentrations did not differ significantly from those in uninfected controls (Fig. 1g–j).

The increase of aglucones was substantially lower than the decline in glucoside concentrations in Norway spruce–associated fungi and *I. typographus*-infested tissues (Fig. 1c and g), suggesting that the aglucones are subject to further metabolism. For example, total phenolic glucosides decreased by ~35 μmol g^−1^ in the main beetle galleries and 39 μmol g^−1^ in phloem infected by Norway spruce–associated fungi alone from the amounts in the uninfected controls, whereas aglucone accumulation in these sites reached only 0.9 μmol g^−1^ and ~ 18 μmol g^−1^, respectively (Fig. 1c and g). The large decline in phenolic glucosides observed after Norway spruce–associated fungal and *I. typographus* infestation was not seen after spruce phloem was mechanically damaged (Fig. S1c), and thus can be considered a direct result of fungal and beetle metabolism. In phloem samples of mature Norway spruce trees, the content of stilbene glucosides in the bark, particularly astringin and isorhapontin, was even higher in the bark of spruce trees during the early stages of bark beetle infestation compared to healthy trees (Fig. S1d). After two months of bark beetle infestation, however, both stilbene glucosides and aglucones were significantly degraded in the bark of beetle-attacked trees (Fig. S1d). This suggests that the combined attacks of bark beetles and their associated microorganisms metabolize this class of defense chemicals. Together, these results demonstrate that fungal communities associated with Norway spruce and its specialist bark beetles may possess substantial capacity to metabolize host tree phenolics, extending beyond simple deglycosylation and suggesting further downstream processing.

### Metabolism of stilbene glucosides by individual fungi

We investigated the metabolism of the stilbene glucosides, the main group of phenolic glucosides in Norway spruce phloem, by individual fungi, including three representative bark beetle–associated fungi vectored into spruce phloem during host tree colonization, *Cylindrobasidium ipidophilum*, *Endoconidiophora polonica*, and *Grosmannia penicillata* (Fig. 2). We also chose three Norway spruce–associated fungi isolated from the phloem of trees prior to bark beetle invasion, including the saprotroph *C. radians*, the spruce pathogen *H. annosum* and the endophyte *B. nummularia* (Fig. 2). Untargeted metabolomic analyses were performed to compare fungal metabolites after incubation on spruce bark–supplemented potato dextrose agar (SB-PDA) with non-inoculated SB-PDA controls. In most fungal strains, incubation resulted in a significant reduction of the phenolic glucosides astringin (**1a**), isorhapontin (**2a**), and piceid (**3a**) relative to uninoculated controls (Fig. 2c and d). This decrease was generally accompanied by a significant accumulation of the corresponding aglucones piceatannol (**1b**), isorhapontigenin (**2b**), and resveratrol (**3b**), as well as piceatannol dimers, particularly in cultures of *B. nummularia*, *C. ipidophilum*, *E. polonica*, and *G. penicillata* (Fig. 2c and d).

**Figure 2:**
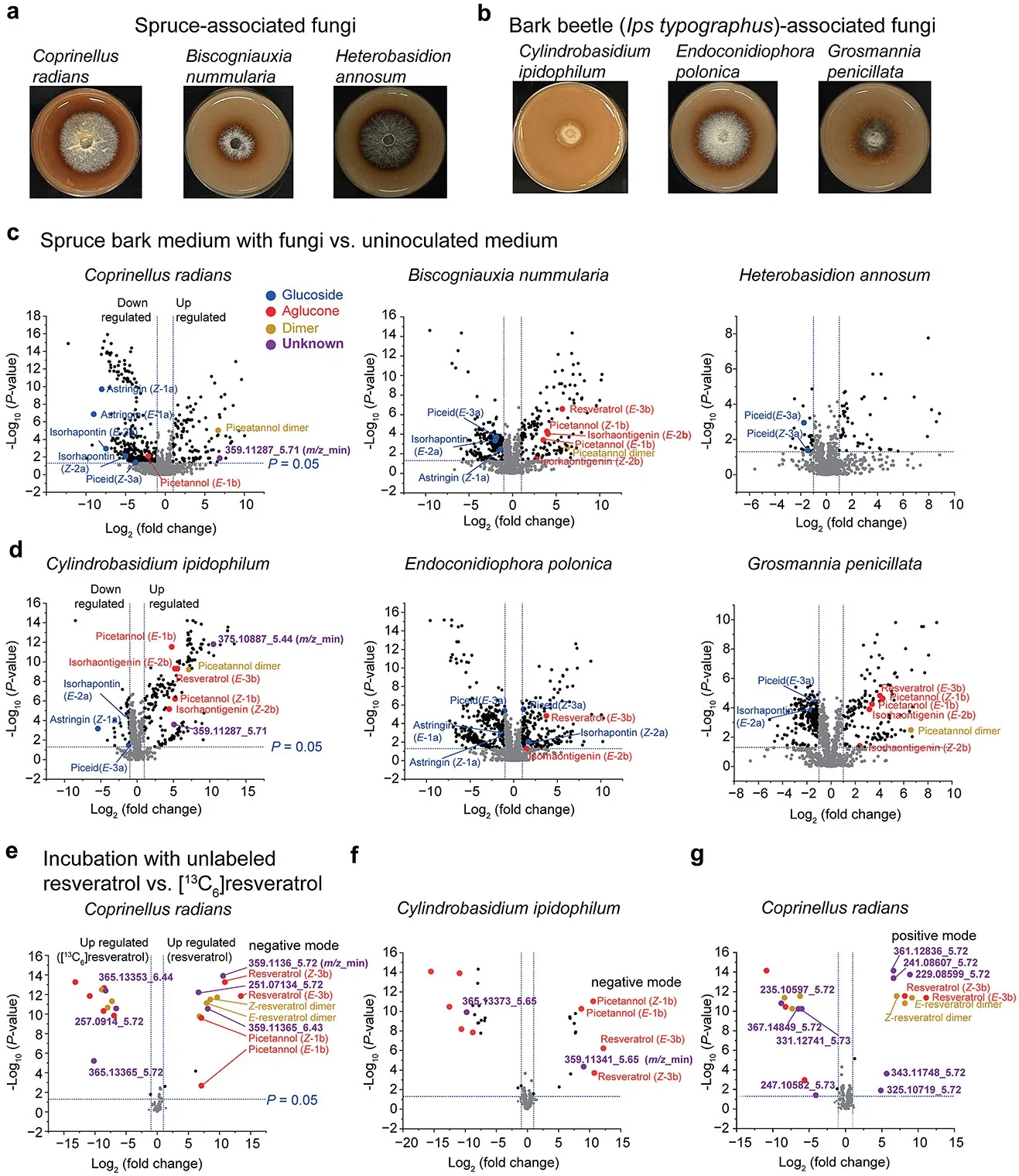
Spruce stilbene glucosides are metabolized by spruce tree—and bark beetle—associated fungi. (a and b) Representative images of spruce tree–associated fungi (*C. radians*, *B. nummularia*, and *H. annosum*) (a) and bark beetle (*I. Typographus*)–associated fungi (*Cylindrobasidium ipidophilum*, *Endoconidiophora polonica*, and *Grosmannia penicillata*) (b) grown on SB-PDA. (c and d) Volcano plots illustrate features (*m/z* value, retention time in min) derived from untargeted UHPLC–qTOF–MS analyses of spruce tree–associated fungi (c) and *I. typographus*–associated fungi (d) incubated on SB-PDA medium compared with uninoculated medium in the negative mode (*n* = 5). (e–g) Volcano plots illustrate features (*m/z* value, retention time in min) derived from untargeted UHPLC–qTOF–MS analyses of fungi *C. radians* (e) and *C. ipidophilum* (f) on PDA supplemented with unlabeled resveratrol versus [^13^C_6_] resveratrol (labeled in the monohydroxylated ring) in the negative mode (*n* = 4–5), as well as *C. radians* (g) in the positive mode (*n* = 5). Features are presented as peak intensities normalized to sample weight and log₁₀-transformed. Compounds were subsequently identified by comparison of mass spectra and retention times to those of authentic standards. The piceatannol dimer is langusol C, and the resveratrol dimers are *E*-δ-viniferin and *Z*-δ-viniferin. ***E*-1a** and ***Z*-1a** denote the *E*- and *Z*-isomers, respectively. Detailed metabolomic results are provided in the raw data file, and representative mass spectra of phenolic compounds and authentic standards are shown in Supplementary File 2. Statistical significance between groups was assessed using two-tailed *t*-tests (*P* < .05) in c–g.

The fungus *C. radians* exhibited contrasting behavior in that, although glucoside levels were significantly reduced following incubation, the corresponding aglucones did not accumulate to detectable levels. Instead, a strongly induced feature at *m*/*z* 359.11287 (negative mode, RT 5.71 min) was detected in the extract of the culture medium after incubation with *C. radians*. MS/MS analysis revealed a fragment corresponding to resveratrol (**3b**) (*m*/*z* 227.0719, negative mode) and a neutral loss of C_5_H_8_O_4_ (Fig. 2c and Fig. 3c). Similarly, a significantly increased feature at *m*/*z* 375.10887 (negative mode, RT 5.44 min), yielding a piceatannol fragment (*m*/*z* 243.0660) and the same C_5_H_8_O_4_ neutral loss, was detected following incubation with *C. ipidophilum* (Fig. 2d and Fig. 3a). These data suggest the conjugation of a pentose moiety (C_5_H_10_O_5_) to phenolic aglucones by both *C. radians* and *C. ipidophilum*. Both species belong to the order Agaricales, and this modification was not observed in the other fungal taxa examined. Analyses conducted on fungal biomass incubated on PDA medium compared to SB-PDA confirmed that these new features were derived by *C. radians* and *C. ipidophilum* transformation of chemical compounds from spruce bark and not from de novo biosynthesis (Fig. S3a).

**Figure 3:**
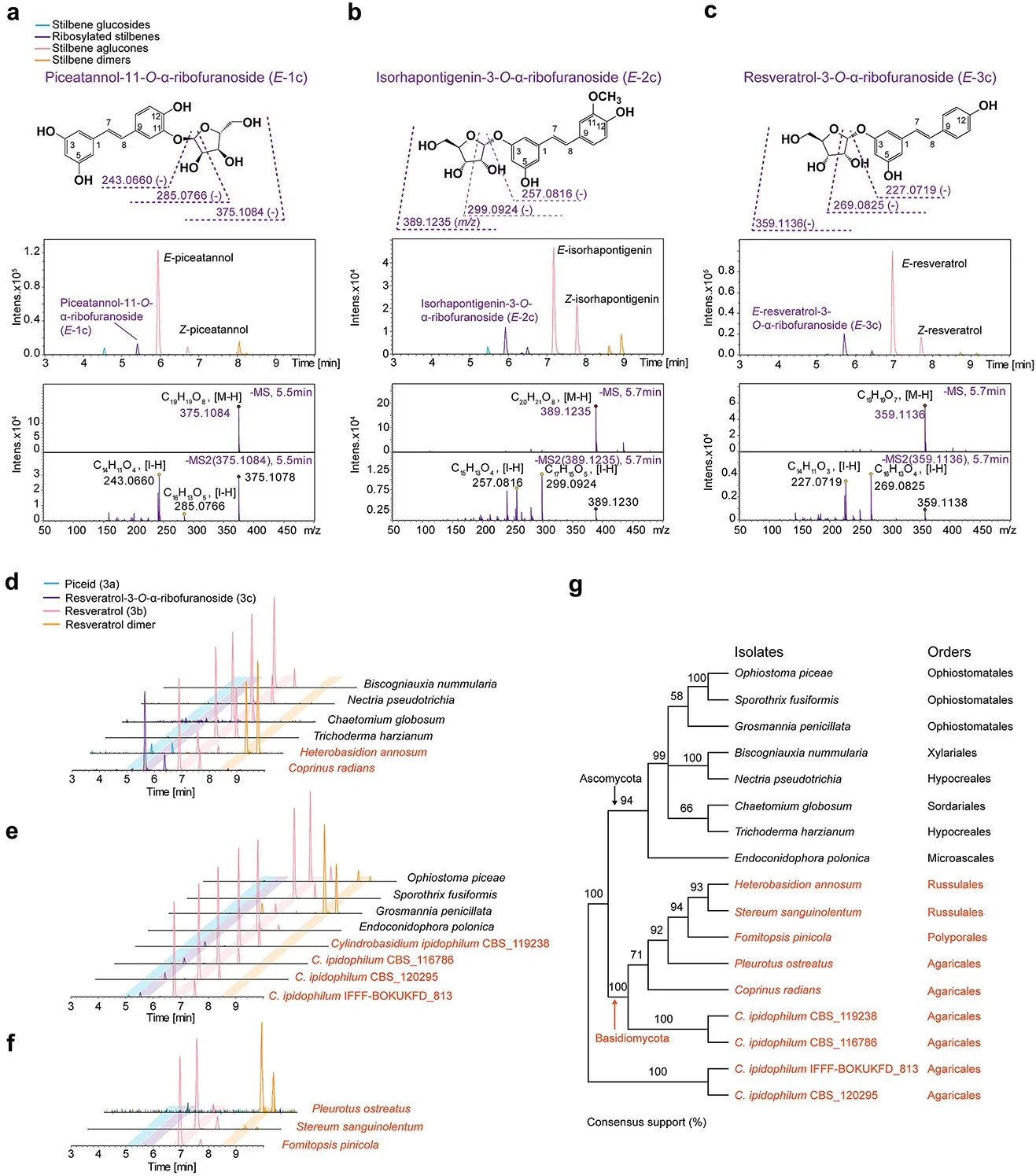
Ribosylated stilbenes occur in two Basidiomycota species of spruce tree- and bark beetle–associated fungi. (a–c) Confirmed structures of ribosylated derivatives of piceatannol (a), isorhapontigenin (b), and resveratrol (c). Depicted are extracted ion chromatograms and mass spectra from untargeted UHPLC–qTOF–MS analyses performed in negative ionization mode for glucosides and their derivatives. Compounds are from the incubation of *C. radians* with the corresponding stilbene glucosides. NMR spectra and assignments are shown in Supplementary Fig. S2 and Table S4. (d–f) Screening of a range of other fungal species by UHPLC–qTOF–MS for piceid metabolites. Fungi are spruce tree associates (d), *I. typographus* symbionts (e), or represent three additional Basidiomycota strains (f). Extracted ion chromatograms obtained in the negative ionization mode. Fungal species belonging to Ascomycota are shown in black, and Basidiomycota in orange. Representative mass spectra of phenolic standards are shown in Supplementary File 2. (g) Phylogenetic analysis of fungal isolates. ITS region sequences were aligned using MUSCLE, and a UPGMA tree was generated in Geneious prime. Branch labels indicate consensus support (%), and ITS rRNA gene sequences are provided in Supplementary File 1.

To further verify the formation of these pentose-conjugated metabolites from stilbene aglucones, fungal cultures were incubated with resveratrol and [^13^C_6_] resveratrol, followed by comparative untargeted metabolomic analysis. In both *C. radians* and *C. ipidophilum*, incubation with labeled substrate resulted in the expected isotopic shift of the pentose-conjugated product, with a significant increase of the *m*/*z* 359.11287 feature in cultures supplied with unlabeled resveratrol and a corresponding increase of the isotopically shifted feature at *m*/*z* 365.13365 in cultures supplied with [^13^C_6_] resveratrol (Fig. 2e and f). Most of the resveratrol metabolites formed by *C. radians* were pentose-conjugated derivatives or resveratrol dimers, as indicated by both negative and positive ionization modes (Fig. 2e and g).

### Ribosylation of spruce stilbenes by *Coprinellus radians* and *Cylindrobasidium ipidophilum*

The pentose-conjugated stilbene metabolites were next isolated from fungal cultures incubated with astringin (**1a**), isorhapontin (**2a**), and piceid (**3a**), and their structures were elucidated by mass spectrometry and NMR spectroscopy (Fig. 3a–c, Fig. S2, and Table S4). In *C. radians*, the 11-hydroxyl group of piceatannol was substituted with an α-ribose moiety to give piceatannol-11-*O*-α-ribofuranoside (**1c**) (Fig. 3a and Fig. S3b). Likewise, both *C. radians* and *C. ipidophilum* had α-ribose substitutions at the 3-hydroxyl position of isorhapontigenin and resveratrol to form isorhapontigenin-3-*O*-α-ribofuranoside (**2c**), and resveratrol-3-*O*-α-ribofuranoside (**3c**), respectively (Fig. 3b and c and Fig. S3b and c). In addition, *C. ipidophilum* produced resveratrol-3,5-*O*-α-bisribofuranoside (**3d**) (Fig. S3d). Three of these ribosides (**1c**, **2c**, **3d**) are new and were identified by their mass and NMR spectra (Fig. S2, Table S4, and Supplementary File 2). The fourth riboside (**3c**) was previously described [39] and was identified here by comparison of our spectral data with those in the literature.

We looked for ribosylated stilbene derivatives in a range of other fungal species cultivated under the same conditions using piceid (**3a**) as substrate because of its greater stability in the fungal medium compared to the other stilbene glucosides (Fig. S4). Ribosylated products were detected in the saprotroph *C. radians*, but not in the saprotrophs *Chaetomium globosum*, *Nectria pseudotrichia*, and *Trichoderma harzianum*, the pathogen *H. annosum*, nor the endophyte *B. nummularia* under the conditions used (Fig. 3d). Stilbene ribosylation was also consistently observed in multiple *C. ipidophilum* strains (Fig. 3e). By contrast, other *I. typographus*–associated fungi, including *E. polonica*, *G. penicillata*, *Sporothrix fusiformis*, and *Ophiostoma piceae*, did not exhibit such ribosylated derivatives when incubated with piceid (**3a**) (Fig. 3e). Both *C. radians* and *C. ipidophilum*, which exhibited ribosylation activity, belong to the Basidiomycota, order Agaricales (Fig. 3g). To test the ability of other Basidiomycota species to metabolize stilbenes in the manner, we tested the pathogen *Stereum sanguinolentum* (Russulales), and the saprotrophs *Fomitopsis pinicola* (Polyporales) and *P. ostreatus* (Agaricales)*.* However, these species failed to generate ribosylated phenolic metabolites under the same experimental conditions (Fig. 3f and g).

To quantify the extent of α-riboside formation, targeted metabolite analyses were conducted after incubating fungi with stilbene glucosides in PDA medium. Owing to the instability of astringin (**1a**) in PDA during incubation (Fig. S4), only the glucosides isorhapontin (**2a**) and piceid (**3a**) were used. Following incubation of *C. radians* with 300 μM isorhapontin and piceid, most of these compounds were converted to their corresponding aglucones, which accumulated in fungal biomass at ~1.5 μmol g^−1^ isorhapontigenin (**2b**) and 3.1 μmol g^−1^ resveratrol (**3b**), respectively (Fig. 4a and b). The ribosylated metabolites isorhapontigenin-3-*O*-α-ribofuranoside (**2c**) and resveratrol-3-*O*-α-ribofuranoside (**3c**) were also detected in the biomass at ~0.6 and 0.5 μmol g^−1^, respectively, representing substantially lower levels than the corresponding aglucones (Fig. 4a and b). Both aglucones and ribosylated derivatives accumulated preferentially in the mycelia rather than in the culture medium of *C. radians* after fungal growth (Fig. S5a). The fungus *C. ipidophilum* produced ~0.02 and 0.4 μmol g^−1^ of the ribosylated metabolites isorhapontigenin-3-*O*-α-ribofuranoside (**2c**) and resveratrol-3-*O*-α-ribofuranoside (**3c**), respectively in fungal biomass (Fig. 4c and d), and these metabolites were nearly undetectable in the corresponding culture media (Fig. S5b). Together, these results demonstrate that α-ribosylation of spruce phenolic aglucones isorhapontigenin (**2b**) and resveratrol (**3b**) represents a species-specific metabolic capability, prominently expressed in *C. radians* and, to a lesser extent, in *C. ipidophilum* under the culture conditions used.

**Figure 4:**
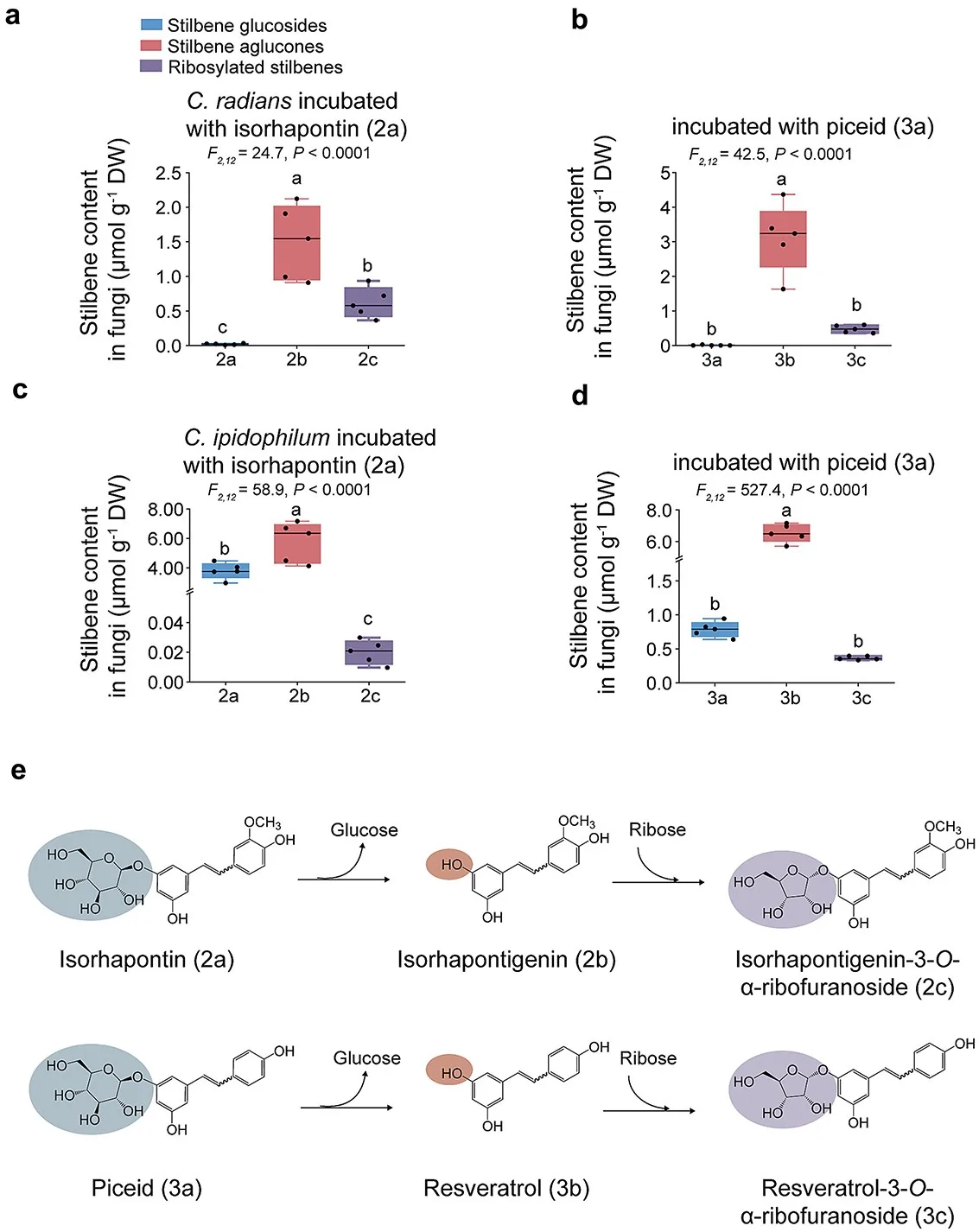
Biotransformation of stilbene glucosides by *C. radians* and *C. ipidophilum.* (a and b) quantification of stilbene glucosides, aglucones, and ribosylated derivatives in the fungal biomass of *C. radians* after 4 days of incubation with the glucosides isorhapontin (a) and piceid (b) (*n* = 5). (c and d) Quantification of stilbene metabolites in the fungal biomass of *C. ipidophilum* after 4 days of incubation with isorhapontin (c) and piceid (d) (*n* = 5). The amounts of *E-* and *Z-* isomers were combined, e.g. compound **2a** represents the combined quantity of ***E*-2a** and ***Z*-2a**. Box plots represent the range of 25 to 75%, with center lines indicating median values and whiskers extending to 1 time the interquartile range. (e) Proposed metabolic pathway illustrating the hydrolysis of stilbene glucosides (isorhapontin and piceid) to their corresponding aglucones, followed by ribosylation. Statistical significance among treatments was assessed using one-way ANOVA followed by Fisher’s LSD tests in a–d (*P* < .05). Lowercase letters indicate statistically significant differences.

### Reduced toxicity of stilbene glucosides and ribosides compared to aglycones

Sugar conjugation increases polarity and can attenuate the biological activity of phenolic compounds. To assess the functional consequences of ribosylation, we compared the effects of stilbene glucosides, their corresponding aglycones, and ribosylated derivatives on the growth of *C. radians* and *C. ipidophilum*. The aglycones isorhapontigenin (**2b**) and resveratrol (**3b**) inhibited fungal growth in a dose-dependent manner in both species (Fig. 5a–d). The stilbene glucosides and the ribosylated derivatives, in contrast, exhibited markedly weaker inhibitory effects. The IC_50_ values for the glucoside isorhapontin (**2a**) and ribosylated isorhapontigenin (**2c**) were over 800 μM, while the IC_50_ for the corresponding aglucone isorhapontigenin (**2b**) was 365 and 545 μM for *C. radians* and *C. ipidophilum*, respectively (Table 1). Although both ribosylated compounds reduced *C. radians* growth at 800 μM compared to the control without stilbenes, growth remained substantially higher than in cultures treated with the aglycones, which showed 85% and 65% inhibition compared to the non-stilbene control for isorhapontigenin (**2b**) and resveratrol (**3b**), respectively (Fig. 5a and b and Table 1). Neither the ribosylated isorhapontigenin (**2c**) nor the ribosylated resveratrol (**3c**) significantly affected the growth of *C. radians* and *C. ipidophilum* at the tested concentrations. IC_50_ values for these compounds were either higher than 800 μM or could not be reliably estimated because the dose–response data could not be adequately fitted to a nonlinear model (Fig. 5a–d and Table 1). Moreover, *C. radians* displayed improved growth in the presence of ribosylated resveratrol (**3c**) compared with both the glucoside piceid (**3a**) and the aglycone resveratrol (**3b**) at equivalent concentrations (Fig. 5b). Additionally, the effect of stilbene aglucones on the growth of *H. annosum*, which does not carry out ribosylation, was greater than that of the corresponding ribosylated derivatives (Fig. 5e and f). The IC_50_ of ribosylated resveratrol (**3c**) (713 μM) for *H. annosum* was much higher than that for resveratrol (**3b**) (101 μM), and growth was much less inhibited by ribosylated resveratrol (**3c**) at 800 μM (34%) than for resveratrol (**3b**) (74%) at this concentration (Table 1). Overall, these findings demonstrate that ribosylation substantially reduces the antifungal activity of stilbene aglucones toward the tested fungal species, supporting a role for α-ribose conjugation in mitigating stilbene toxicity.

**Figure 5:**
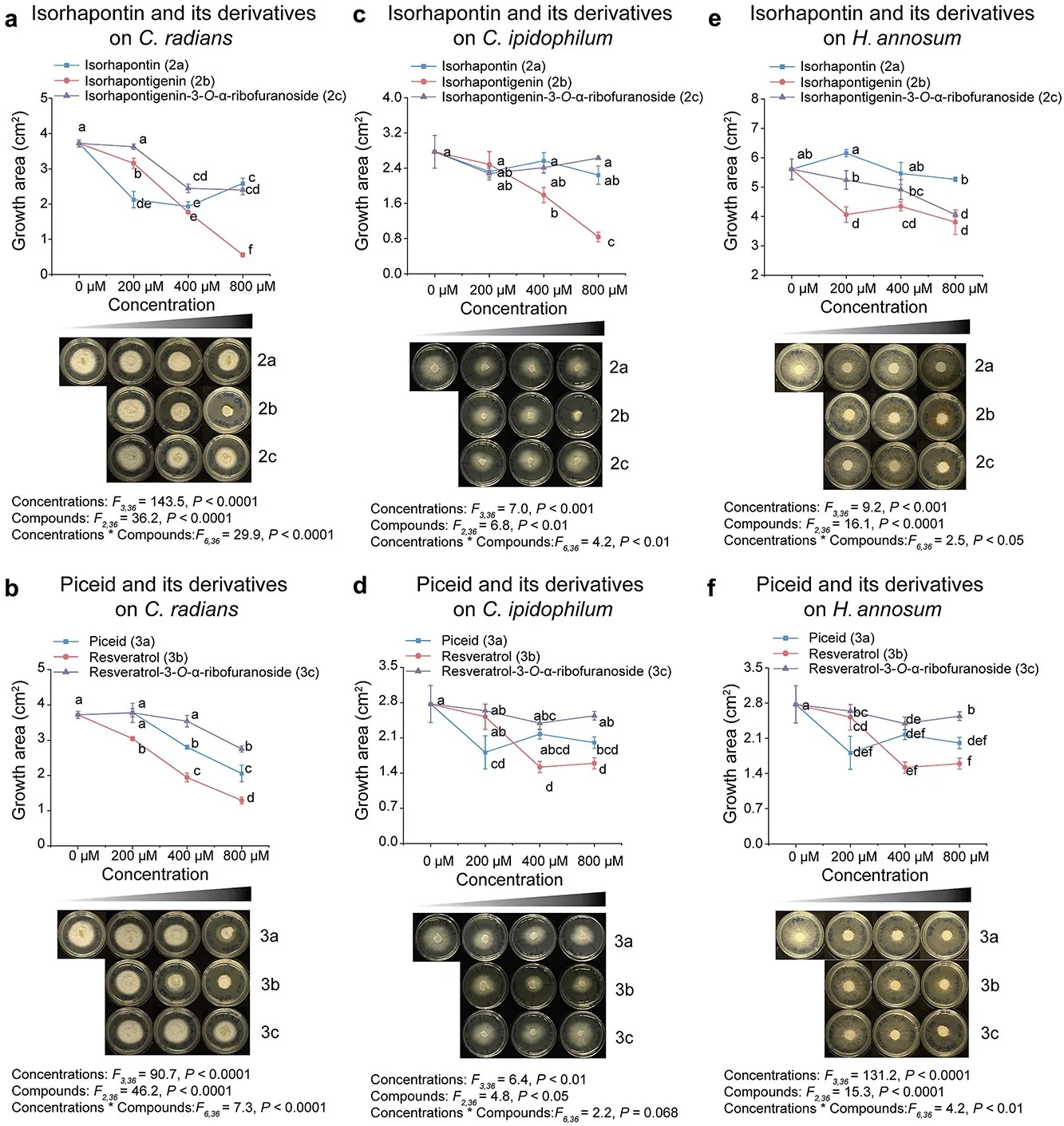
Stilbene glucosides and ribosides exhibit lower toxicity to fungi than the corresponding aglycones. (a and b) Colony growth area of *C. radians* on PDA plates supplemented with increasing concentrations of isorhapontin and its derivatives (a), and piceid and its derivatives (b) (*n* = 4). (c and d) Colony growth area of *C. ipidophilum* on PDA plates supplemented with increasing concentrations of isorhapontin and its derivatives (c), and piceid and its derivatives (d) (*n* = 4). (e and f) Growth area of *H. annosum* on PDA plates supplemented with increasing concentrations of isorhapontin and its derivatives (e) or piceid and its derivatives (f) (*n* = 4). Statistical significance among treatments was assessed using two-way ANOVA followed by Fisher’s LSD tests (*P* < .05). Data are presented as mean ± SE. Lowercase letters indicate statistically significant differences.

**Table 1:** Glucosylated and ribosylated phenolics exhibit reduced antifungal activity compared with their corresponding aglycones. IC_50_ values (mean ± SE) were determined by nonlinear curve fitting using a biphasic dose–response function. Percent inhibition (mean ± SE) was calculated based on fungal growth area at 800 μM relative to the control group (*n* = 4 per treatment). IC_50_ values exceeding the highest concentration tested (800 μM) are shown as “> 800”. The original data used for IC_5_₀ calculations are provided in the Raw Date File. “NF” indicates that the data could not be adequately fitted to the nonlinear dose–response model.

	*Coprinellus radians*		*Cylindrobasidium ipidophilum*		*Heterobasidion annosum*	
Substrates	IC_50_ (μM)	Inhibition (%) at 800 (μM)	IC_50_ (μM)	Inhibition (%) at 800 (μM)	IC_50_ (μM)	Inhibition (%) at 800 (μM)
Isorhapontin (**2a**)	> 800	30.4 ± 3.9	> 800	19.1 ± 7.5	NF	6.1 ± 1.3
Isorhapontigenin (**2b**)	365.4 ± 31.0	85.1 ± 1.5	545.1 ± 349.5	69.7 ± 3.9	> 800	32.2 ± 7.4
Isorhapontigenin-3-*O*-ribofuranoside (**2c**)	> 800	35.5 ± 3.7	> 800	5.1 ± 1.1	> 800	27.6 ± 1.4
Piceid (**3a**)	> 800	44.8 ± 6.3	> 800	27.6 ± 4.1	238.5 ± 25.0	66.8 ± 2
Resveratrol (**3b**)	470.1 ± 48.7	65.3 ± 2.4	> 800	42.4 ± 3.9	101.5 ± 15.4	73.6 ± 3.2
Resveratrol-3-*O*-ribofuranoside (**3c**)	> 800	26.0 ± 2.3	NF	8.4 ± 3.1	712.9 ± 303.9	34.2 ± 5.4

### Increased metabolic stability of stilbene ribosylated derivatives compared to glucosides

To determine whether conjugation with a non-glucose sugar moiety confers resistance to enzymatic hydrolysis, we performed *in vitro* assays using stilbene glucosides and α-ribosides as substrates. Crude protein extracts from *C. radians* efficiently hydrolyzed the glucosides isorhapontin (**2a**) and piceid (**3a**) to their corresponding aglycones isorhapontigenin (**2b**) and resveratrol (**3b**) (Fig. 6a and b). In contrast, the same extracts did not hydrolyze the ribosylated derivatives isorhapontigenin-3-*O*-α-ribofuranoside (**2c**) and resveratrol-3-*O*-α-ribofuranoside (**3c**), indicating resistance to fungal glycosidase activity (Fig. 6a and b). A similar pattern was observed with crude protein extracts from *C. ipidophilum*. The glucosides isorhapontin (**2a**) and piceid (**3a**) were readily converted to aglycones, whereas the ribosylated derivatives isorhapontigenin-3-*O*-α-ribofuranoside (**2c**) and resveratrol-3-*O*-α-ribofuranoside (**3c**) remained intact (Fig. 6c and d). Moreover, protein extracts from *I. typographus*, which possess considerable hydrolytic activity toward stilbene glucosides [38], were likewise unable to hydrolyze ribosylated stilbenes (Fig. S6). Together, these results indicate that α-ribosylation stabilizes stilbenes against enzymatic cleavage by both fungal and bark beetle glucosidases, thereby preventing reformation of the toxic aglycone form.

**Figure 6:**
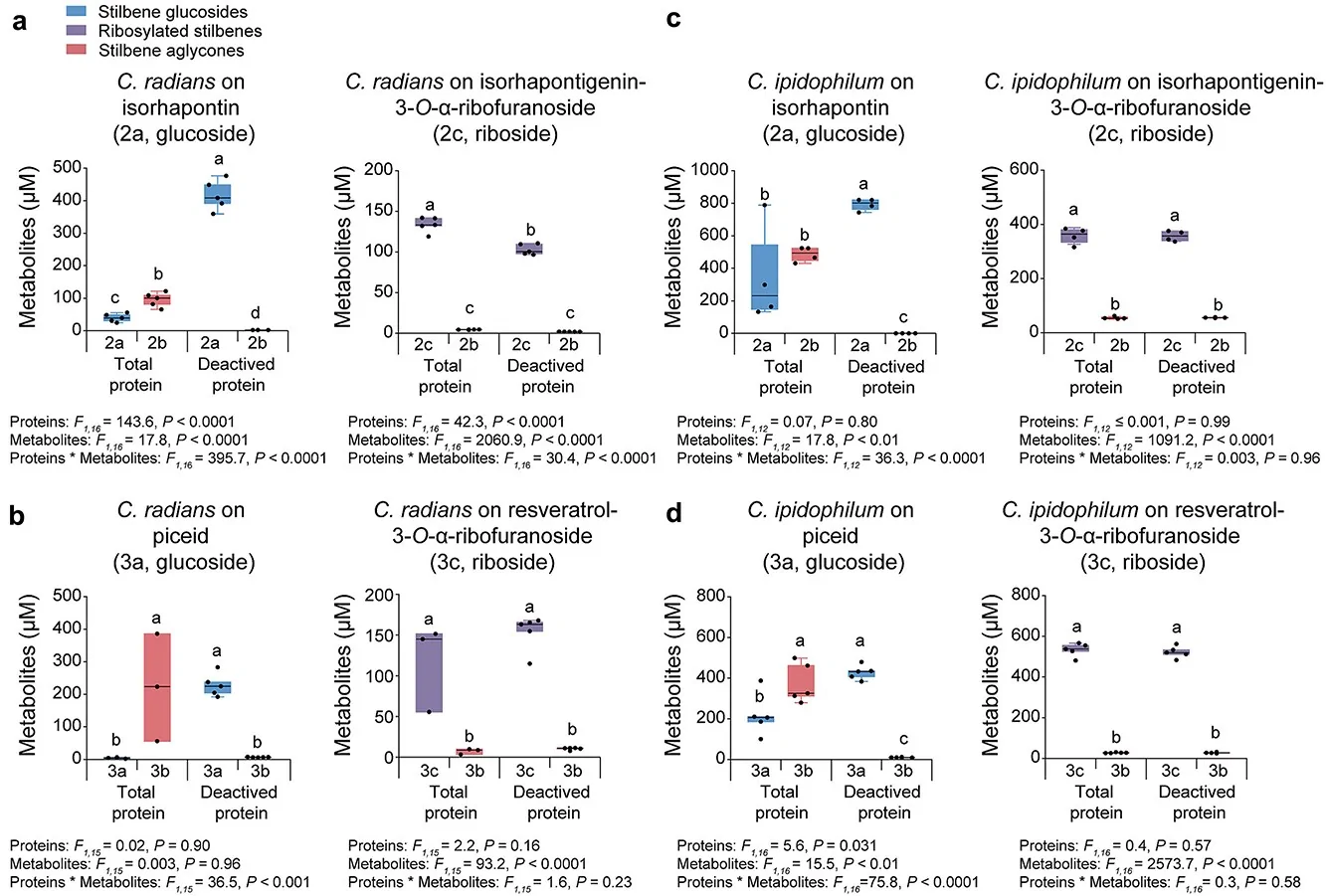
Ribosylation of stilbene aglycones increases stability against fungal hydrolysis compared to glucosylation. (a and b) Crude protein extracts from *C. radians* were incubated with the stilbene glucosides isorhapontin and its ribosylated derivative isorhapontigenin-3-*O*-ribofuranoside (a), and with piceid (glucoside). And its ribosylated derivative resveratrol-3-*O*-ribofuranoside (b). (c and d) Crude protein extracts from *C. ipidophilum* were incubated with isorhapontin (glucoside) and its ribosylated derivative (c), and with piceid (glucoside) and its ribosylated derivative (d), respectively. Boiled, heat-inactivated protein extracts served as negative controls (*n* = 3–5). The amounts of *E-* and *Z-* isomers were combined, e.g. compound **2a** represents the combined quantity of ***E*-2a** and ***Z*-2a**. Statistical significance among treatments was assessed using two-way ANOVA followed by Fisher’s LSD tests (*P* < .05). Lowercase letters indicate statistically significant differences.

### Increased transformation of stilbene aglycones to α-ribosides by fungal species cultured on spruce bark-containing medium correlates with elevated fungal growth

To further address the biological significance of α-ribosylation, we compared the magnitude of fungal growth on PDA medium containing spruce bark powder (0%, 0.5%, 1% or 2%) with the extent of α-ribosylation. The experiment was conducted on several species of the Basidiomycota used in this study: the Norway spruce associate, *C. radians*, the bark beetle symbiont, *C. ipidophilum*, and a saprophyte found in many tree species, *P. ostreatus*. While the growth of *C. ipidophilum* increased substantially with spruce bark content in this range, the growth of the other two species was reduced with increasing spruce bark content (Fig. 7a–c). The formation of ribosylated stilbenes, quantified by targeted LC–MS/MS analysis, was highest in *C. ipidophilum*, especially for piceatannol-11-*O*-α-ribofuranoside (**1c**) (Fig. 7d and e). These results suggest that the biotransformation of stilbenes by fungi may contribute to improved growth in spruce trees.

**Figure 7:**
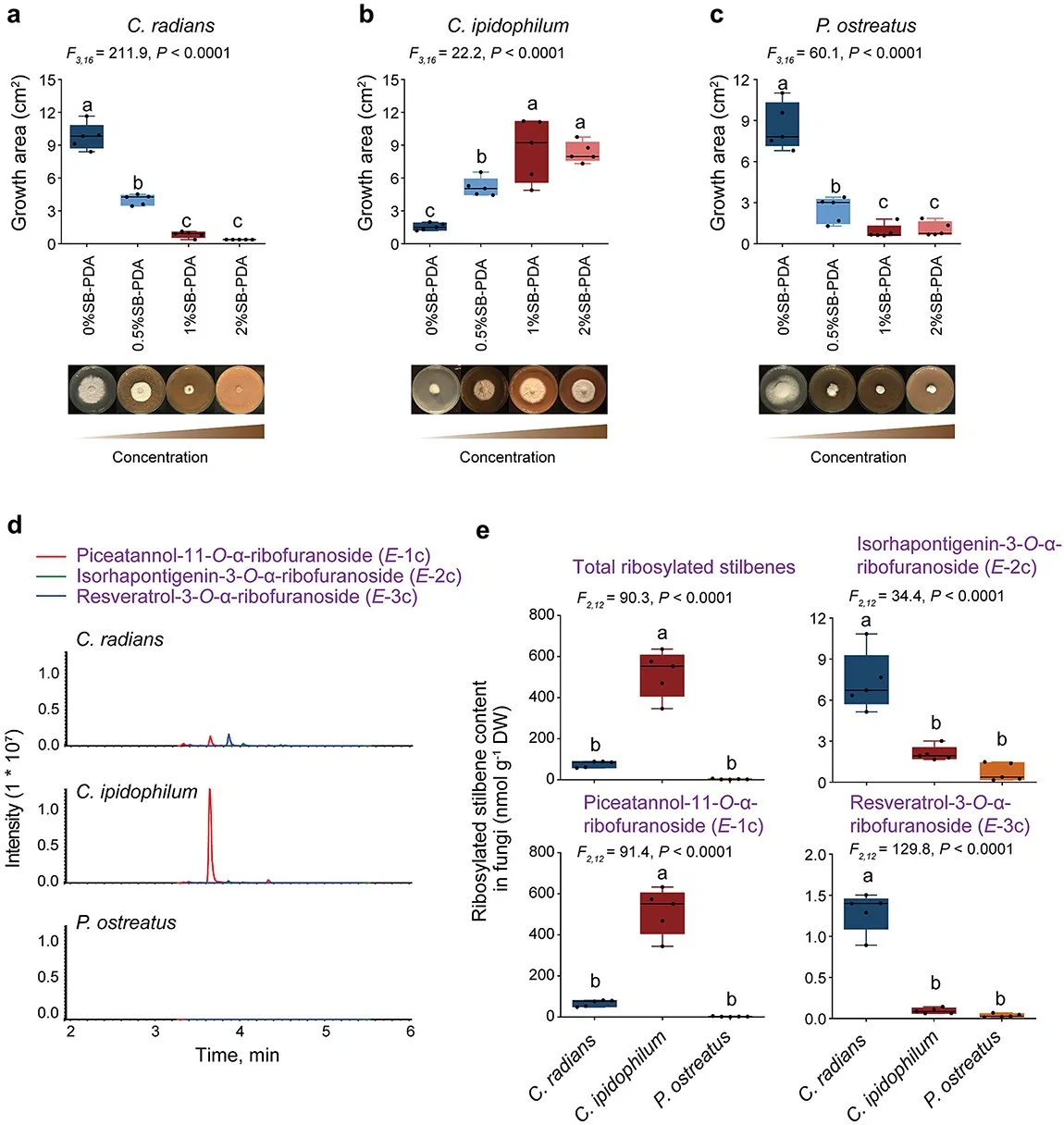
Increased metabolism of stilbene aglucones to α-ribosylated derivatives is correlated with enhanced fungal growth on spruce bark-containing medium: comparison of three Basidiomycota species. (a–c) Growth area of *C. radians* (a), *C. ipidophilum* (b), and *P. ostreatus* (c) grown on PDA plates supplemented with 0%, 0.5%, 1%, or 2% spruce bark (*n* = 5). (d) Extracted ion chromatograms of ribosylated stilbenes in fungal biomass obtained by targeted LC–MS/MS analysis performed in negative ionization mode. (e) Quantification of total ribosylated stilbenes, piceatannol-11-*O*-α-ribofuranoside, isorhapontigenin-3-*O*-α-ribofuranoside, and resveratrol-3-*O*-α-ribofuranoside in fungal biomass grown on 1% SB-PDA (*n* = 5). Statistical significance among treatments was assessed by one-way ANOVA followed by Fisher’s LSD test (*P* < .05) for panels a–c and e. Different lowercase letters indicate statistically significant differences.

## Discussion

Glycosylation is a widespread strategy in plants for the storage and regulation of chemical defenses, because it modifies the solubility, stability, and bioactivity of aglucones [40]. This chemical modification enables high intracellular accumulation of defense compounds while minimizing autotoxicity, yet plants retain the capacity for rapid activation upon biotic attack through enzymatic hydrolysis [41]. In this study, the phloem (inner bark) of Norway spruce (*P. abies*) trees was found to contain abundant stilbene- and flavonoid-based, anti-microbial defenses, which occur predominantly as glucosides. These greatly exceeded the concentrations of their corresponding aglucones in uninfected phloem tissues (Fig. 1) [38]. A similar chemical defense architecture has been reported in other conifer species [42, 43].

Two types of biotic attack caused a pronounced depletion of stilbene and flavonoid glucosides in phloem tissues and the accumulation of the corresponding aglucones, changes not observed after mechanical wounding (Fig. 1 and Fig. S1). Both the invasion of *I. typographus* bark beetles and their symbiotic fungi, as well as the infection of Norway spruce–associated fungi not symbiotic with bark beetles caused similar metabolic changes. Bark beetle fungal symbionts have been extensively studied, particularly the ophiostomatoids, which may support beetle invasion by providing nutrients to bark beetles [44, 45], detoxifying plant defense compounds [26, 46] and hastening the death of the tree [12, 47]. Beetle symbionts from the Basidiomycota have been comparatively less investigated, but are thought to have similar ecological roles [14]. Fungi inhabiting the phloem of conifer trees even in the absence of bark beetle invasion may also impact tree-beetle interactions following invasion. For instance, pathogenic fungi can weaken host defenses [27], which may facilitate beetle invasion. Saprotrophs, which obtain nutrients by breaking down dead organic matter, can contribute to nutrient cycling in habitats such as bark beetle galleries [48]. Endophytes exhibit diverse roles [49], including the potential to enhance host tree resistance despite causing no visible symptoms. In this study, we investigated both *I. typographus* symbiotic fungi introduced during beetle invasion, and fungi isolated from uninvaded Norway spruce phloem encompassing a range of ecological strategies. All cultured isolates metabolized the phenolic glucosides and the majority showed an increase in the aglucones, including various saprotrophs, pathogens, and endophytes, and some bark beetle symbionts (Fig. 2). Hydrolysis of stilbene and flavonoid glucosides has been well documented in previous work on bark beetle–associated fungi [25, 50] and the bark beetles themselves [38].

Because stilbene and flavonoid aglycones generally exhibit stronger antifungal activity than their glucosides [51, 52], such hydrolysis would be expected to inhibit fungal growth. Indeed, increasing aglucone concentrations in growth media significantly inhibited all the fungal species tested here, including the *I. typographus* symbiont *C. ipidophilum*, the saprotroph *C. radians*, and the pathogen *H. annosum* (Fig. 5). These results support the concept that Norway spruce stores anti-fungal “protoxins” that are converted into toxic derivatives upon fungal colonization. Plants store many other classes of defense compounds as glucoside protoxins, such as cyanogenic glucosides, benzoxazinoids, iridoid glucosides, and glucosinolates [53–56]. These protoxins are accompanied in the plant by designated glucoside hydrolases, which contact their glycoside substrates upon plant damage, catalyzing their conversion to toxic products [41]. This type of activated defenses allows the plant to respond immediately to tissue rupture. However, it can be circumvented by insects that avoid tissue disruption, such as piecing-sucking aphids [57]. In contrast, spruce stilbene and flavonoid glucosides appear to be hydrolyzed by the infecting fungi themselves to yield the toxic aglucones (Fig. S1). Thus, these defenses are not activated by simple mechanical damage, which minimizes the risk of autotoxicity for the plant. However, they require that attackers possess their own β-glucosidases, which means that they may not be effective against all enemies.

The accumulation of stilbene and flavonoid aglucones in fungus-infected and bark beetle-colonized phloem was substantially lower than would be expected based on the extent of glucoside depletion (Fig. 1 and Fig. S1). This discrepancy suggests that additional metabolic transformations might occur in parallel with or subsequent to deglycosylation. Stilbene aglucones, for example, are known to undergo diverse modifications, including methylation, glycosylation, glucuronidation, sulfation, dimerization, and oxidative cleavage [47, 58], yet such processes have been little explored in the fungi associated with spruce trees and their bark beetles despite the fact that these microbes are confronted with some of the highest concentrations of stilbenes known in plant tissues [59]. So far, *E. polonica*, a *I. typographus* symbiont, has been shown to transform stilbenes into muconoid-type ring-cleavage products, which are likely the first steps in the degradation of spruce defenses into substrates that can enter the tricarboxylic acid cycle and be used as carbon resources [26]. Another case of phenolic metabolism in spruce-bark beetle-microbe interactions is the metabolism of *I. typographus*-derived phenolic aglucones by the entomopathogen *B. bassiana* into methylglucosylated phenolic derivatives as a detoxification strategy [38].

Other organisms besides fungi could also metabolize spruce-derived phenolic compounds. Bark beetle galleries contain a complex community in addition to fungi and bacteria, including nematodes, and mites [10, 60, 61], that could also metabolize phenolics. For example, the two-spotted spider mite (*Tetranychus urticae*), is reported to glycosylate the tomato flavonoid (*S*)-naringenin via a UDP-glycosyltransferase [62]. Nematodes of the genus *Heterorhabditis* and their *Photorhabdus luminescens* bacteria can detoxify stilbenes via epoxidation [63]. Additionally, some phenolic compounds, such as astringin and piceatannol, may undergo spontaneous degradation (Fig. S4), influenced by pH and their chemical structure [64, 65]. These biotic and abiotic pathways could jointly explain the observed depletion of stilbene glucosides in *I. typographus*-infested Norway spruce phloem without a corresponding accumulation of aglucones (Fig. 1 and Fig. S1).

In this study, we identified α-ribosylation as a previously unrecognized metabolic fate of spruce stilbenes. We demonstrated that two Basidiomycota of the order Agaricales, the Norway spruce–associated saprotrophic fungus *C. radians* and the spruce bark beetle–associated saprotroph *C. ipidophilum* both convert the three major stilbene aglucones of Norway spruce into three mono-α-ribofuranosylated derivatives and one bi-α-ribofuranosylated compound (Fig. 3 and Fig. S3). Ribosylation of plant metabolites and other xenobiotics is rare in fungi and other microbes, with only a few cases documented previously [39, 66]. However, α-ribosylation activity was conserved across multiple *C. ipidophilum* strains but was absent from other *I. typographus* symbionts, including *E. polonica* and *G. penicillata* (Fig. 3e). Among fungi associated with Norway spruce unattacked by bark beetles, only the Agaricales species *C. radians* exhibited ribosylation activity in our study (Fig. 3d). The saprotroph *P. ostreatus (Agaricales) could not transform phenolic aglucones into ribosylated derivatives.* The wood-decaying saprotrophic fungus *Daldinia eschscholzii* (Xylariales) has been reported to α-ribosylate resveratrol [39], but a related species from the same order, the endophyte *B. nummularia* (Xylariales), lacked this activity (Fig. 3d). This pattern suggests that ribosylation of xenobiotics may represent a lineage-specific detoxification strategy linked to particular taxonomic groups. However, broader sampling across fungal taxa is required to test these hypotheses. In particular, additional strains from Agaricales and Xylariales are needed to assess the phylogenetic specificity of phenolic ribosylation, while a wider range of tree-associated saprotrophs should be examined to evaluate its potential niche specificity. These experiments should ideally be conducted on a range of media, especially with lower nutrient content, to see if these conditions induce ribosylation or other phenolic detoxification pathways.

Norway spruce– or bark beetle–associated fungi that do not ribosylate stilbenes must have other metabolic adaptations to cope with these toxins or resist them by another mechanism, such as target site insensitivity or rapid excretion [67]. For example, the spruce pathogen *H. annosum* (Russulales) has been reported to transform phenolics into dimers via a laccase activity [68], although the toxicity of the resulting products remains unknown. In this study, we found that *H. annosum*, the saprotroph *P. ostreatus*, and the *I. typographus*–associated symbiont *G. penicillata* (Ophiostomatales) all converted piceid via resveratrol to resveratrol dimers (Fig. 3f). Therefore, it is difficult to draw conclusions about the species-specificity or niche-specificity of dimerization. The dimerization of spruce phenolics by more fungi needs to be examined, and pathways besides ribosylation and dimerization need to be explored. Our results demonstrated unequivocally that α-ribosylation reduces stilbene toxicity. Ribosylated stilbenes lacked the strong growth-inhibitory effects exhibited by their aglycone counterparts, allowing both *C. radians* and *C. ipidophilum* to maintain robust growth in their presence (Fig. 5). Glycosylated stilbenes in general are known to lack many of the biological and pharmacological activities of their corresponding aglycones [69]. Similar detoxification reactions are reported in fungi for other plant phenolic toxins, such as xylosylation, glucosylation, and methyl-glucosylation [32, 34, 38]. Glycosylation of ingested toxins is also an important detoxification strategy for insect herbivores [29, 30, 70], plant-feeding mammals and in fact all organisms encountering lipophilic toxins [28, 31, 33]. Glycosylation enlarges the mass of the compound and increases its polarity, and this may prevent the modified toxin from reaching the active site, which for stilbenes might be the cell wall, plasma membrane, nucleus or mitochondria at the subcellular level [59].

α-Ribosylation of stilbenes not only decreases their toxicity, but increases their metabolic stability, providing resistance to hydrolysis by fungal and *I. typographus* protein extracts that were able to hydrolyze glucosylated stilbenes (Fig. 6 and Fig. S6). Conjugation with non-glucose sugars has been shown to block β-glucosidase-mediated enzyme cleavage in other systems [30, 34, 35]. For example, methylation of the glucose moiety in *B. bassiana* stabilizes phenolic glucosides, rendering them resistant to hydrolysis by insect, fungal, and commercial β-glucosidases [38]. In environments where β-glucosidase activities are prevalent, the reaction of aglycone toxins with unusual sugars may increase their stability and prevent reappearance of the unprotected toxin. In this study, we demonstrated the benefits of α-ribosylation by comparing the growth of two ribosylating Basidiomycota species, and one non-ribosylating Basidiomycota on spruce bark-containing medium. Under these conditions, the species with the highest ribosylation capacity achieved the highest growth rate (Fig. 7).

Despite the advantages of producing ribosylated stilbenes, these compounds accumulated at lower levels than their corresponding aglycones in the fungal species studied here (Fig. 4). α-Ribosylation may be constrained by the supply of this pentose sugar because ribose is also required for nucleotide biosynthesis and other critical cellular processes [71, 72]. Thus, this detoxification process may lead to significant trade-offs with fungal growth and development. Further experiments with loss-of-function strains will be useful to prove the function of ribosylation for these fungi. It is also likely that the a-ribosylating taxa and other fungi studied here possess additional ways to resist toxic phenolic compounds besides metabolism. Rapid excretion via efflux transporters is possible, and ABC transporters are known to excreted compounds like the stilbene resveratrol from fungal cells [67, 73]. However, our data suggest that transporter-mediated export is unlikely to be a predominant detoxification mechanism in species studied. Phenolic aglycones were primarily detected in fungal biomass rather than in the surrounding medium. For example, for *C. ipidophilum* incubated with 600 μM piceid in PDA medium, ~6 μmol/g dry weight of phenolic aglycones accumulated in fungal biomass (Fig. 4), whereas a much smaller proportion of these substances (about 0.25 μmol/g fresh weight) was detected in the culture medium (Fig. S5). Nevertheless, the potential contribution of ABC and MFS transporters to fungal tolerance of spruce defense compounds remains an important topic for future investigation.

In conclusion, many species of fungi colonize the phloem (inner bark) of Norway spruce trees, either as saprotrophs, pathogens and endophytes, or as symbionts of invading bark beetles. This abundance seems surprising given the high concentrations of stilbene glucosides present in the phloem that are hydrolyzed to antifungal aglucones upon initial fungal infestation and bark beetle attack. Here we describe how two species of Basidiomycota convert stilbene aglucones to less toxic and metabolically stable α-riboside derivatives. Elucidation of this biochemical process provides insight on what facilitates successful colonization of chemically-defended tissues in woody plants. In addition, because one of the α-ribosylating fungi is an associate of a tree-killing spruce *I. typographus*, which might support beetle invasion of Norway spruce by providing nutrients or overcoming tree defenses, its ability to form α-ribosylated stilbenes may help promote beetle outbreaks.

## Supplementary Material

Supplementary_File_1_wrag230

Supplementary_File_2_wrag230

ISME-Supplementary_figures-R2_wrag230

ISME-Supplementary_tables-R3_wrag230

ISME-Supplementary_methods-R3_wrag230

## Data Availability

The datasets generated during and analysed during the current study are available in the Edmond repository, https://doi.org/10.17617/3.OL50C0.
