# Supplementary material for: Fungi associated with Norway spruce and its bark beetles detoxify spruce defense compounds by conjugation to α-ribose
Source: ISME J. 2026 Sep 4;20(1):wrag230. doi: 10.1093/ismejo/wrag230 (PMC13637821; doi:10.1093/ismejo/wrag230)
Supplement: Supplementary file 1 [file isme-supplementary_figures-r2_wrag230.pdf]

Supplementary Figures

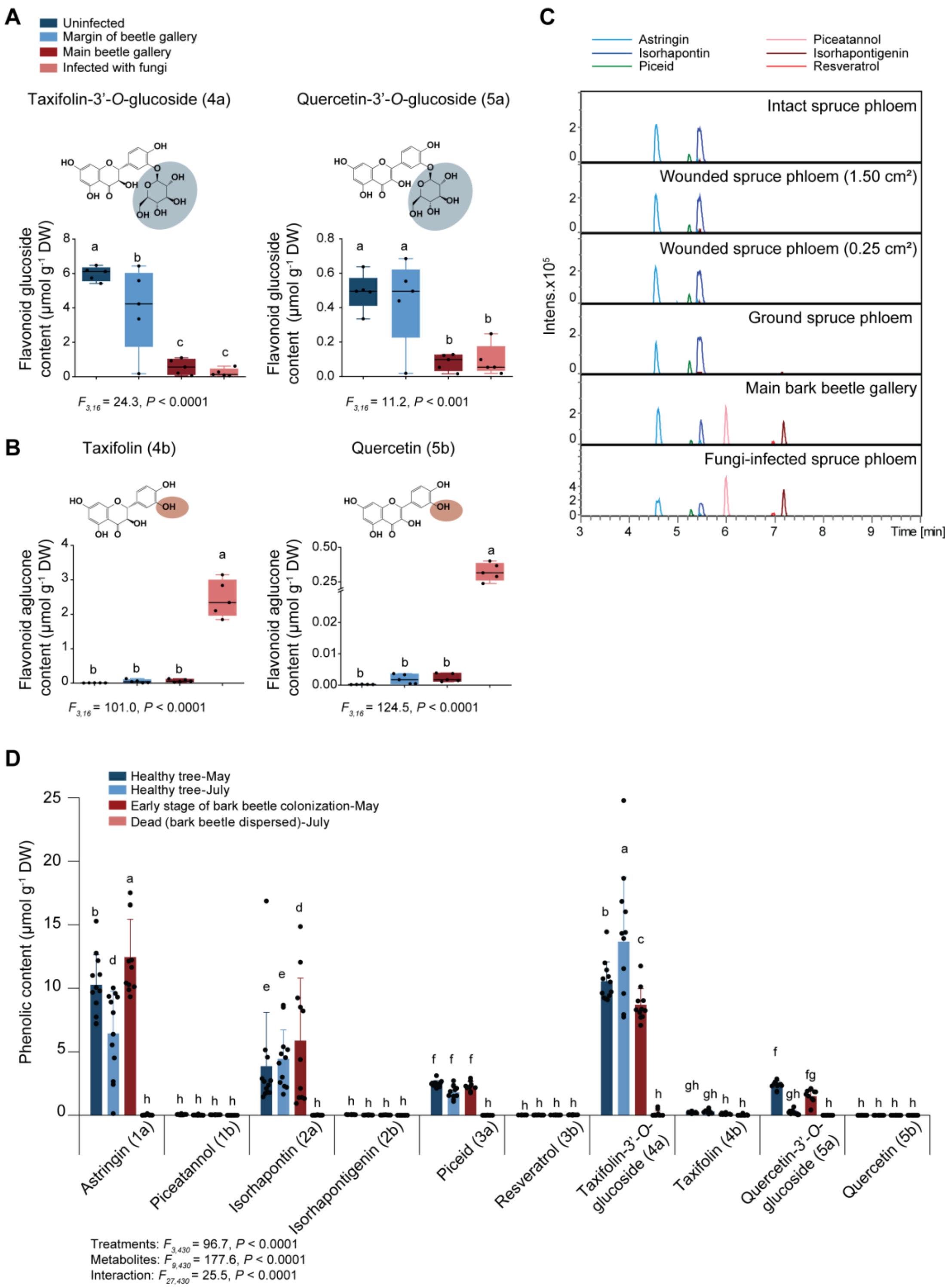

Figure S1. Degradation of glucosides in the phloem of Norway spruce following fungal colonization and bark beetle infestation. Quantification of flavonoid glucosides (**A**) taxifolin-3′-*O*-glucoside and quercetin-3′-*O*-glucoside, and flavonoid aglucones (**B**) taxifolin and quercetin in uninfected phloem, fungal-infected phloem, margin of beetle gallery, and main beetle gallery (*n* = 5). In the chemical structures, glucose and aglucone moieties are highlighted with light blue and red ovals, respectively. Box plots show the interquartile range of 25 to 75%,, with centre lines indicating median values and whiskers extending to 1 time the interquartile range. (**C**) Stilbene aglucones were formed by biotransformation of stilbene glucosides in fungi-infected phloem and bark beetle galleries, but not in mechanically wounded phloem. Extracted ion chromatograms are from spectra obtained from UHPLC-qTOF-MS in the negative ionization mode. Representative mass spectra of phenolic compounds and authentic standards are shown in Supplementary File 2. **(D)** Phenolic content in the bark of healthy and bark beetle-infested Norway spruce trees, collected in May and July from Kranichfeld (Thuringia, Germany) (*n* = 11-12). Statistical significance among groups was assessed using one-way ANOVA in **A-B** and two-way ANOVA in **D**, followed by Fisher's LSD tests ($P < 0.05$). Different lowercase letters indicate significant differences.

^{1}H NMR with water suppression, full range in MeOH-d_3

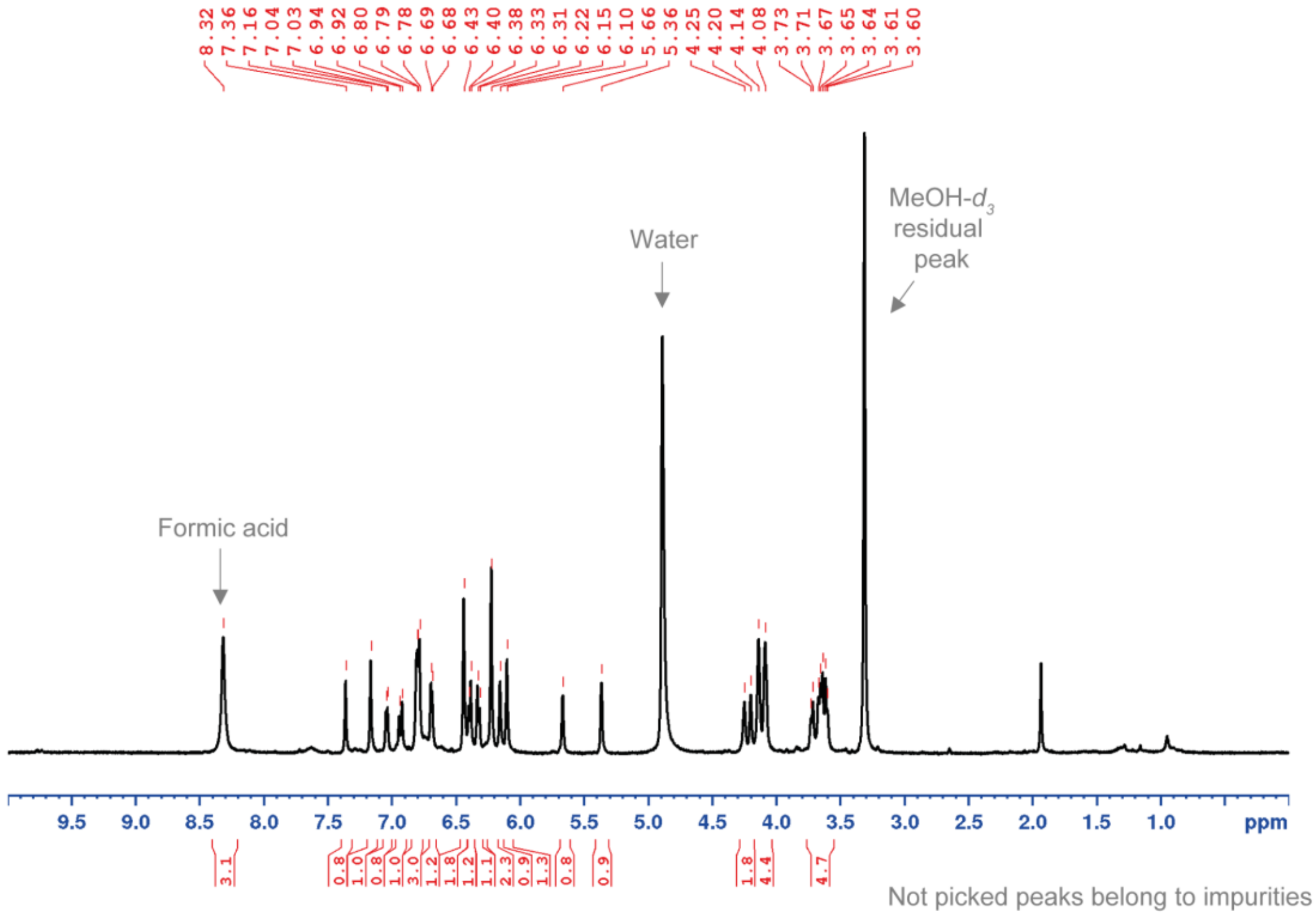

^{1}H NMR with water suppression, 6.0-7.5 ppm(above) , 3.0-4.5 ppm (below) in MeOH-d_3

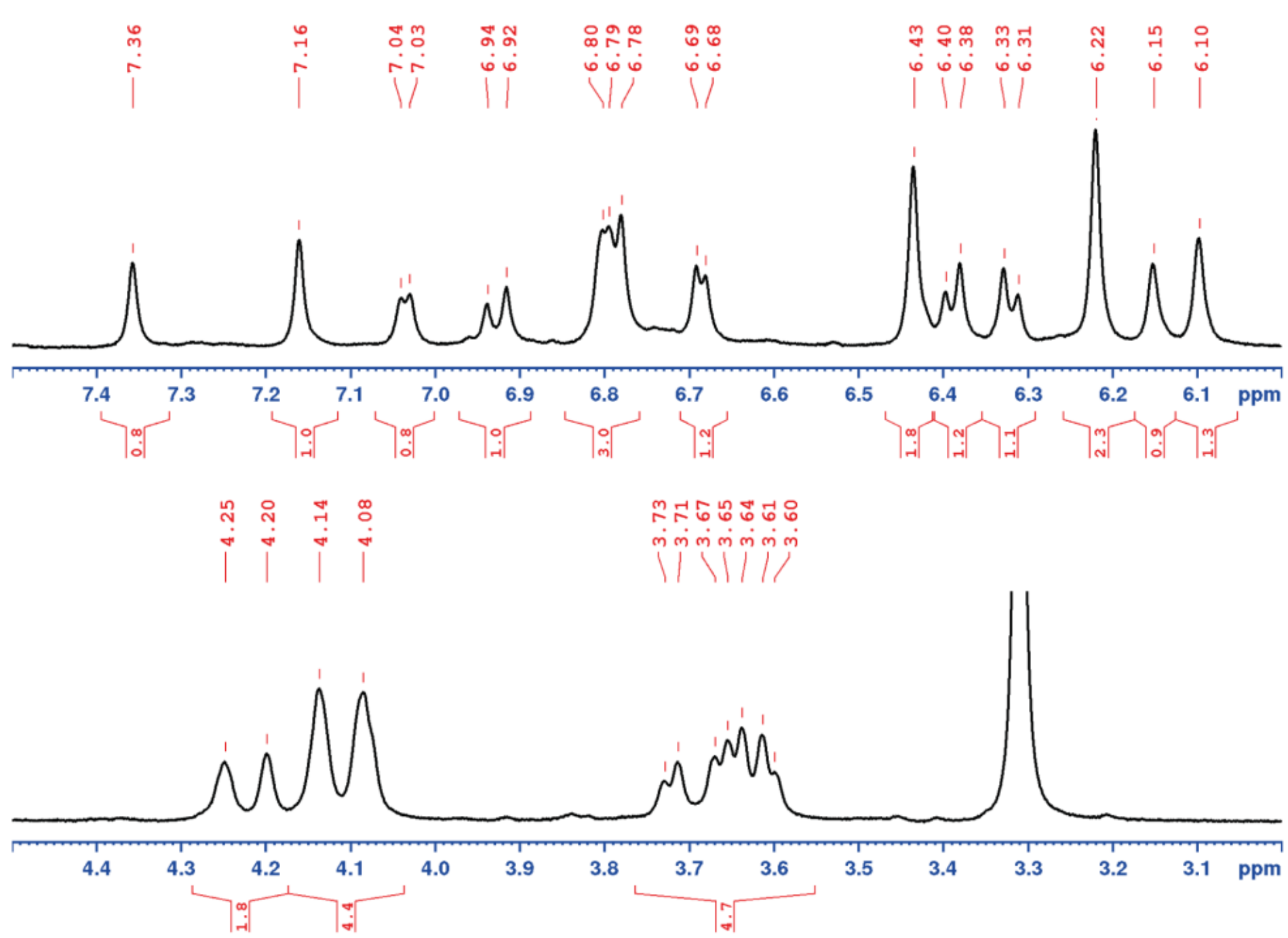

DEPTQ, full range in MeOH-d_3

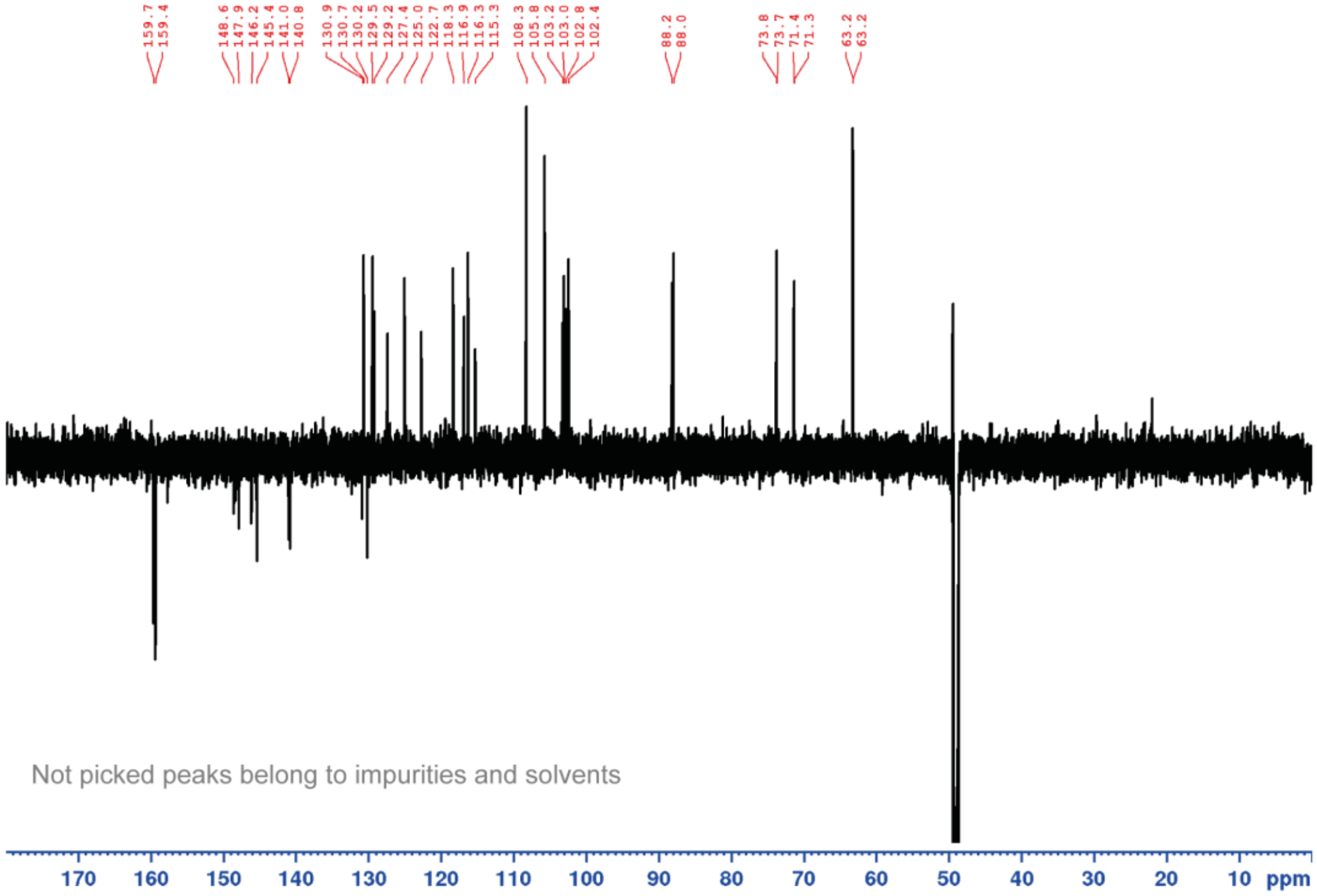

DEPTQ, 60-165 ppm in MeOH-d_3

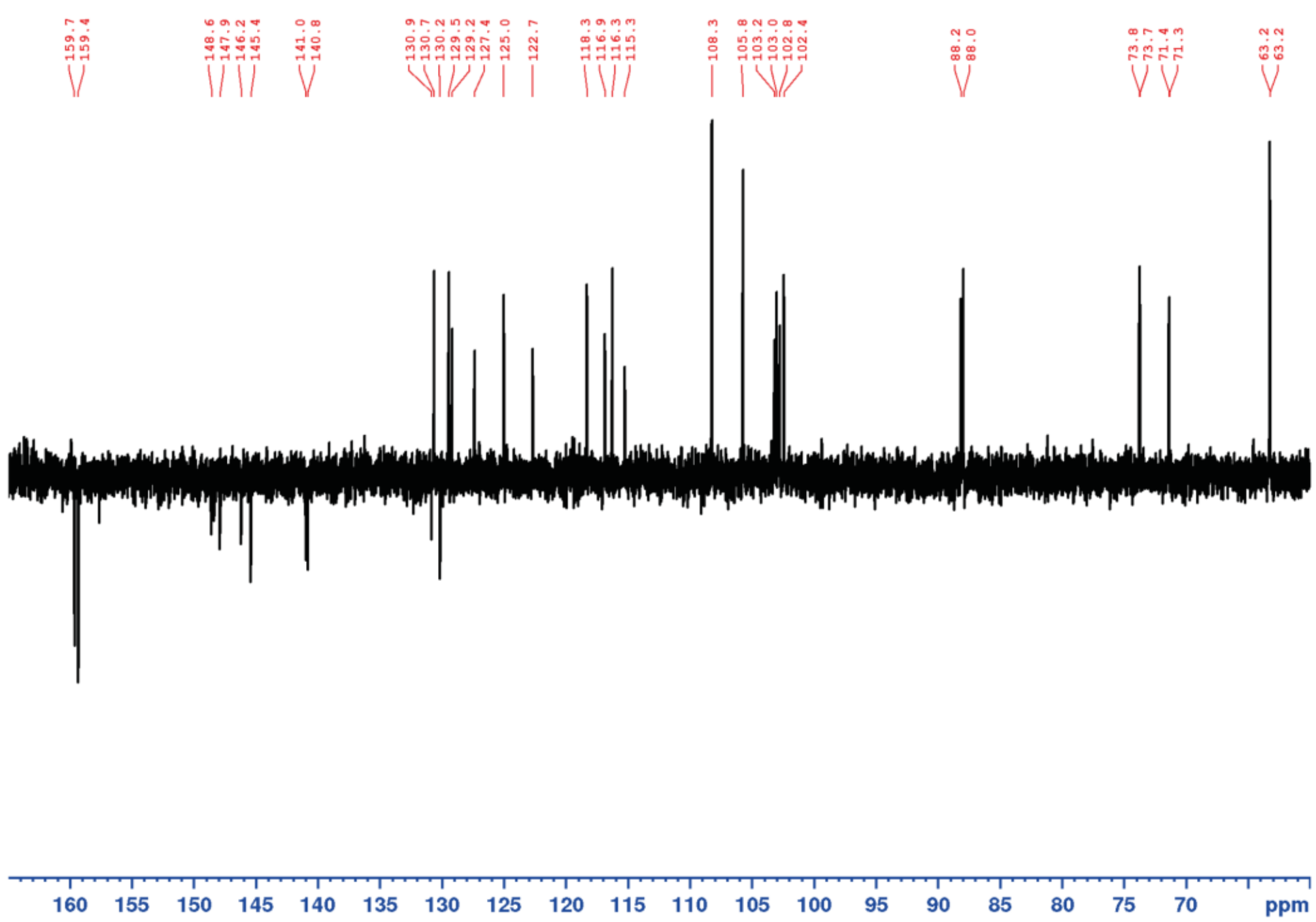

20

COSY with water suppression, 3.0-8.0 ppm in MeOH-d_3

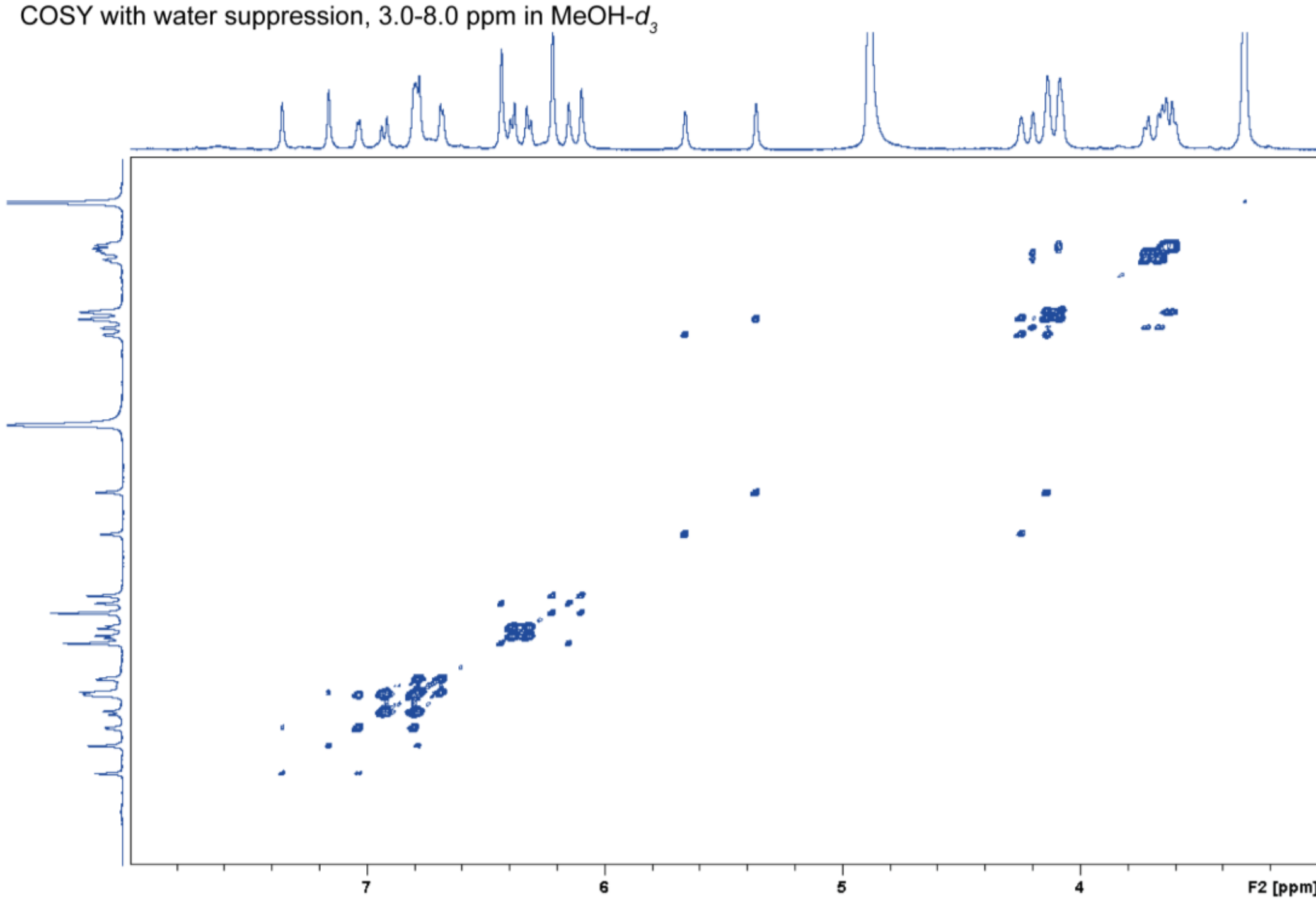

Phase sensitive HSQC (blue: CH, CH_3, green: CH_2)/ HMBC (red), 40-170 ppm in MeOH-d_3

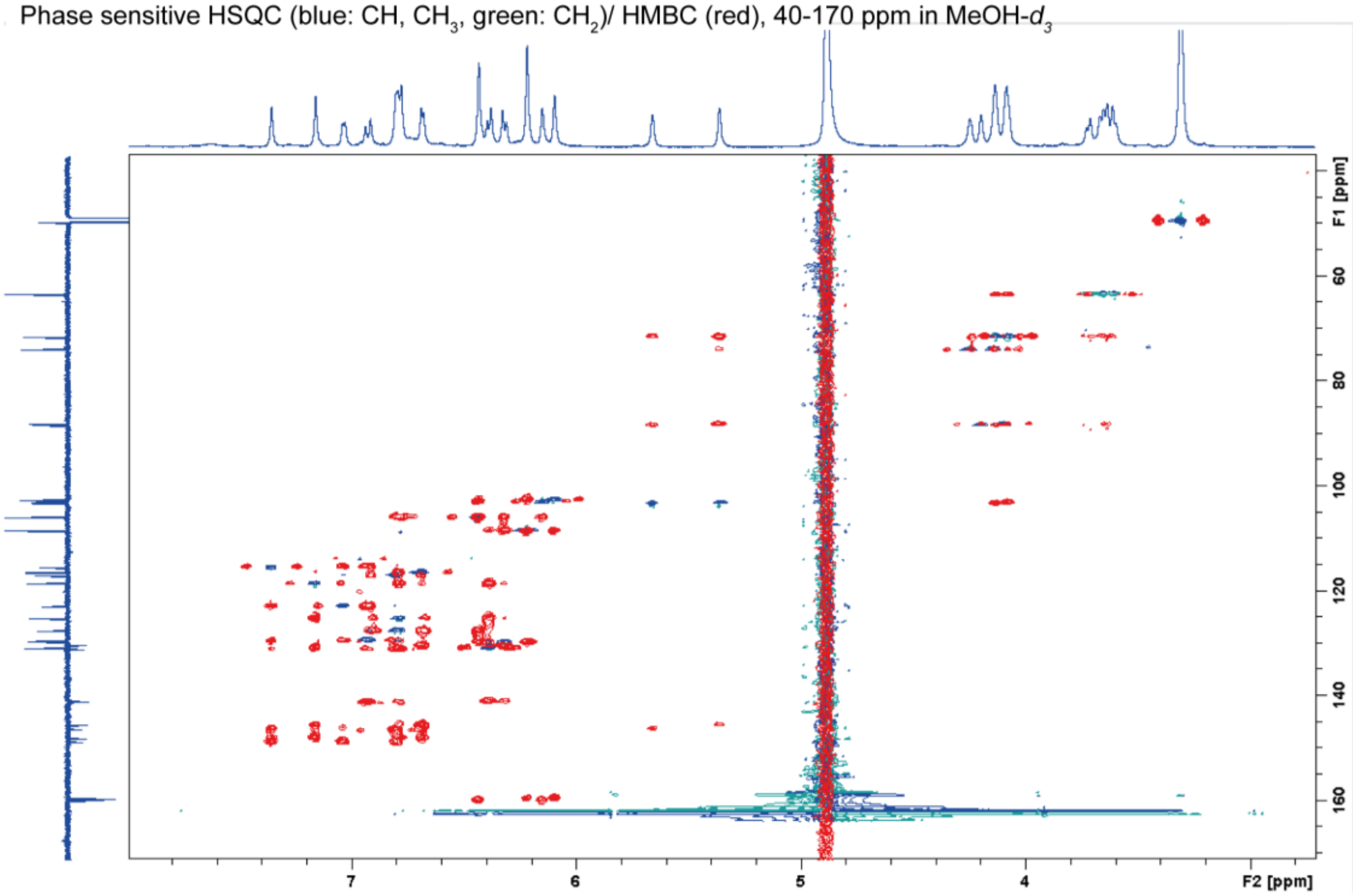

Figure S2A NMR spectra for piceatannol-11-*O*-α-ribofuranoside (***E*-1c** and ***Z*-1c**, ***E*** : ***Z*** = 1 : 1.1)

^{1}H NMR with water suppression, full range in MeOH-d_3

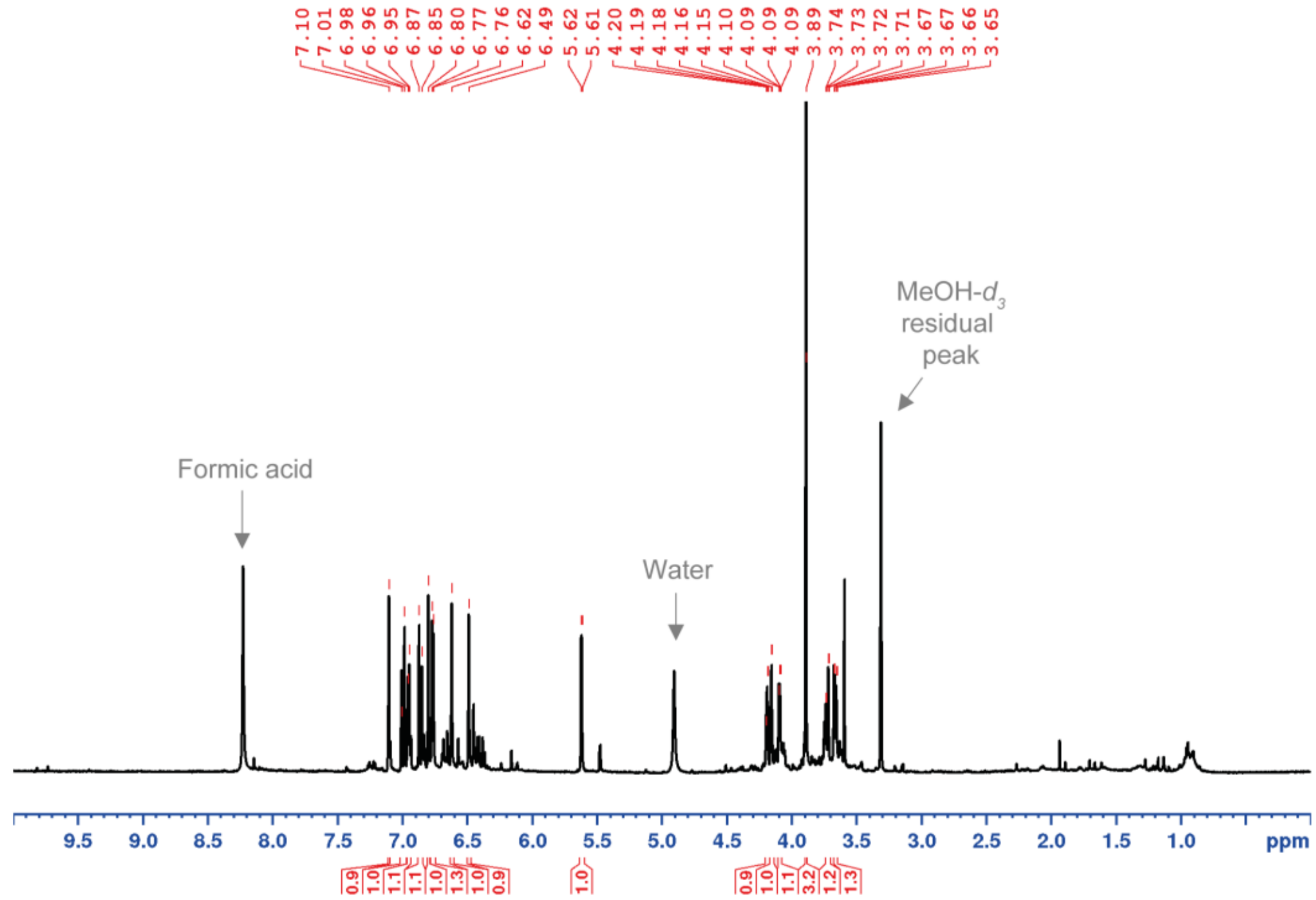

Not picked peaks belong to impurities, solvents, *Z*-isomer

^{1}H NMR with water suppression, 6.3-7.3 ppm(above) , 3.5-4.3 ppm (below) in MeOH-d_3

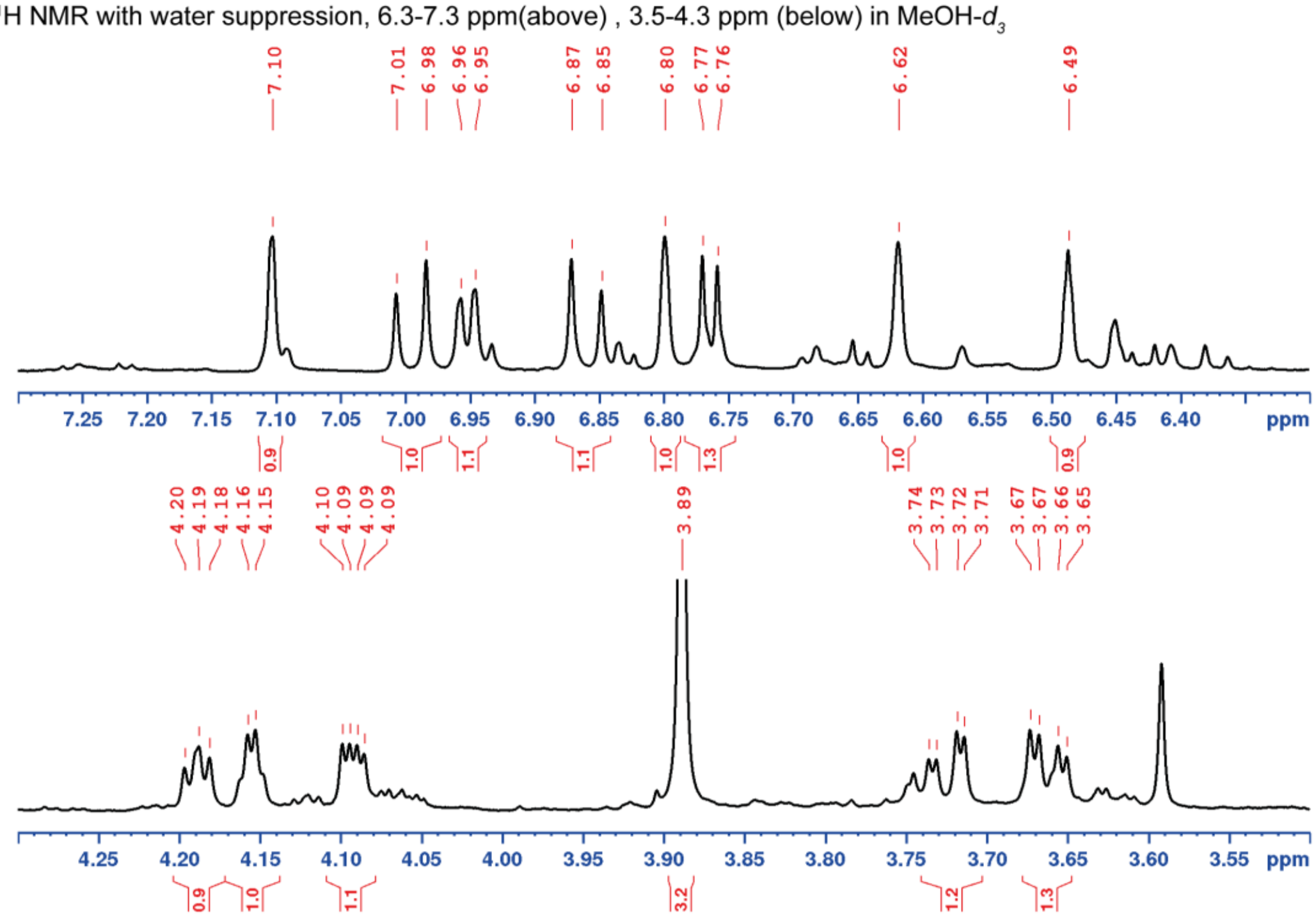

24

DEPTQ, full range in MeOH-d_3

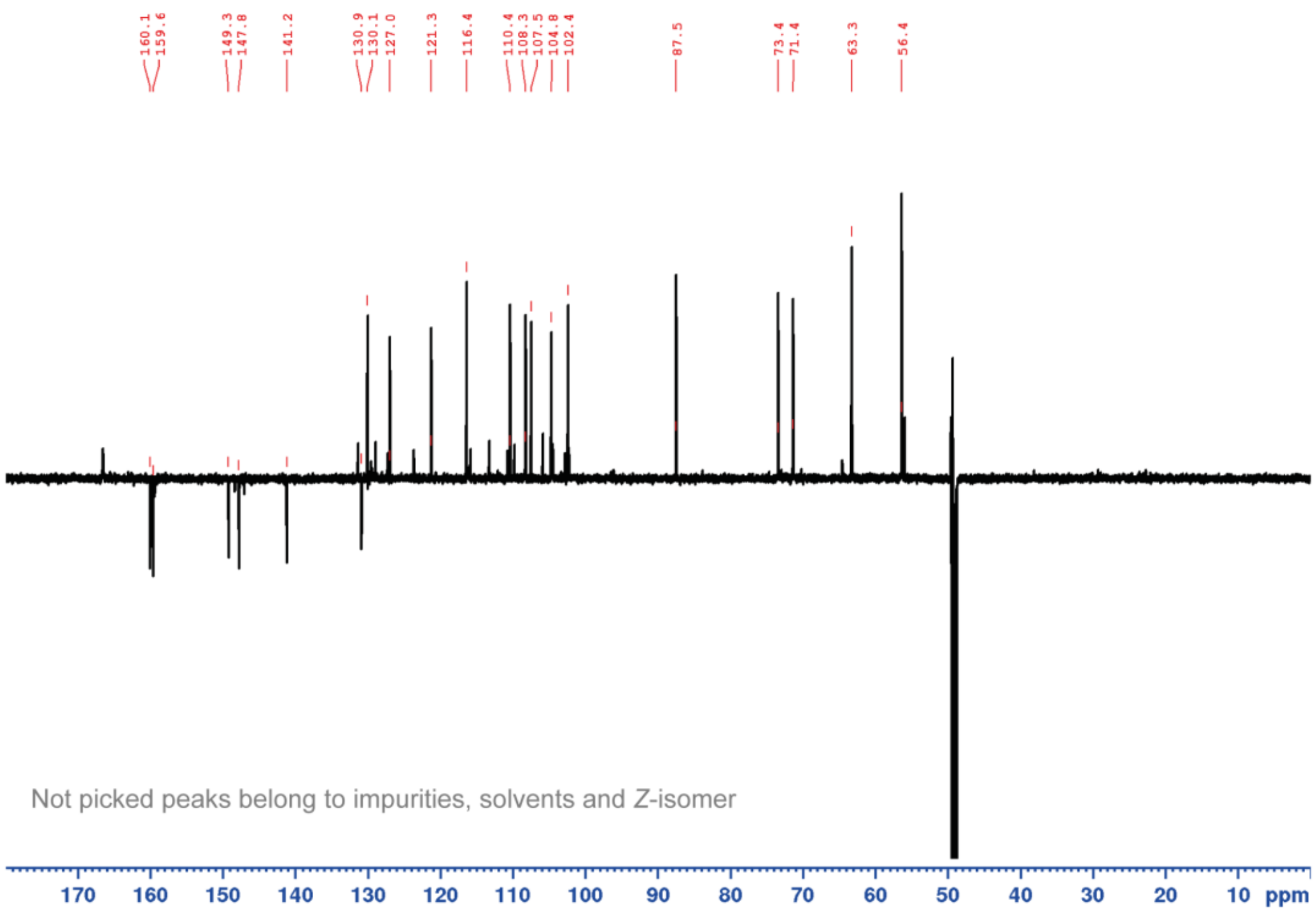

DEPTQ, 60-165 ppm in MeOH-d_3

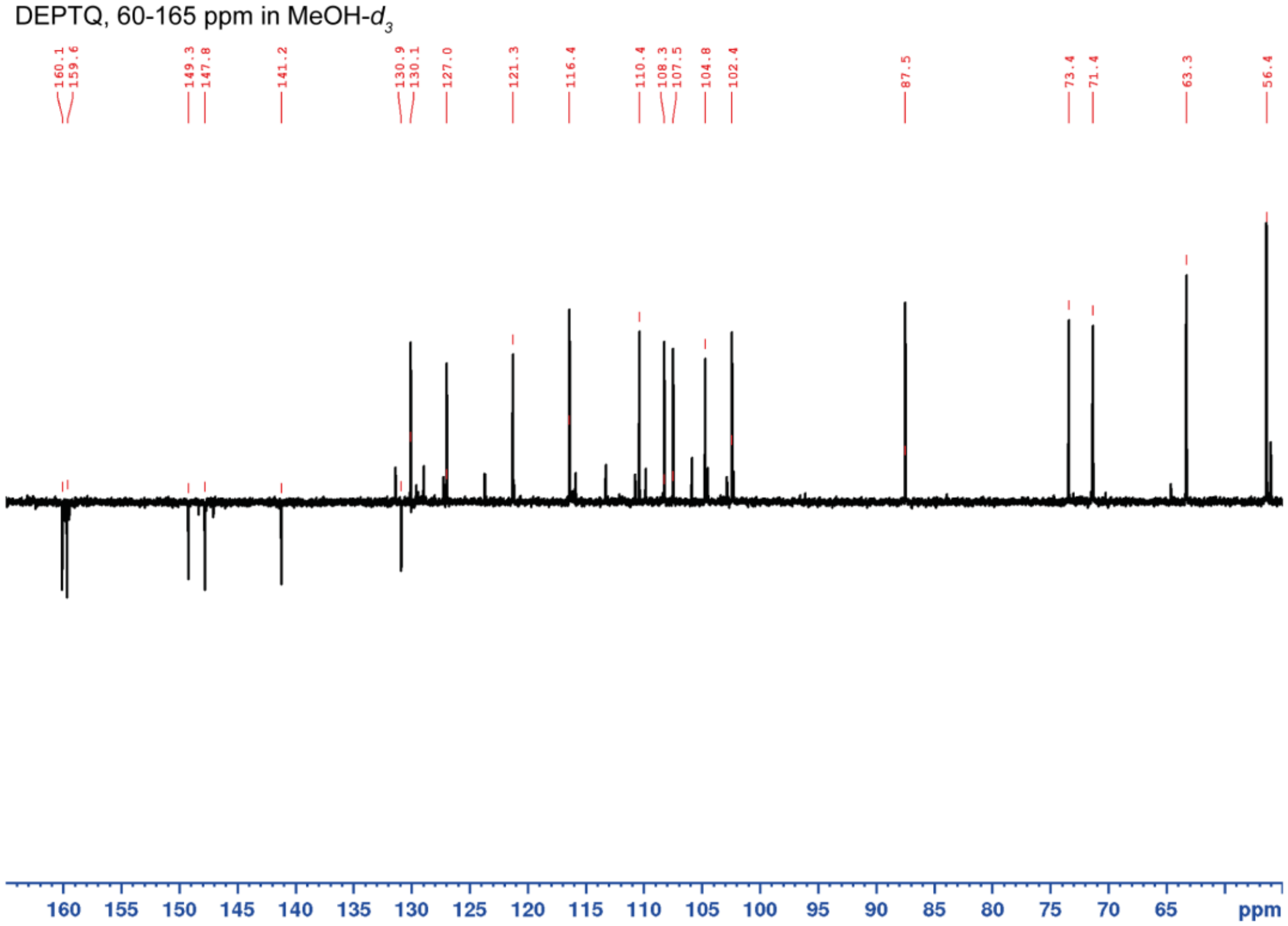

25

COSY with water suppression, 3.0-7.4 ppm in MeOH-d_3

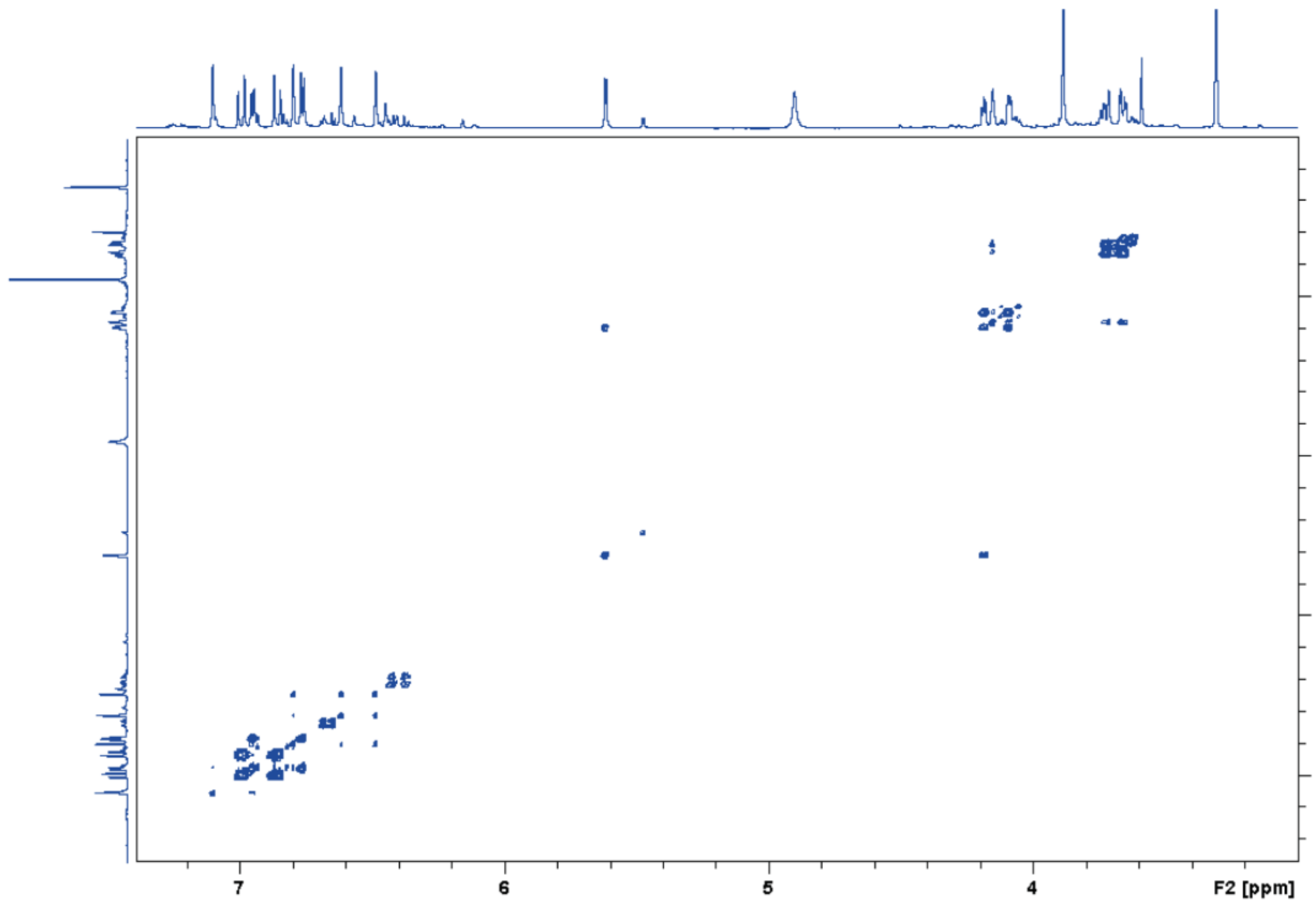

Phase sensitive HSQC (blue: CH, CH_3, green: CH_2)/ HMBC (red), 45-170 ppm in MeOH-d_3

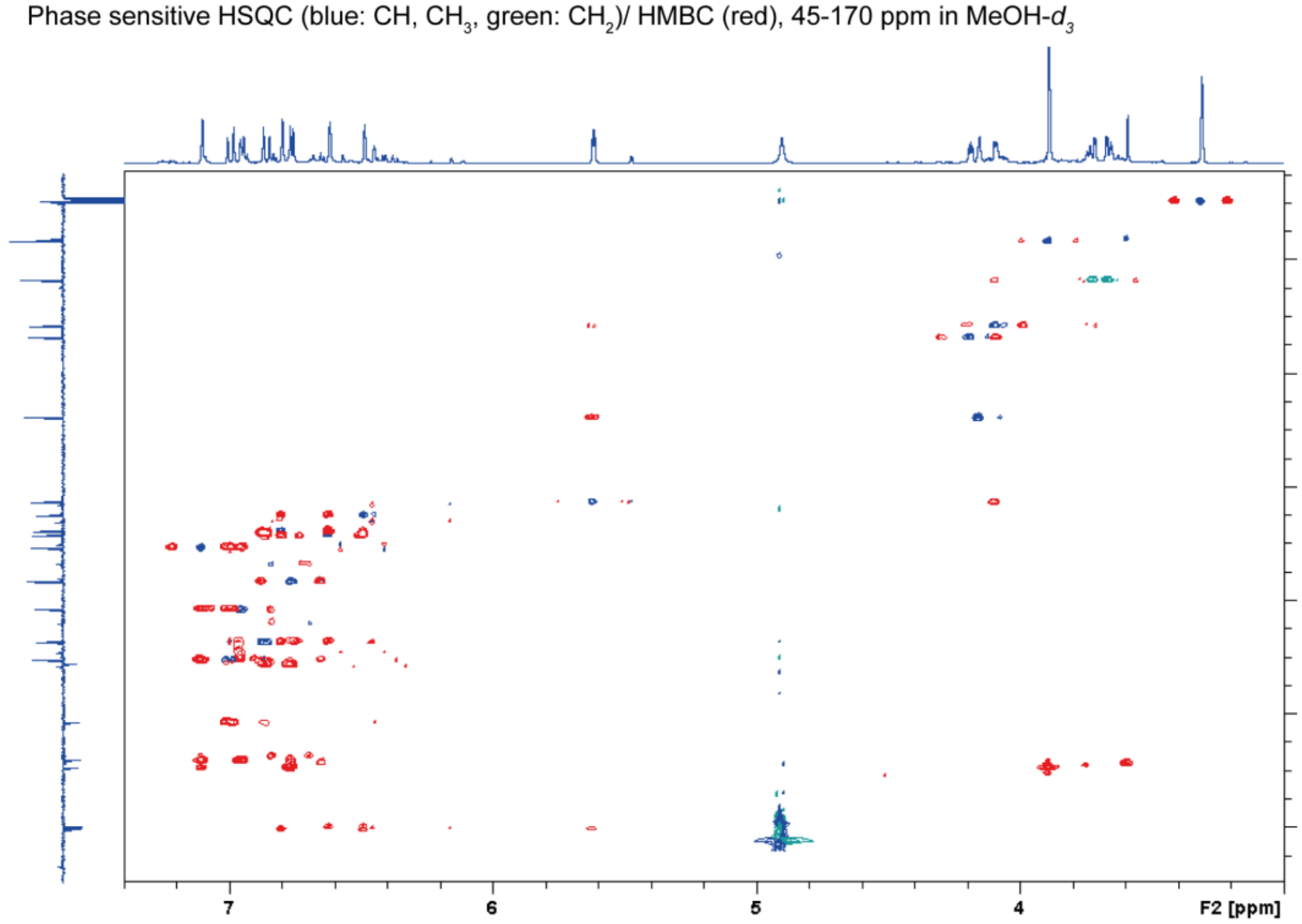

Figure S2B NMR spectra for isorhapontigenin-3-*O*-α-ribofuranoside (***E*-2c**, ***E*** : ***Z*** = 1 : 0.25)

^{1}H NMR, full range in DMSO-d_6

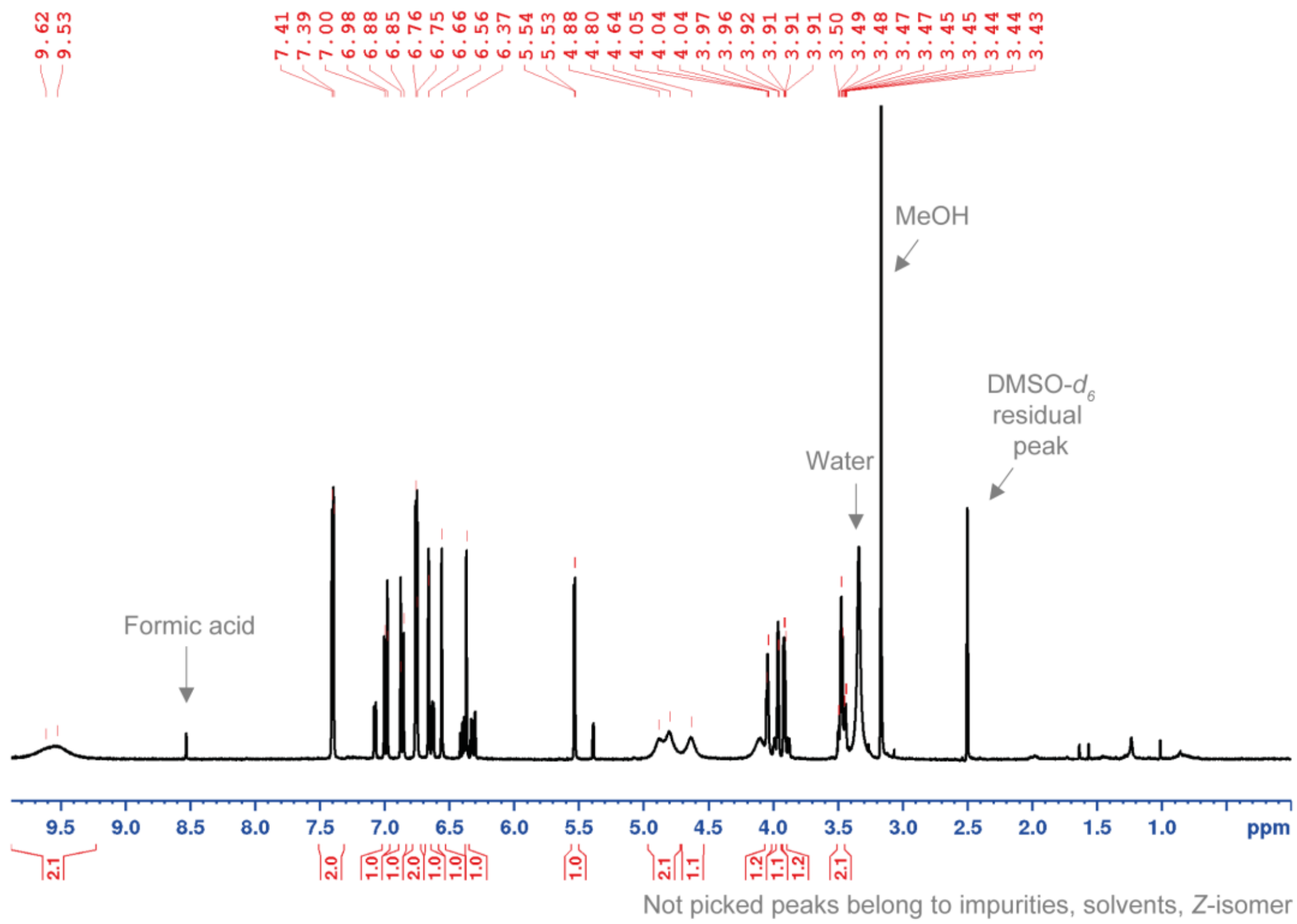

^{1}H NMR, 6.2-7.5 ppm(above) , 3.4-5.7 ppm (below) in DMSO-d_6

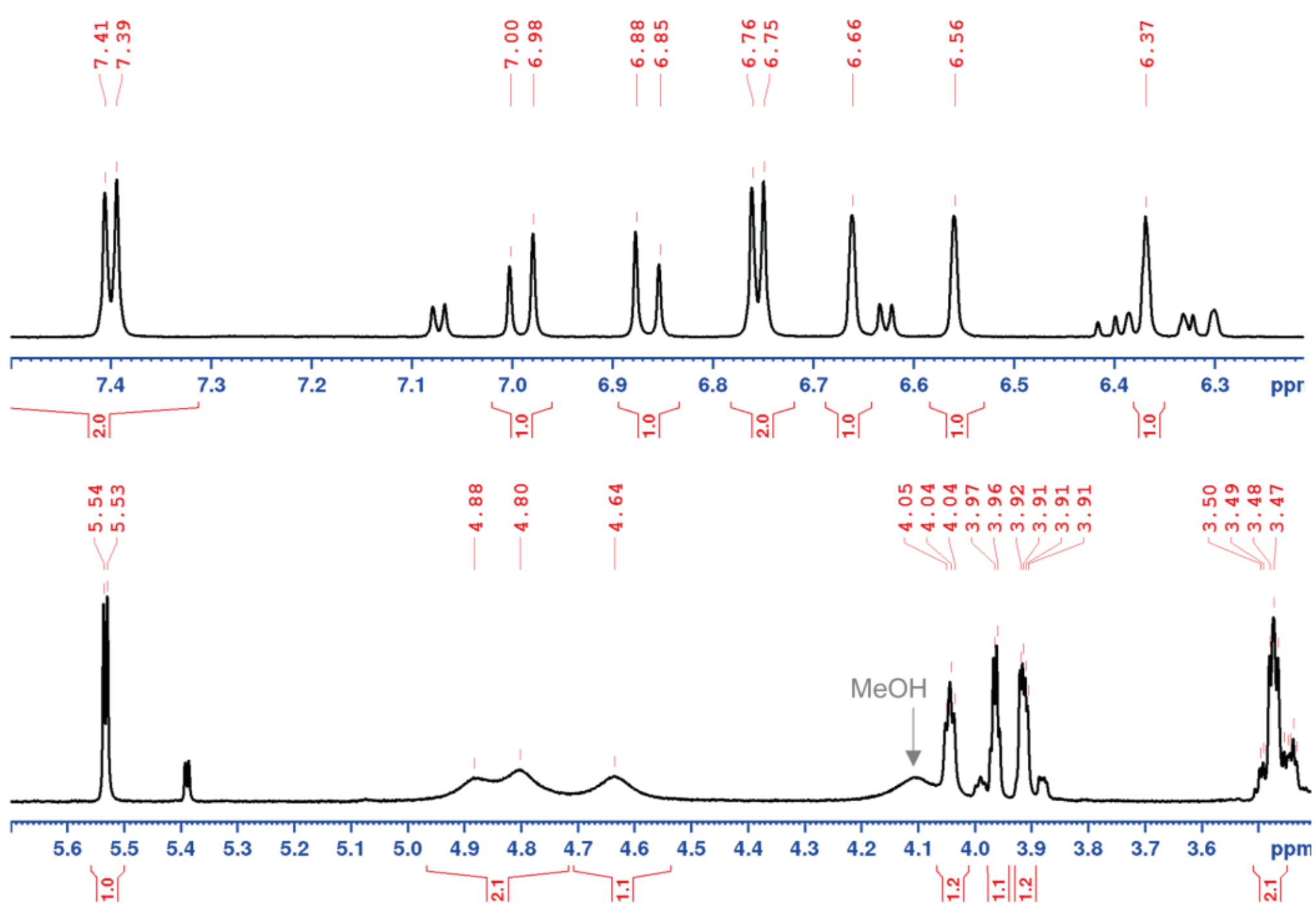

28

DEPTQ, full range in DMSO-d_6

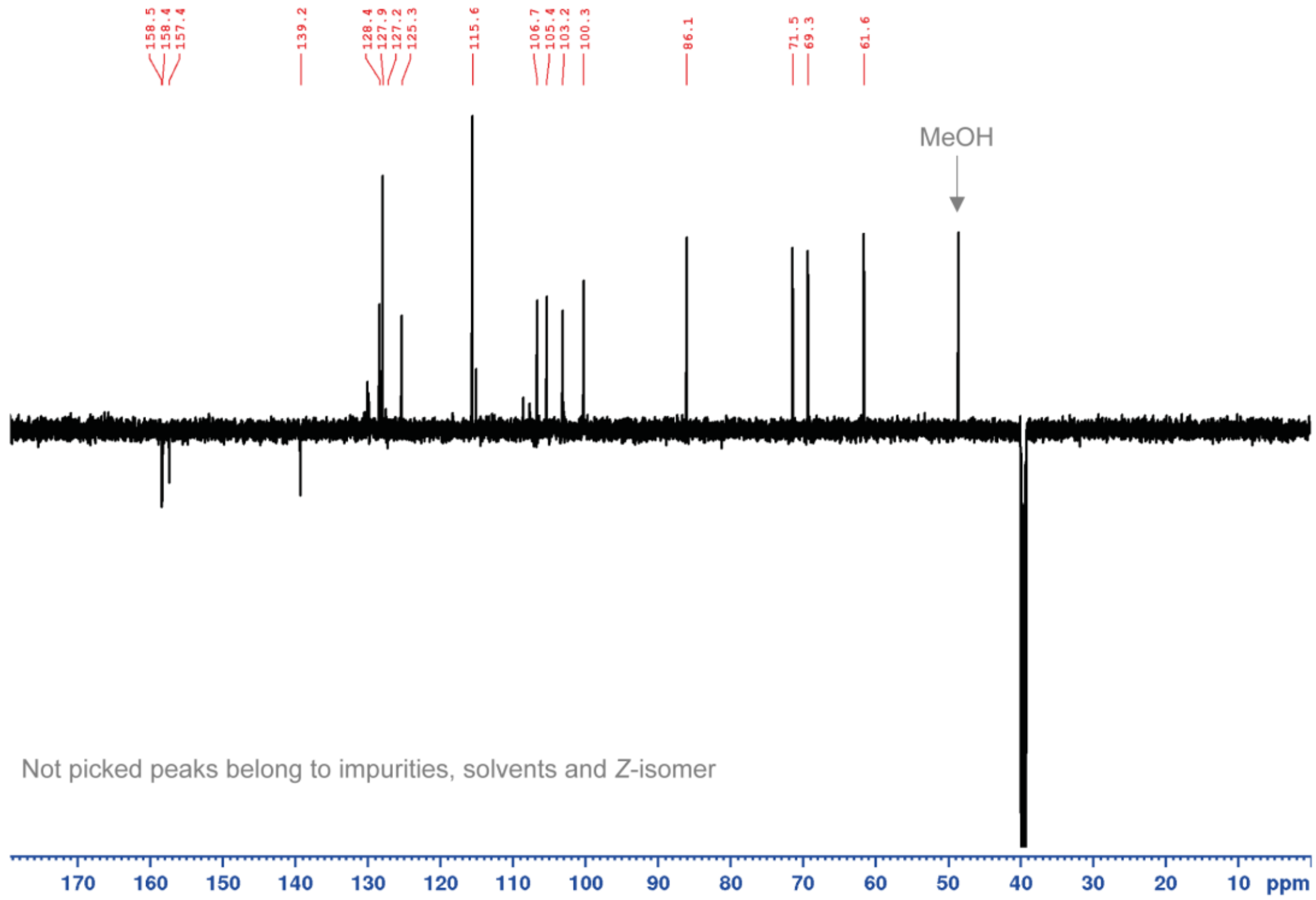

DEPTQ, 60-165 ppm in DMSO-d_6

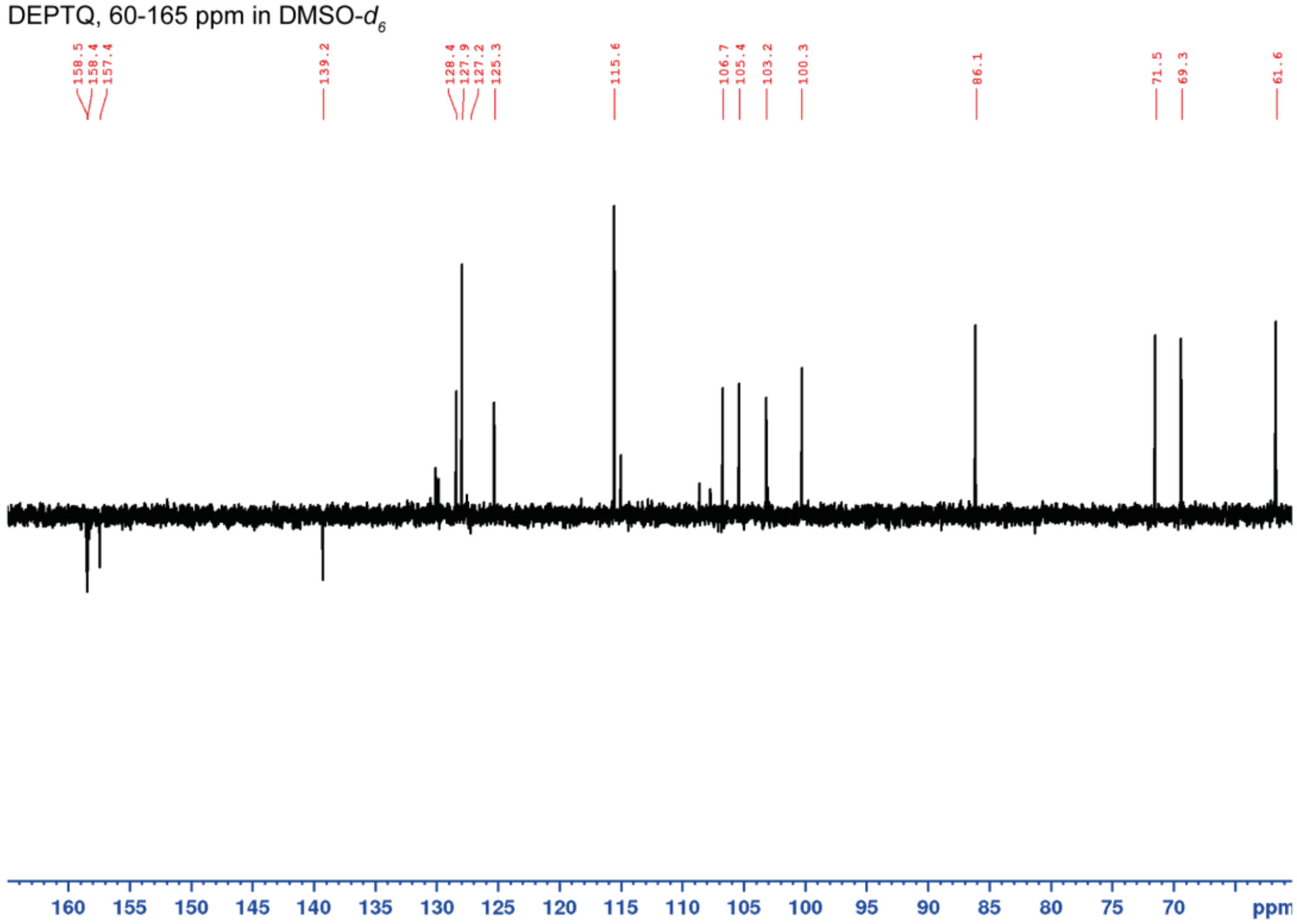

29

COSY with water suppression, 3.0-8.0 ppm in DMSO-d_6

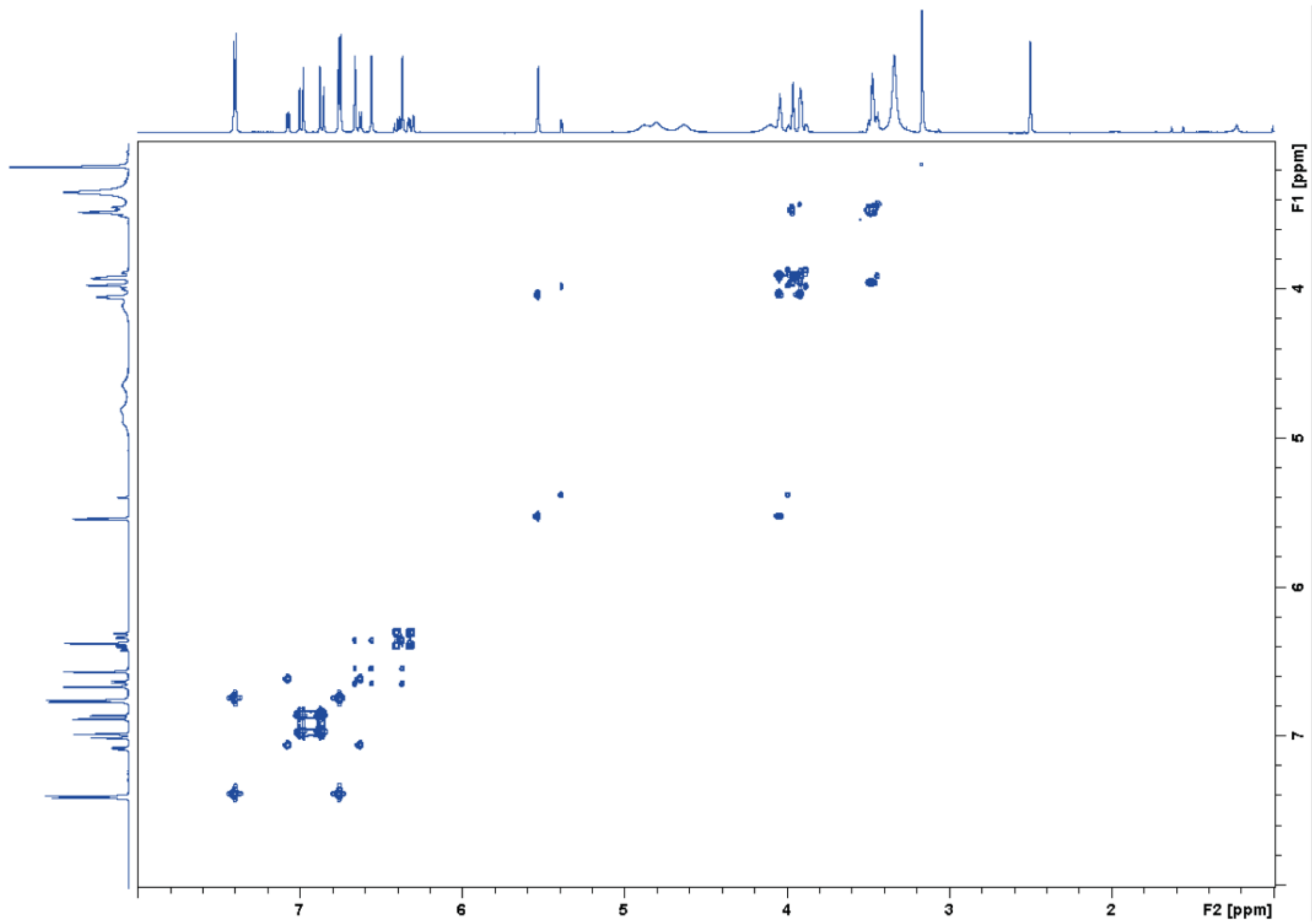

Phase sensitive HSQC (blue: CH, CH_3, green: CH_2)/ HMBC (red), 45-170 ppm in DMSO-d_6

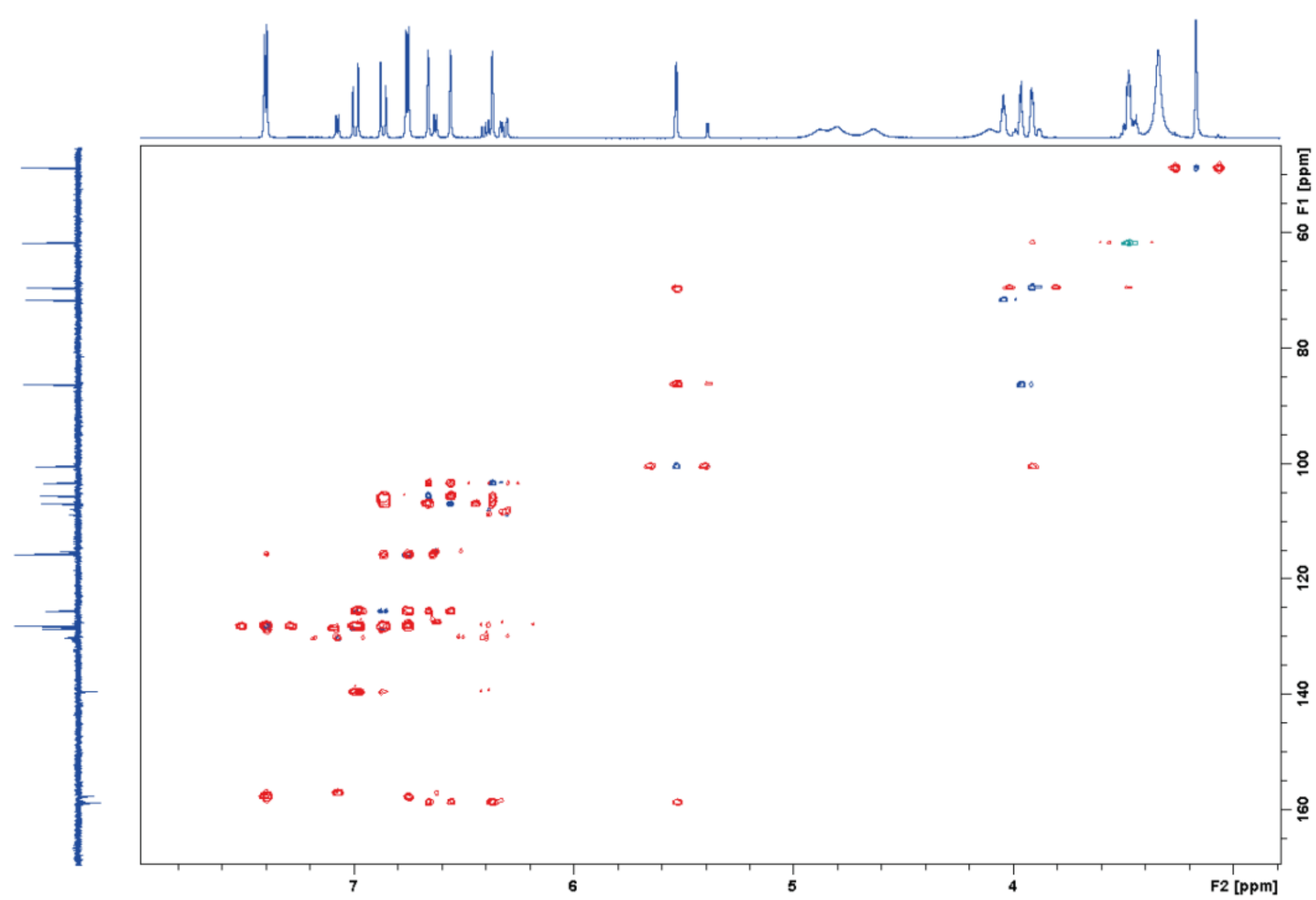

Figure S2C NMR spectra for resveratrol-3-*O*-α-ribofuranoside (***E*-3c**, ***E* : *Z*** = 1 : 0.19)

^{1}H NMR with water suppression, full range in MeOH-d_3

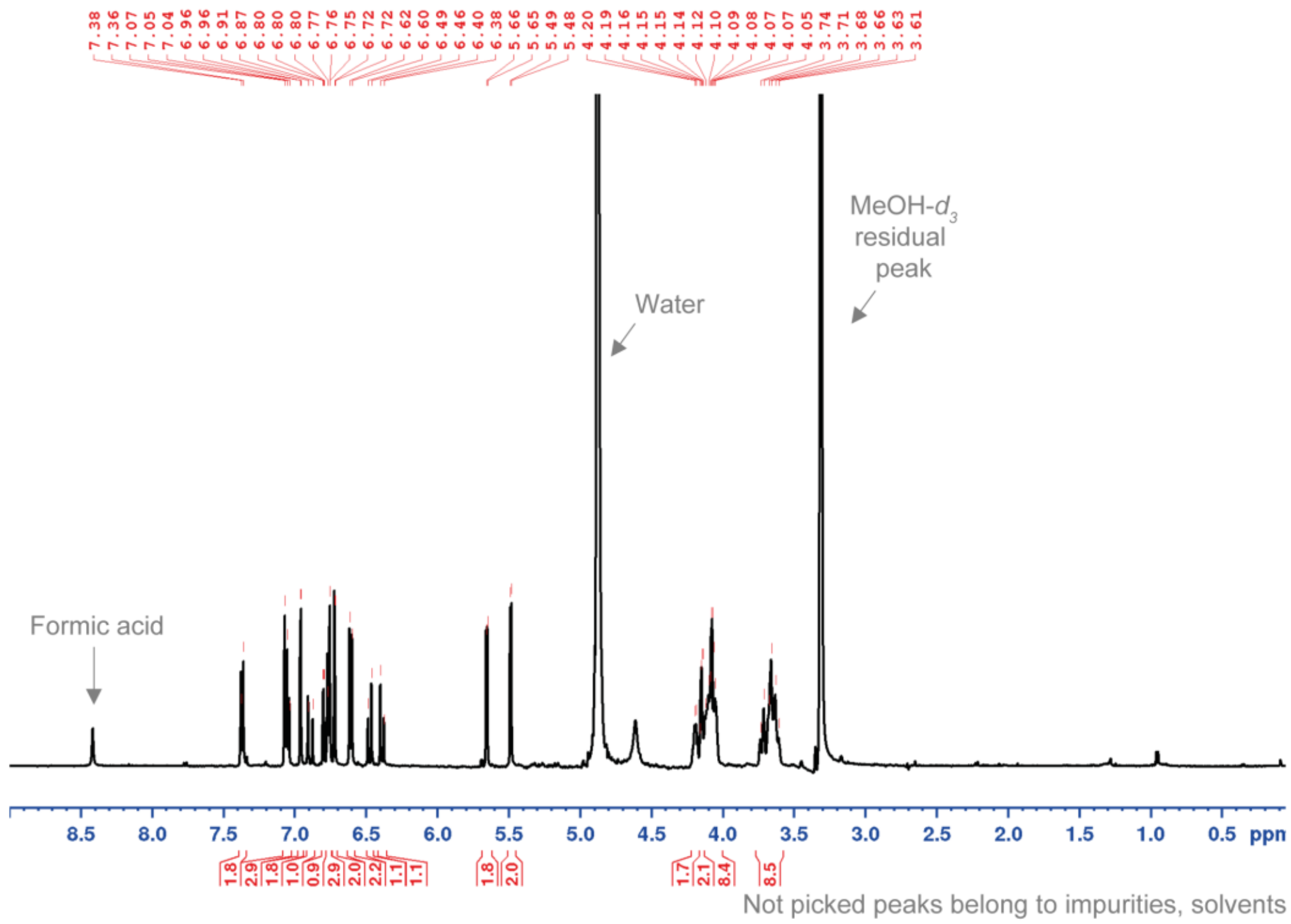

^{1}H NMR with water suppression, 5.3-7.5 ppm(above) , 3.4-4.5 ppm (below) in MeOH-d_3

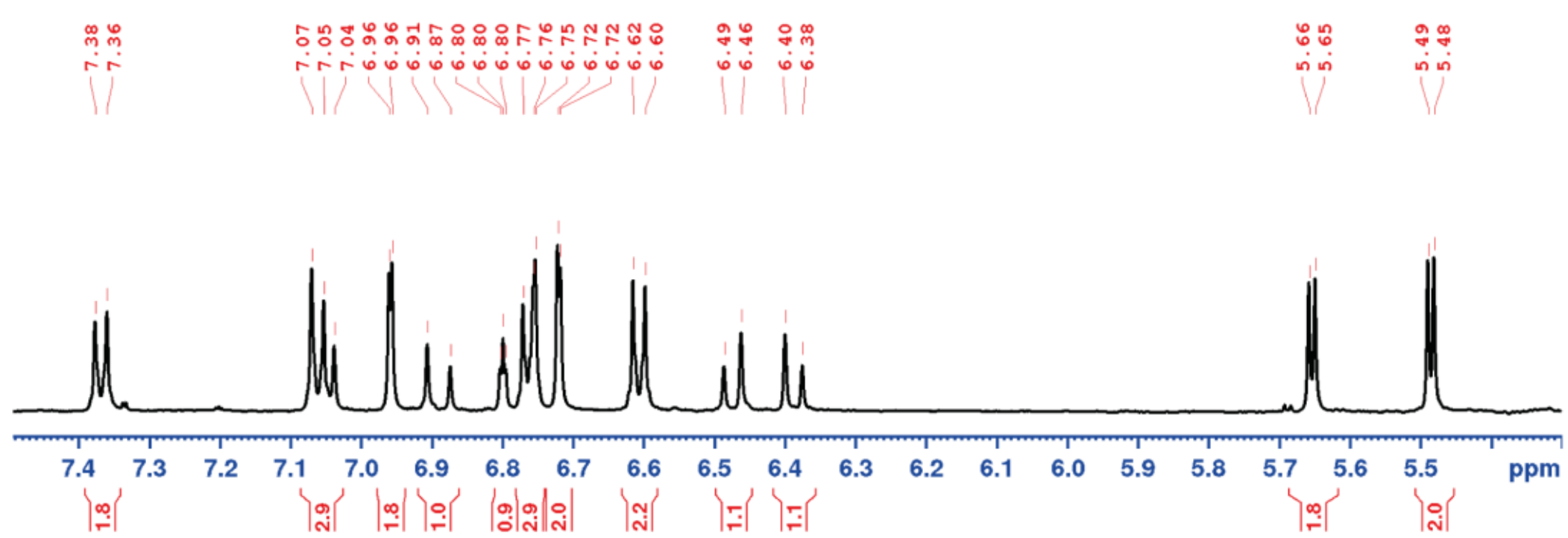

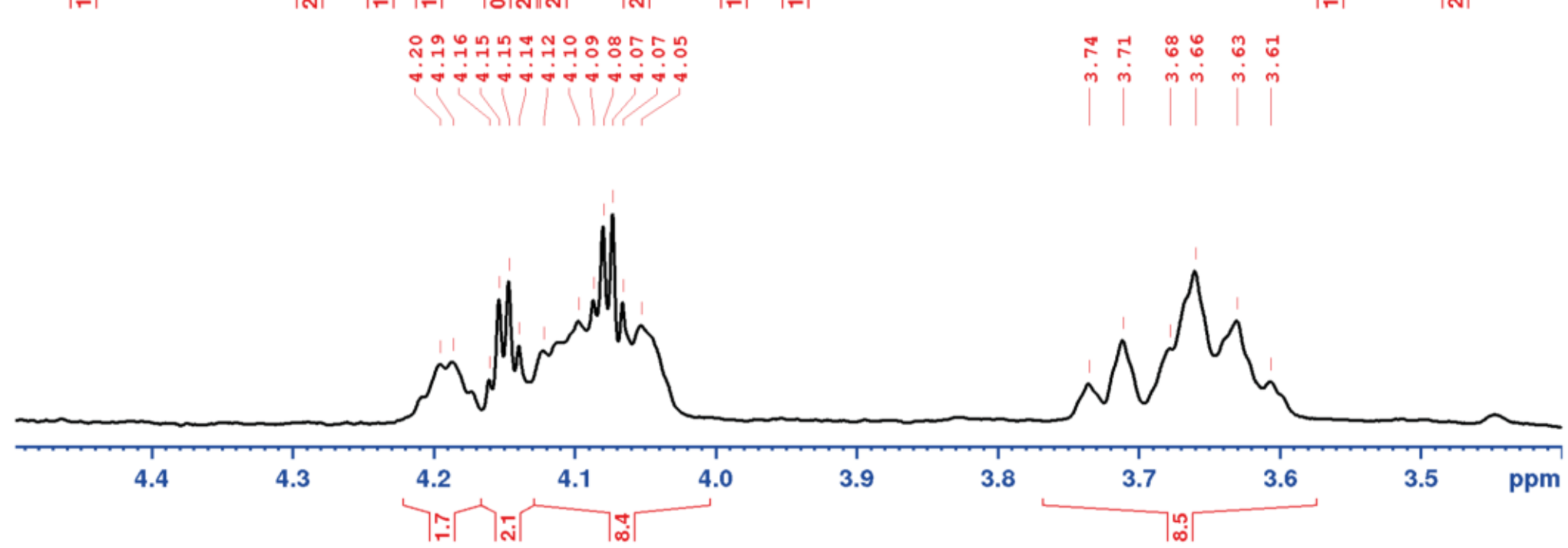

32

DEPTQ, full range in MeOH-d_3

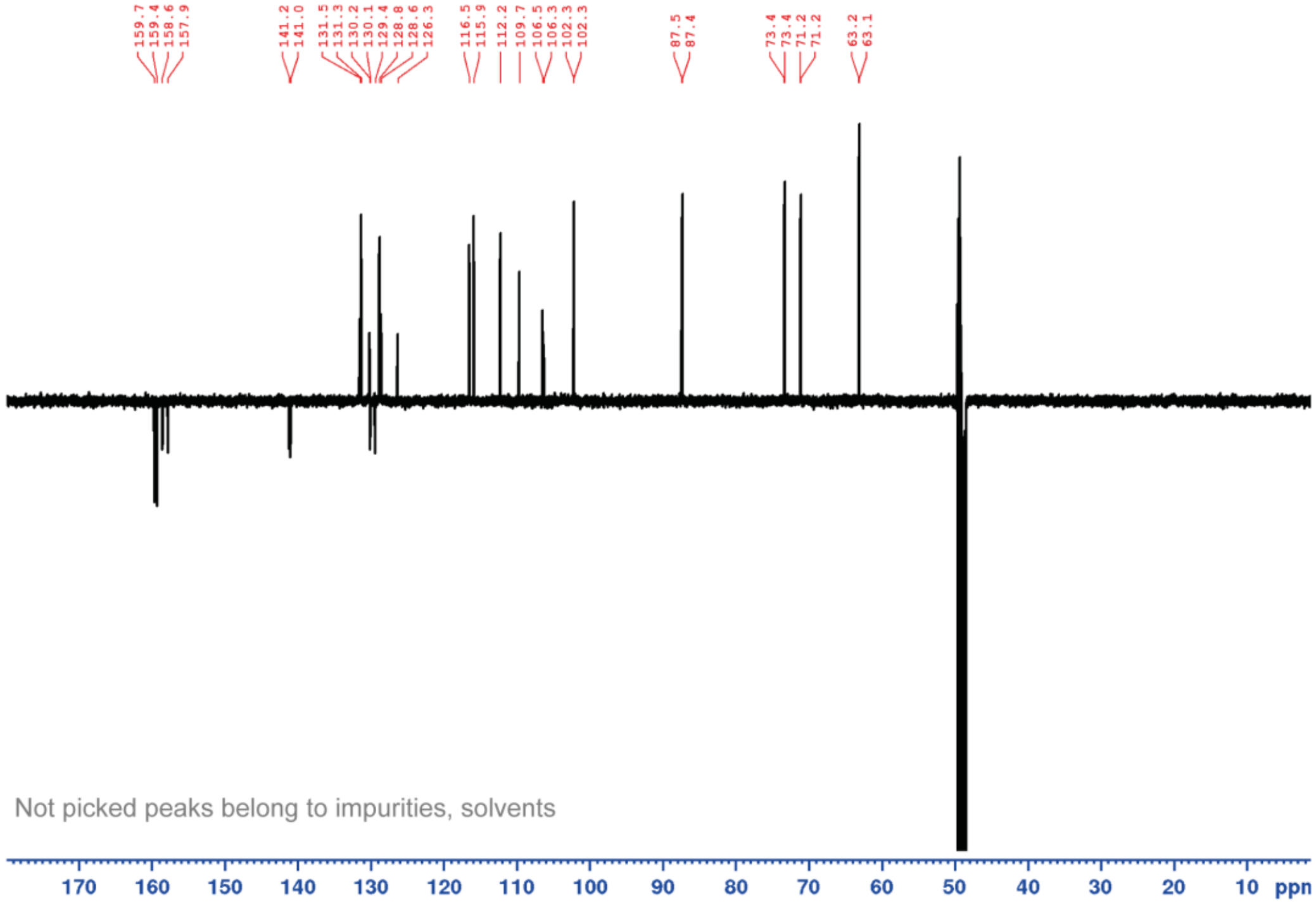

DEPTQ, 60-165 ppm in MeOH-d_3

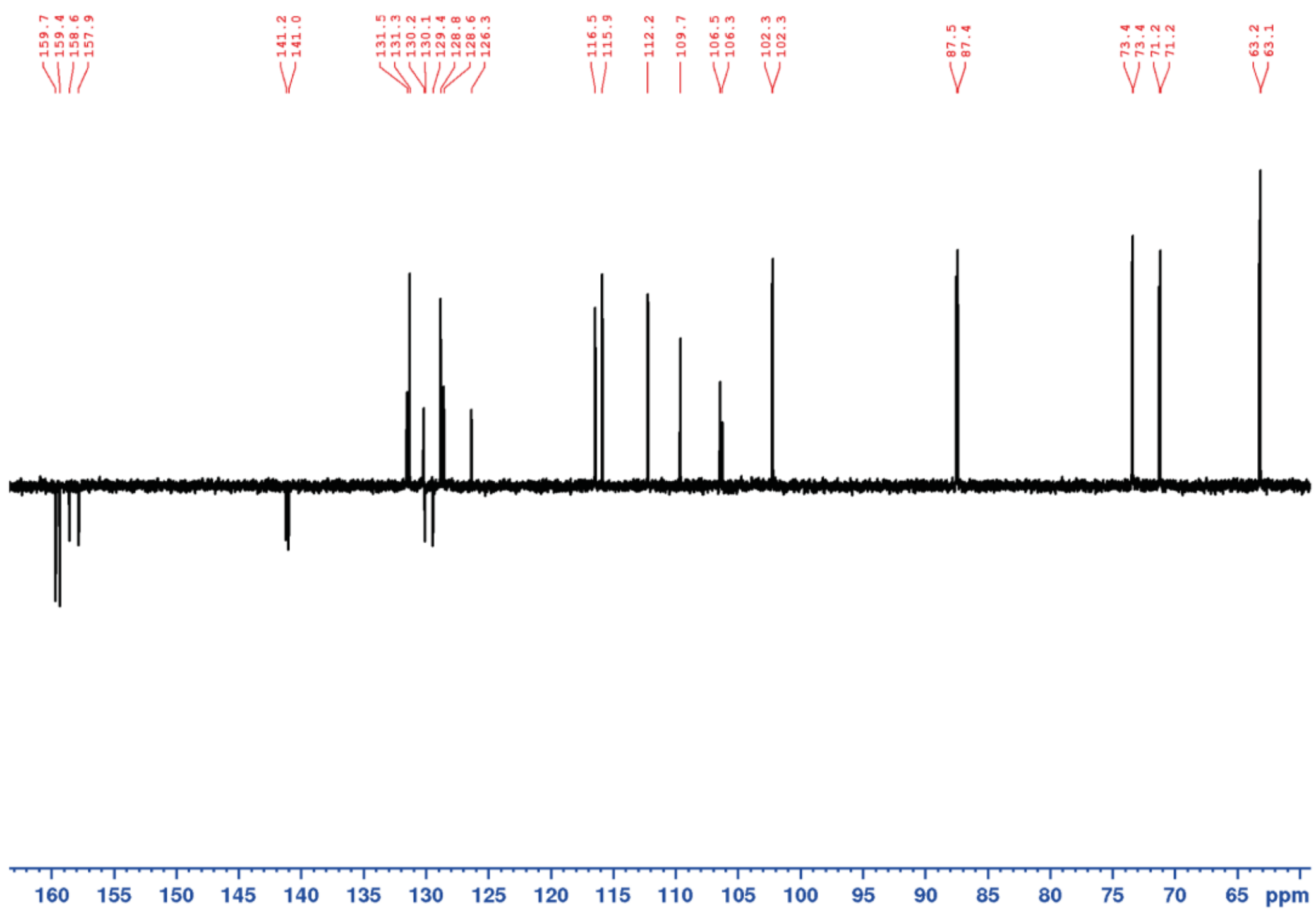

33

COSY with water suppression, 3.0-8.0 ppm in MeOH-d_3

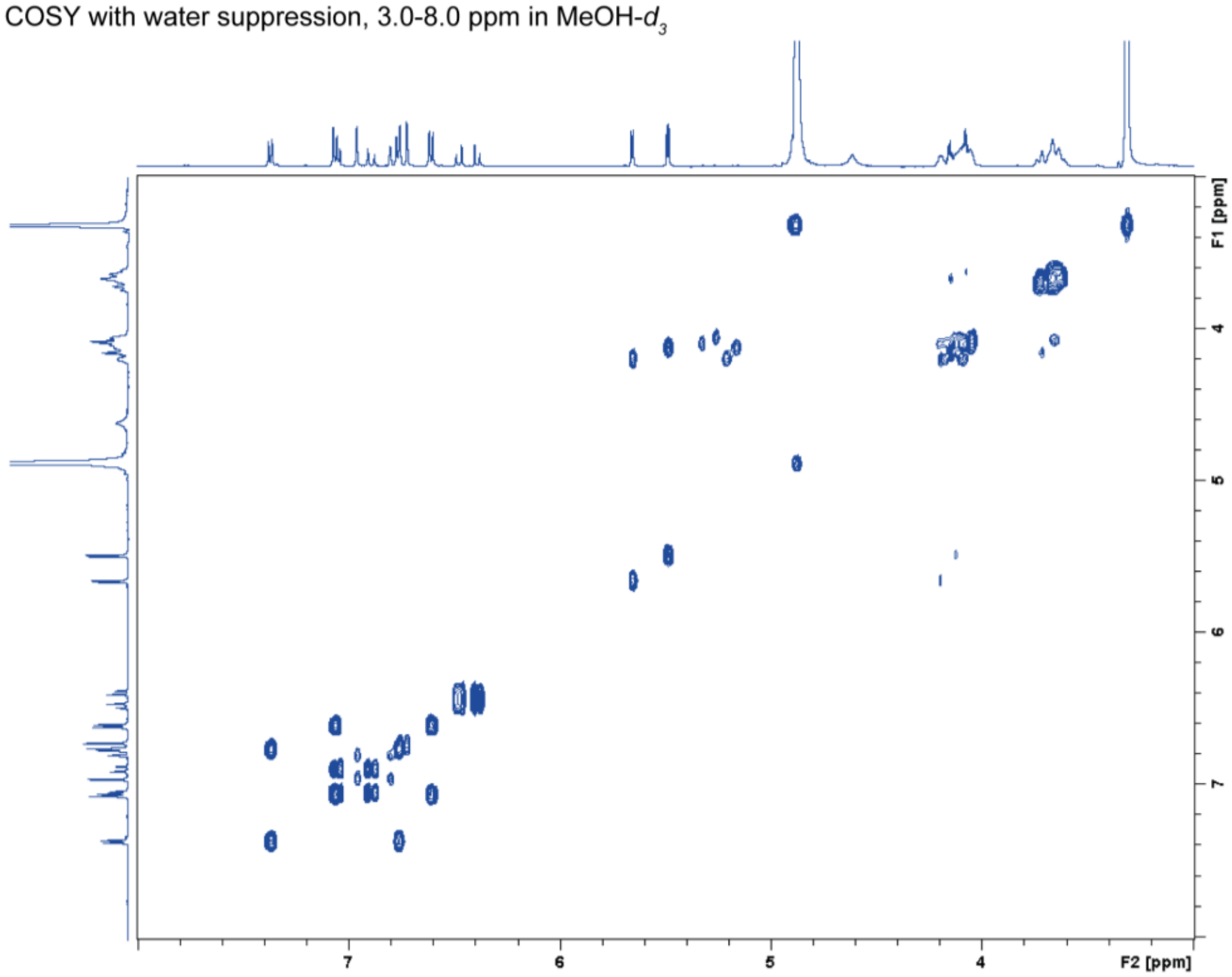

Phase sensitive HSQC (blue: CH, CH_3, green: CH_2)/ HMBC (red), 45-170 ppm in MeOH-d_3

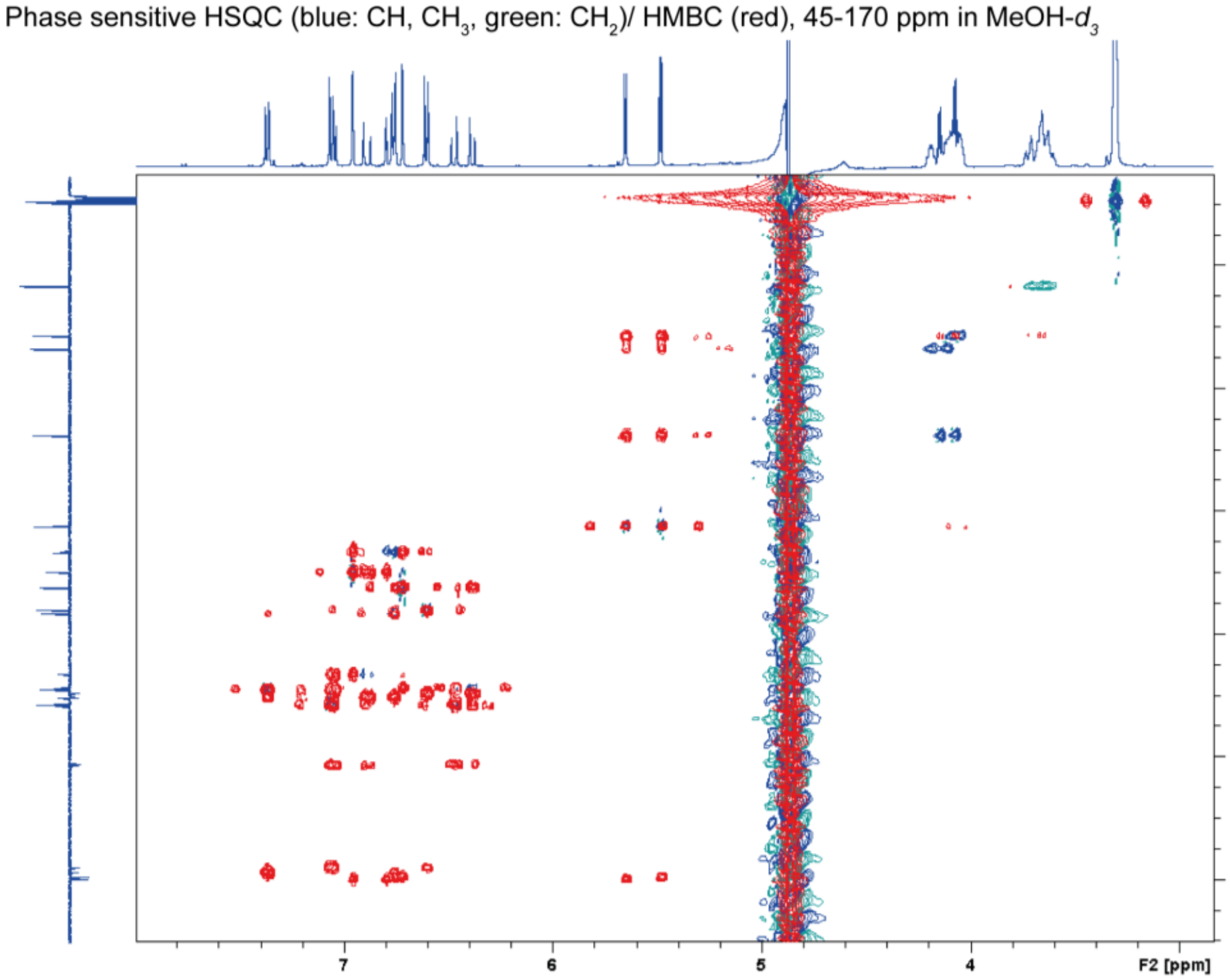

Figure S2D NMR spectra for resveratrol-3,5-*O*-α-diribofuranoside (***E*-3d** and ***Z*-3d**, ***E*** *:* ***Z*** = 1 : 1.1)

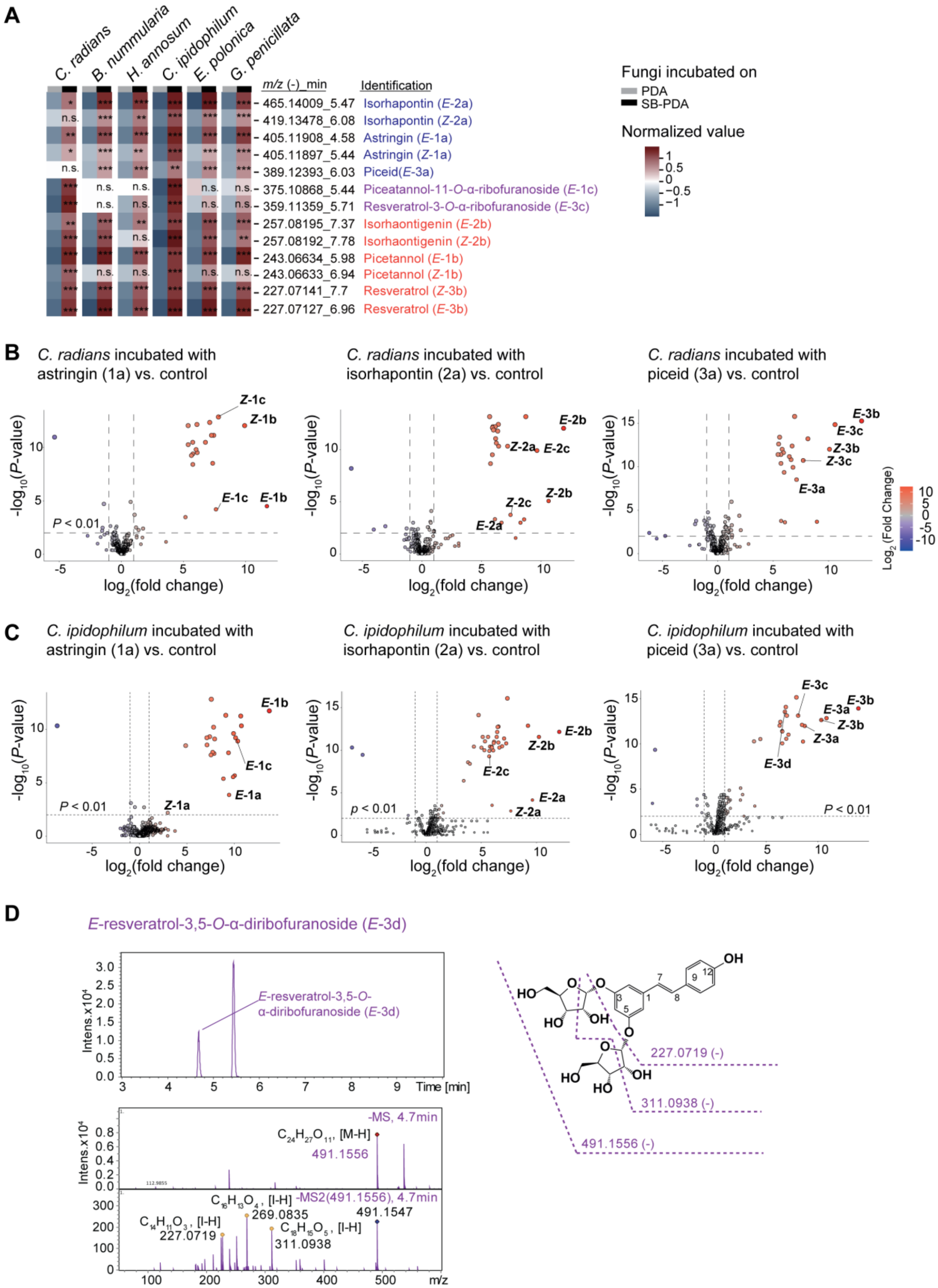

A
C. radians
B. nummularia
H. annosum
C. ipidophilum
E. polonica
G. penicillata
m/z (-)_min
Identification
465.14009_5.47 Isorhapontin (E-2a)
419.13478_6.08 Isorhapontin (Z-2a)
405.11908_4.58 Astringin (E-1a)
405.11897_5.44 Astringin (Z-1a)
389.12393_6.03 Piceid(E-3a)
375.10868_5.44 Piceatannol-11-O-α-ribofuranoside (E-1c)
359.11359_5.71 Resveratrol-3-O-α-ribofuranoside (E-3c)
257.08195_7.37 Isorhaontigenin (E-2b)
257.08192_7.78 Isorhaontigenin (Z-2b)
243.06634_5.98 Picetannol (E-1b)
243.06633_6.94 Picetannol (Z-1b)
227.07141_7.7 Resveratrol (Z-3b)
227.07127_6.96 Resveratrol (E-3b)
Fungi incubated on
PDA
SB-PDA
Normalized value
B
C. radians incubated with astringin (1a) vs. control
C. radians incubated with isorhapontin (2a) vs. control
C. radians incubated with piceid (3a) vs. control
-log10(P-value)
log2(fold change)
P < 0.01
Log2(Fold Change)
C
C. ipidophilum incubated with astringin (1a) vs. control
C. ipidophilum incubated with isorhapontin (2a) vs. control
C. ipidophilum incubated with piceid (3a) vs. control
D
E-resveratrol-3,5-O-α-diribofuranoside (E-3d)
Intens.x10⁴
Time [min]
E-resveratrol-3,5-O-α-diribofuranoside (E-3d)
-MS, 4.7min
C24H27O11, [M-H]
491.1556
-MS2(491.1556), 4.7min
C16H13O4, [I-H]
269.0835
C14H11O3, [I-H]
227.0719
C16H15O5, [I-H]
311.0938
491.1547
m/z
227.0719 (-)
311.0938 (-)
491.1556 (-)

37

Figure S3. *Coprinellus radians* and *Cylindrobasidium ipidophilum* convert stilbene glucosides into ribosylated derivatives via the aglucones. (A) Heatmap of LC–MS/MS features derived from untargeted UHPLC–qTOF–MS analyses of fungal biomass grown on SB-PDA compared with PDA lacking spruce bark (n = 5). **(B-C)** Volcano plots illustrating the metabolic transformation of phenolic glucosides by *C. radians* **(B)** and *C. ipidophilum* **(C)** compared with DMSO solvent controls (n = 5). Metabolomic data are presented as peak intensities normalized to sample weight and $\log_{10}$-transformed. Statistically significant metabolites derived from phenolic substrates are listed in the Raw Data File. ***E*-1a** and ***Z*-1a** denote the *E*- and *Z*-isomers, respectively. **(D)** Identification of (*E*)-resveratrol-3,5-O-α-diribofuranoside. Depicted are extracted ion chromatograms (*m/z* 491.1556) and mass spectra from UHPLC-qTOF-MS in the negative ionization mode. NMR spectra and assignments are shown in Supplementary Fig. S3 and Table S4. Significance was determined using Student's t-tests ($P < 0.01$) in **A-C**. Asterisks indicate significance levels (n.s., $p \geq 0.05$; *, $p < 0.05$; **, $p < 0.01$; ***, $p < 0.001$).

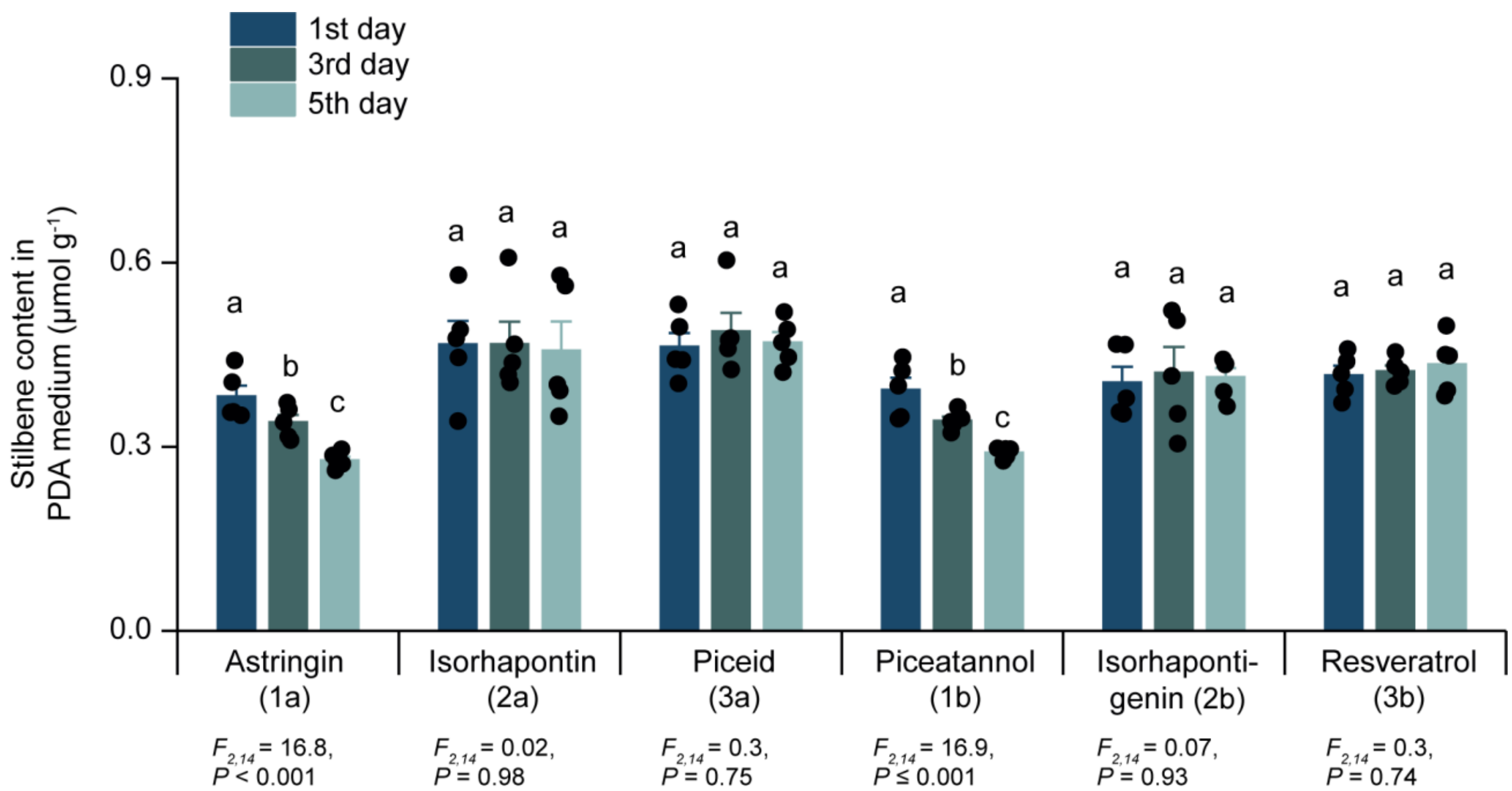

Figure S4. Time-dependent degradation of astringin and piceatannol in PDA medium in the absence of fungal incubation. Stilbene concentrations in PDA medium were quantified after 1, 3, and 5 days of incubation without fungi (n = 5). The amounts of *E*- and *Z*- isomers were combined, e.g., compound **1a** represents the combined quantity of ***E*-1a** and ***Z*-1a**. Statistically significant differences between means (± SE) were determined using one-way ANOVA followed by Fisher's LSD tests. Different lowercase letters indicate significant differences (P < 0.05).

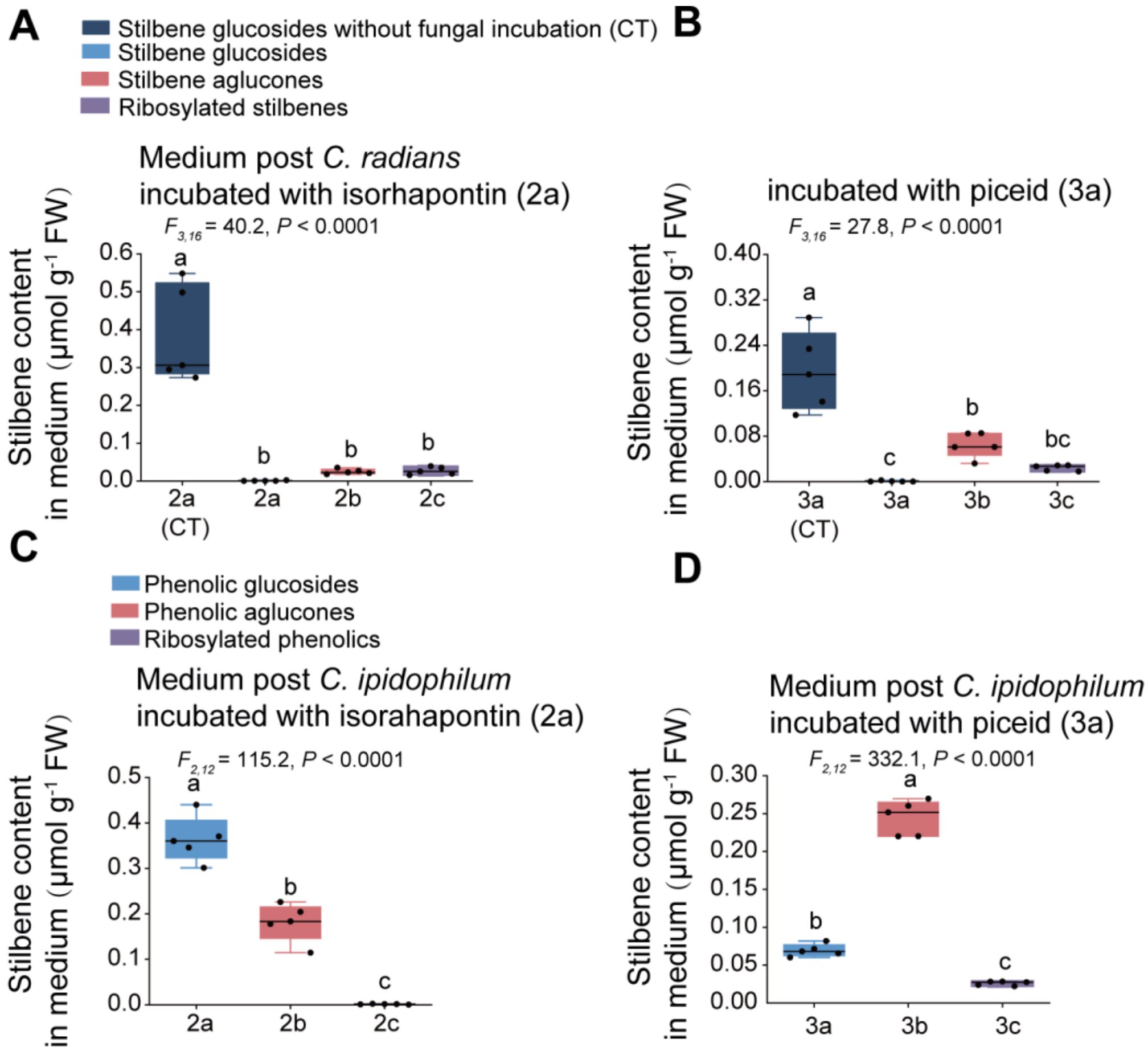

Figure S5. Metabolite content in the incubation medium following fungi growth on stilbene glucosides. (A-B) Quantification of stilbene glucosides and their derived metabolites in the incubation medium after 4 days of *C. radians* growth with isorhapontin **(A)** or piceid **(B)** as substrates (n = 5). **(C-D)** Quantification of stilbene glucosides and their derived metabolites in the incubation medium after 4 days of *C. ipidophilum* growth with isorhapontin **(C)** or piceid **(D)** as substrates (n = 5). The amounts of *E*- and *Z*- isomers were combined, e.g., compound **2a** represents the combined quantity of ***E*-2a** and ***Z*-2a**. Box plots represent the range of 25 to 75%, with center lines indicating median values and whiskers extending to 1 time the interquartile range. Statistically significant differences between means (± SE) were determined using one-way ANOVA followed by Fisher's LSD tests (P < 0.01). Different lowercase letters indicate significant differences.

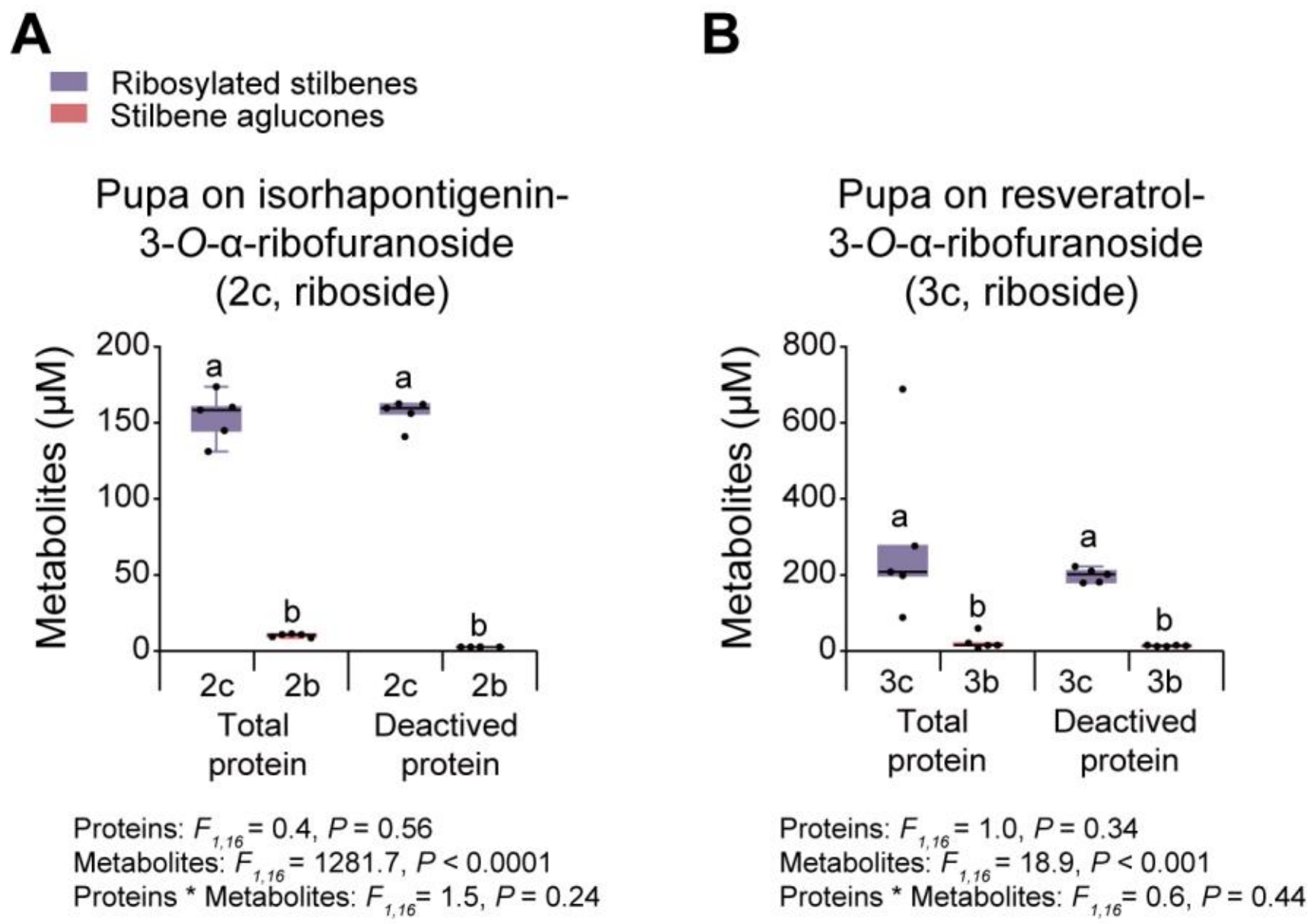

Figure S6. Ribosylation of stilbene aglucones stabilizes the compounds against hydrolysis by bark beetle proteins. (A-B) Crude protein extracts from bark beetle pupae were incubated with the ribosylated stilbenes, isorhapontigenin-3-*O*-ribofuranoside **(A)** and resveratrol-3-*O*-ribofuranoside **(B)**. Boiled, inactivated proteins served as negative controls (n = 5). The amounts of *E*- and *Z*- isomers were combined, e.g., compound **2c** represents the combined quantity of ***E*-2c** and ***Z*-2c**. Box plots represent the range of 25 to 75%, with center lines indicating median values and whiskers extending to 1 time the interquartile range. Statistically significant differences between means (± SE) were determined using two-way ANOVA followed by Fisher's LSD tests (P < 0.05). Different lowercase letters indicate significant differences.

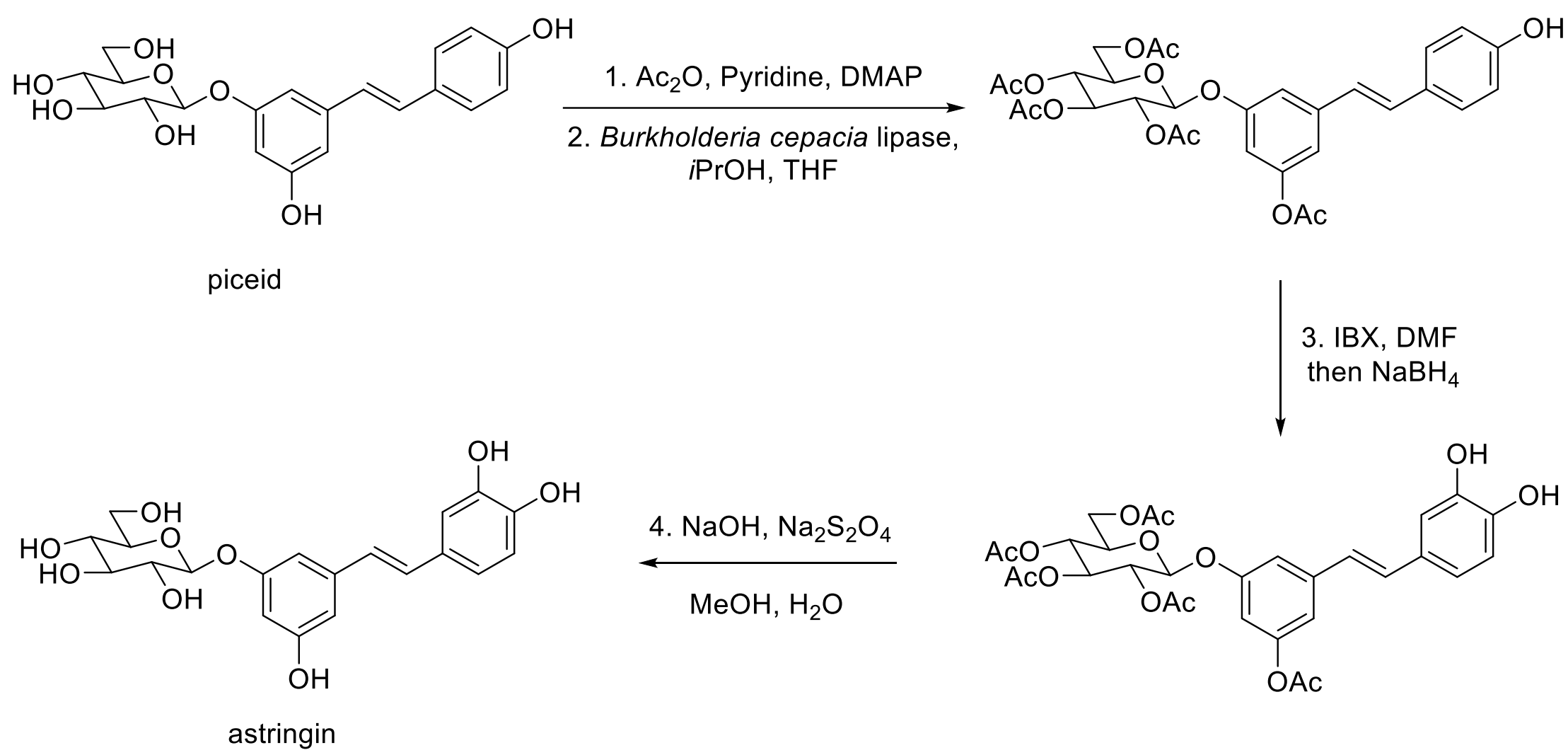

Figure S7. Brief summary of the reported method for synthesis of astringin.

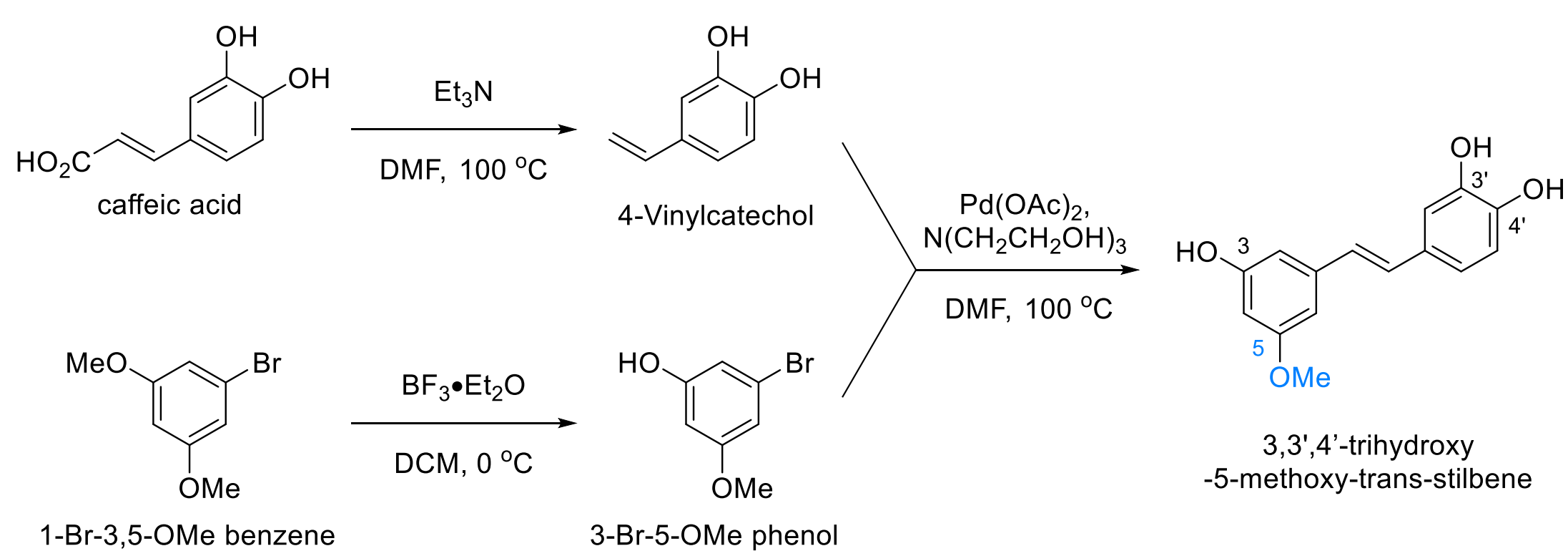

Figure S8. Synthesis of 3,3',4'-trihydroxy-5-methoxy-trans-stilbene.

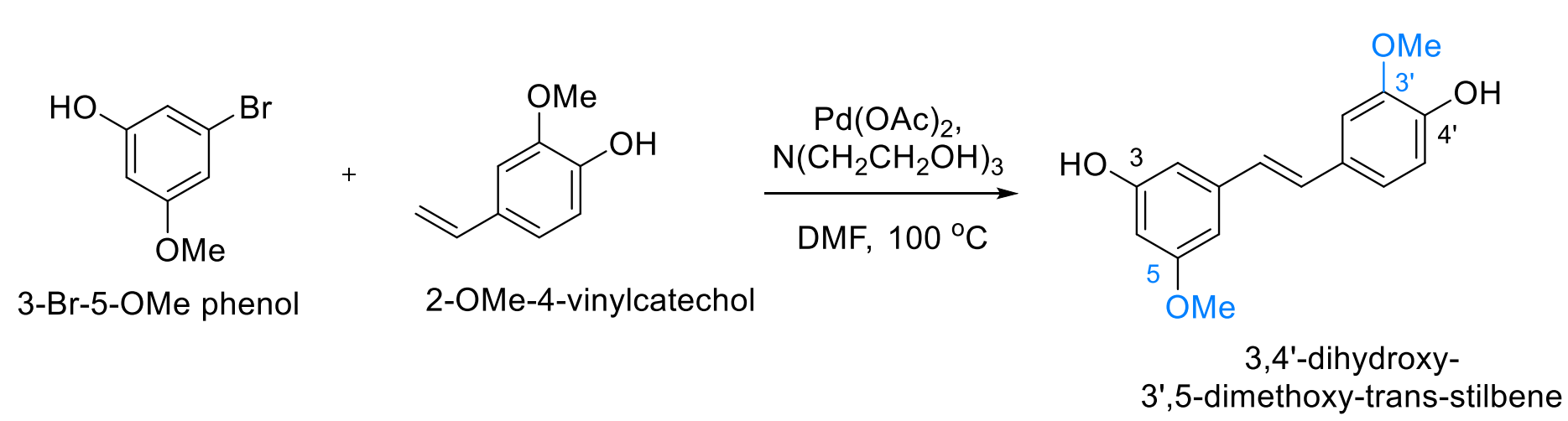

Figure S9. Synthesis of 3,4'-diihydroxy-3',5-dimethoxy-trans-stilbene.

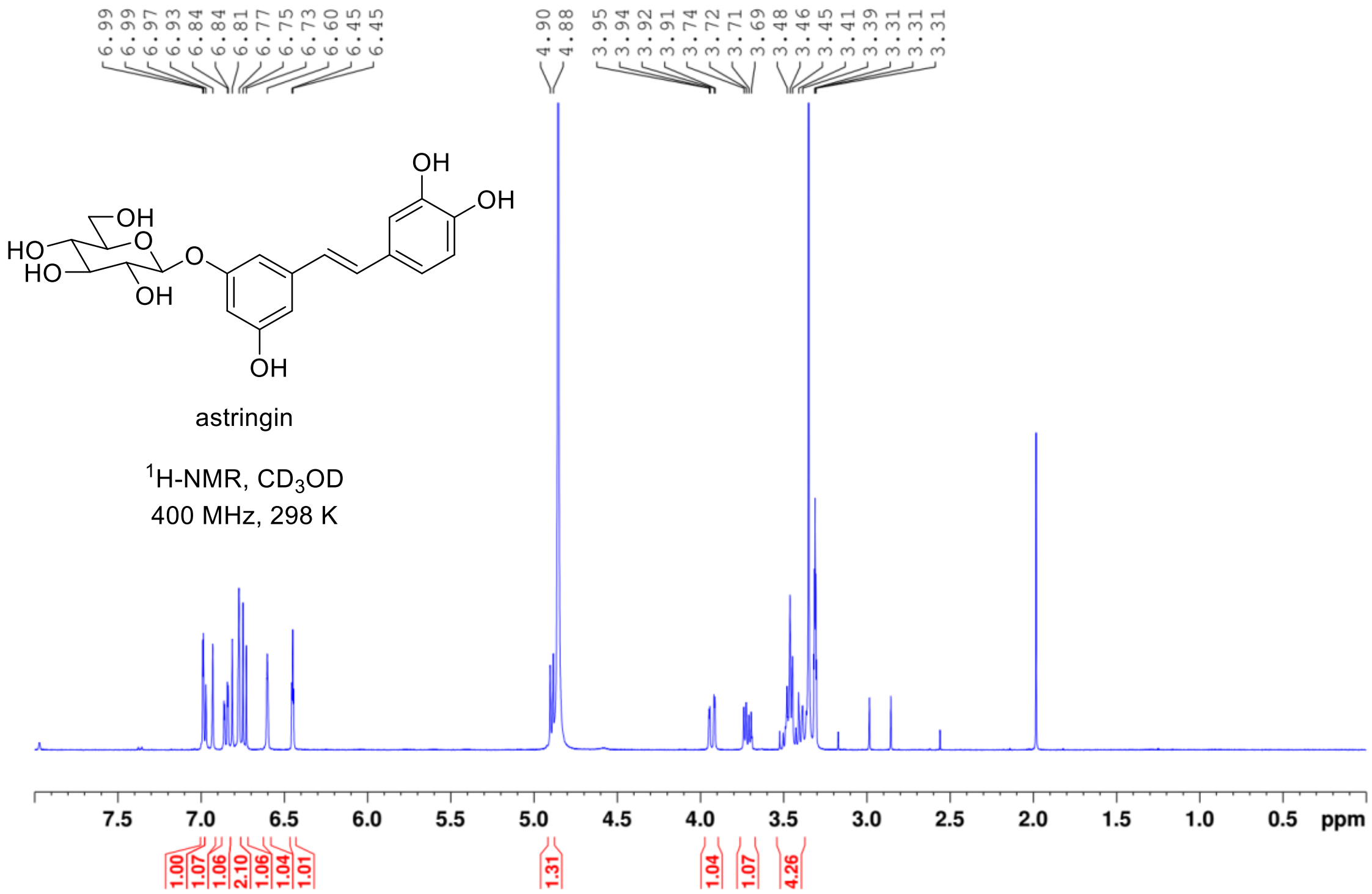

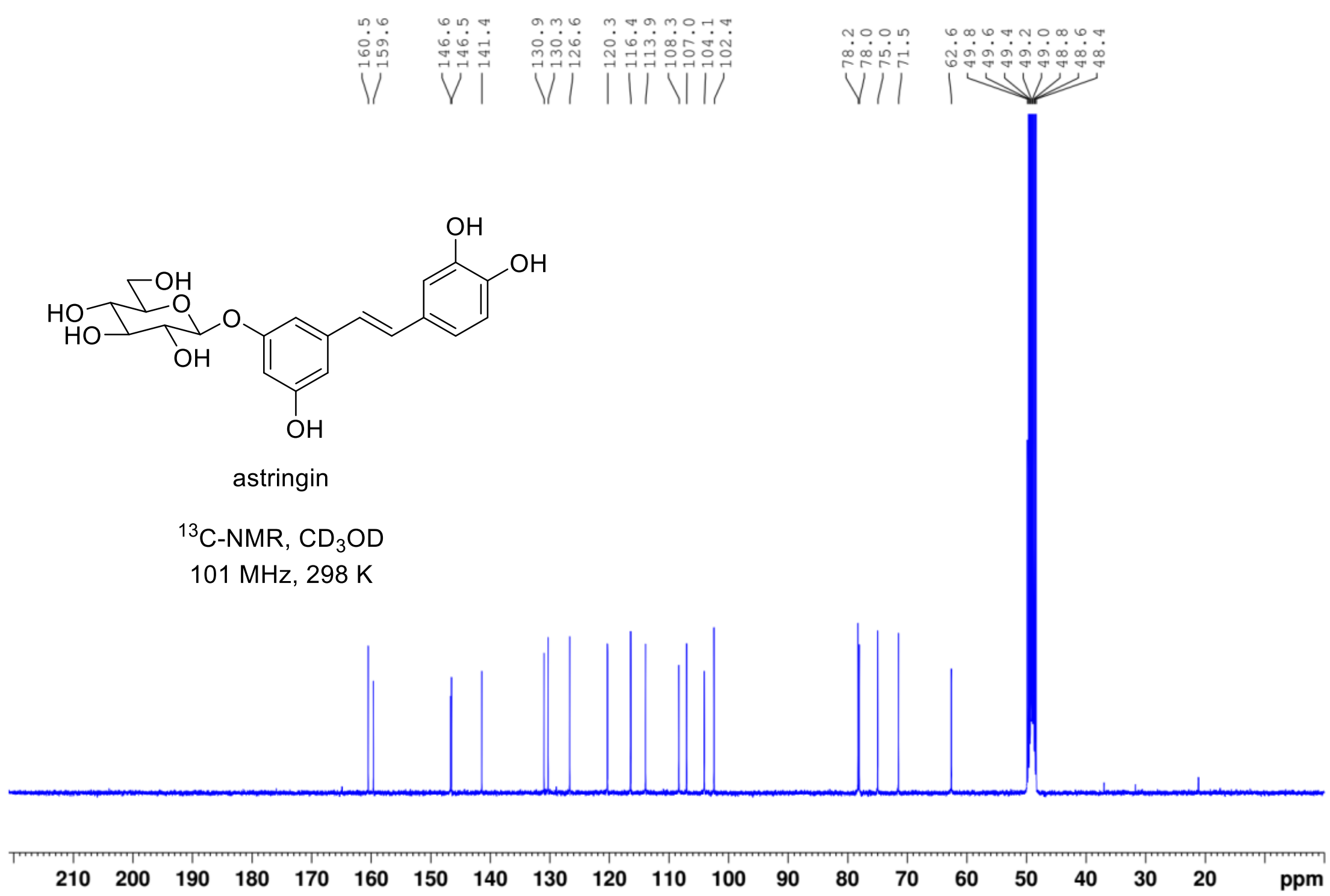

Figure S10A NMR spectra for astringin.

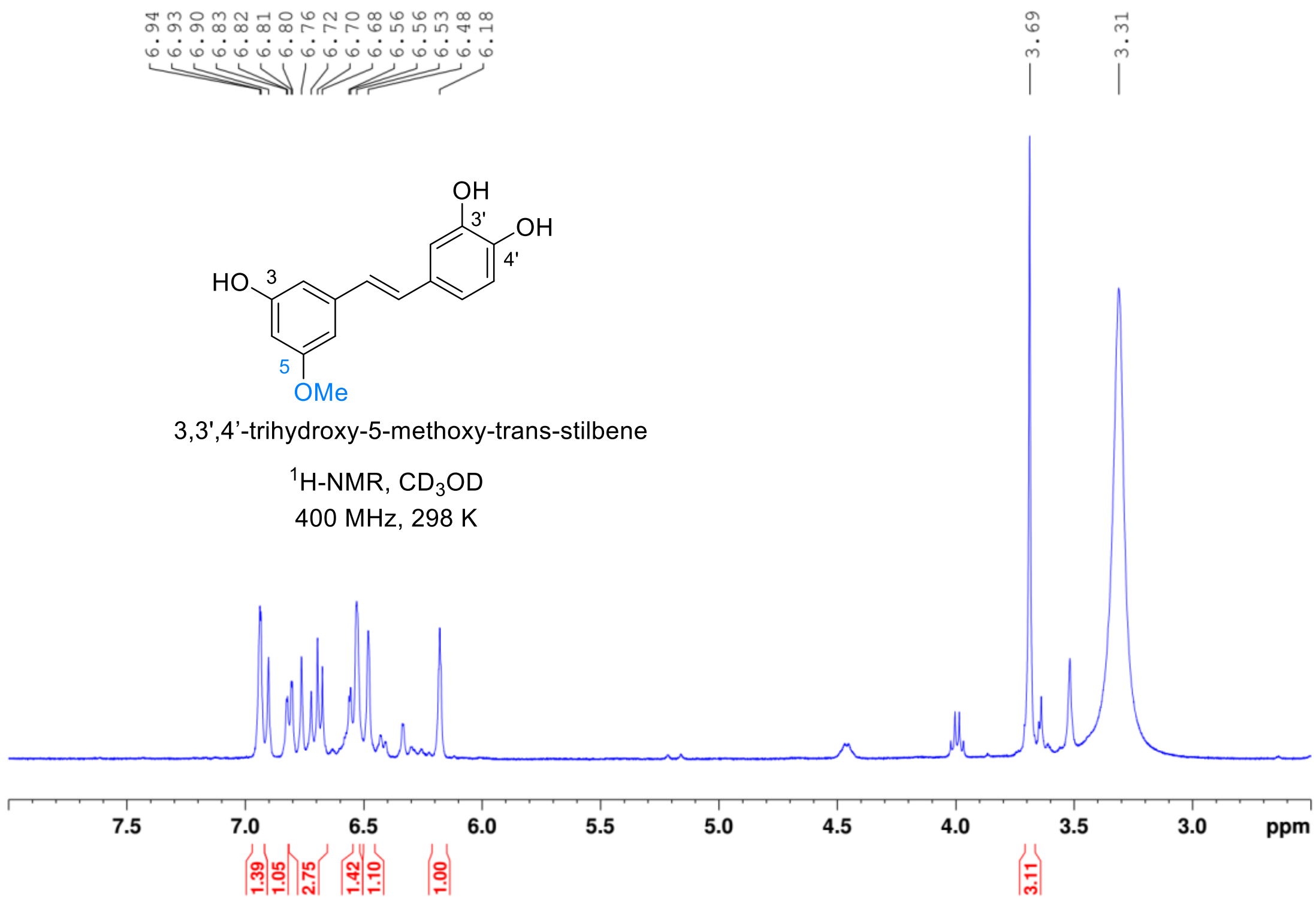

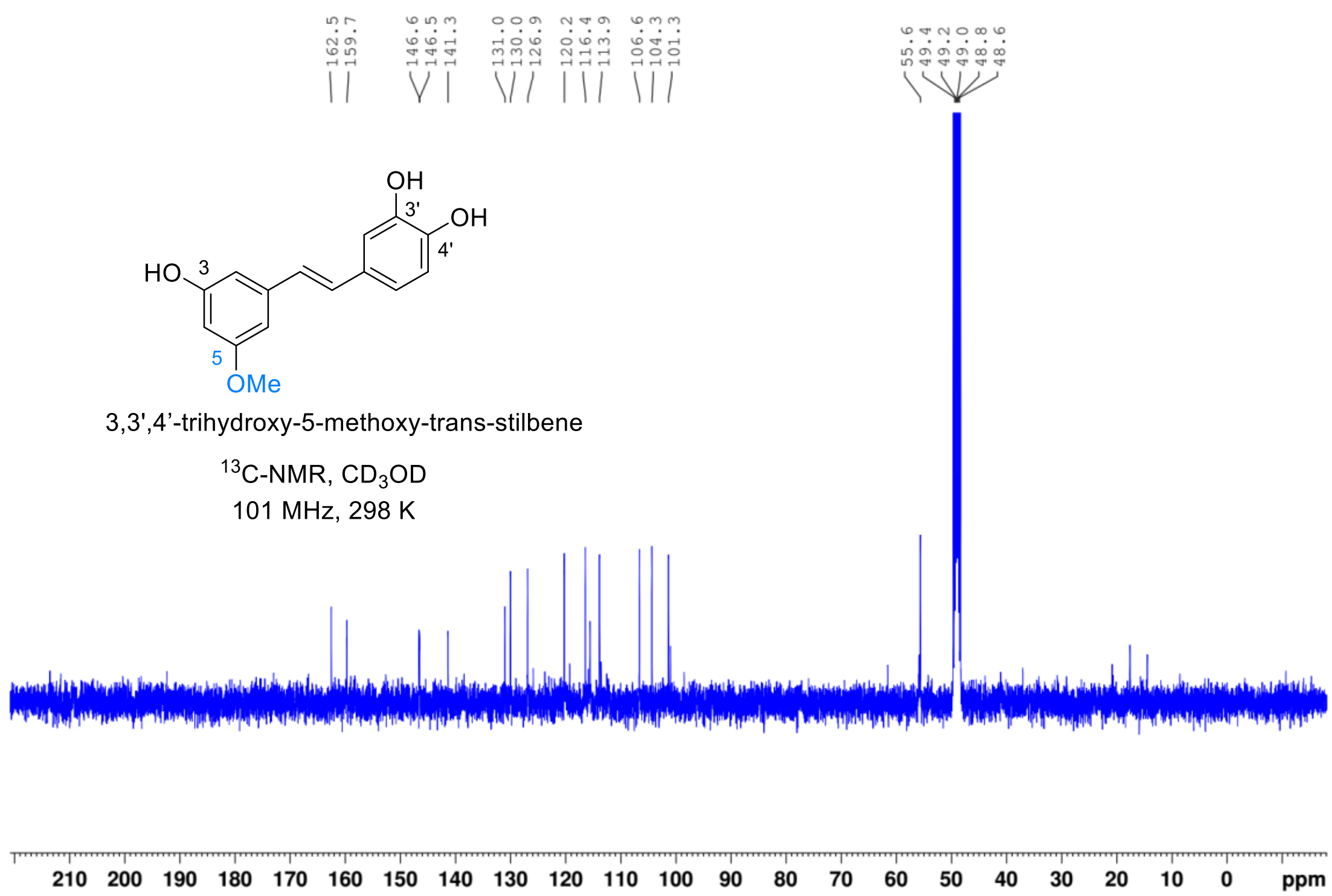

Figure S10B NMR spectra for 3,3’,4’-trihydroxy-5-methoxy-trans-stilbene.

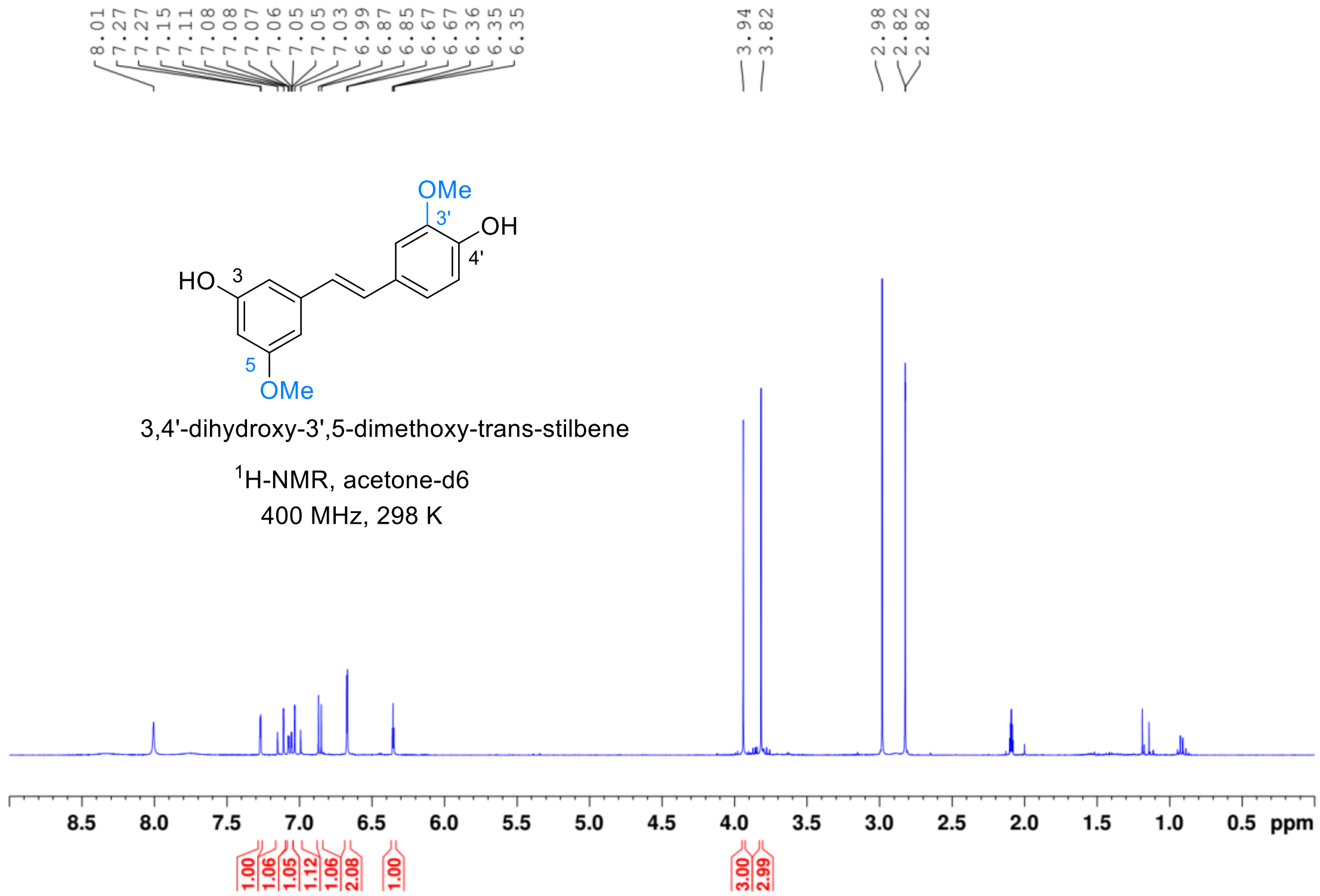

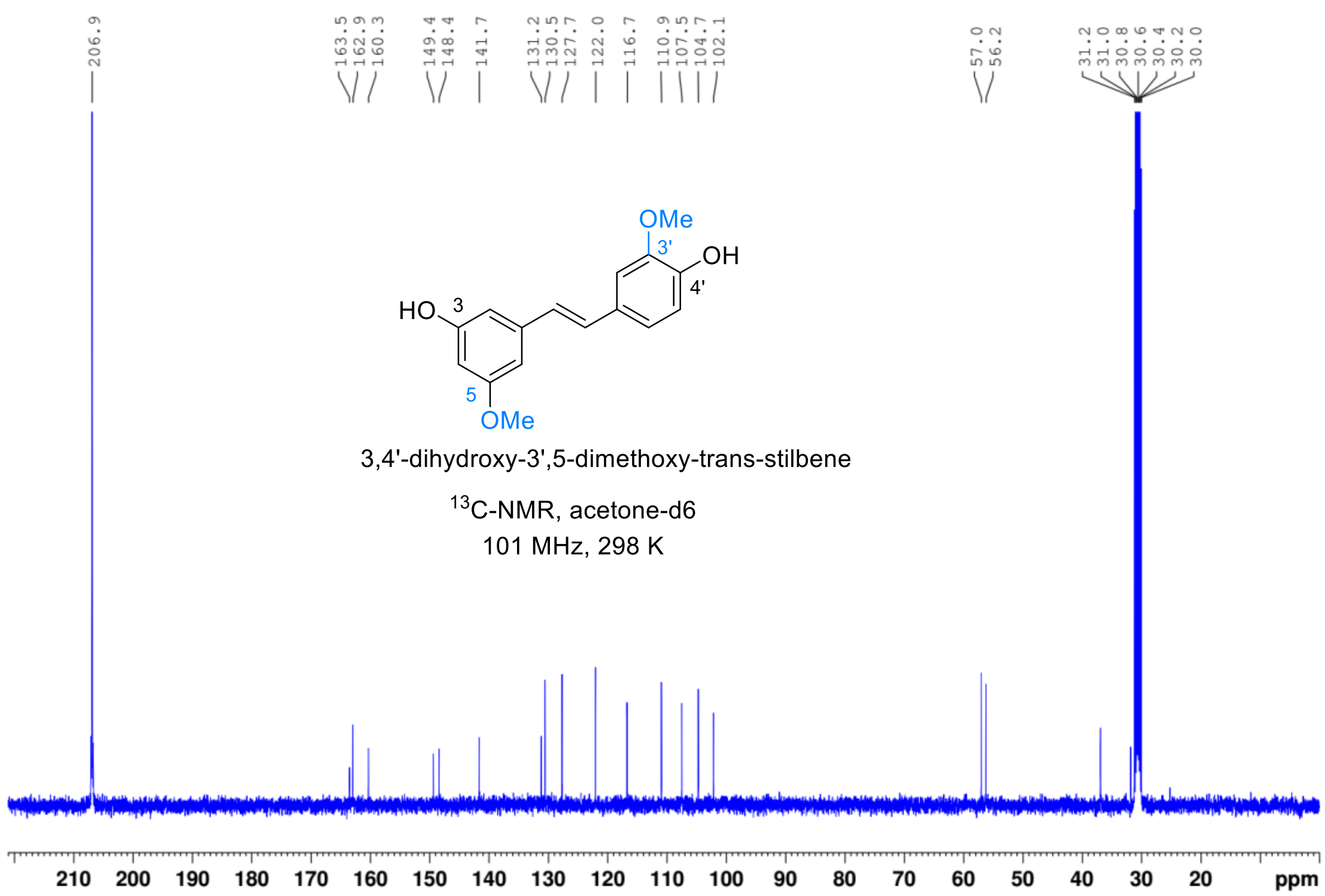

Figure S10C NMR spectra for 3,4'-diihydroxy-3',5-dimethoxy-trans-stilbene.
