# Supplementary material for: Fungi associated with Norway spruce and its bark beetles detoxify spruce defense compounds by conjugation to α-ribose
Source: ISME J. 2026 Sep 4;20(1):wrag230. doi: 10.1093/ismejo/wrag230 (PMC13637821; doi:10.1093/ismejo/wrag230)
Supplement: Supplementary file 2 [file isme-supplementary_methods-r3_wrag230.pdf]

Fungi associated with Norway spruce and its bark beetles detoxify spruce defense compounds by conjugation to α-ribose

Shivani Kiran Nimbkar, Yoko Nakamura, Baoyu Hu, Benke Hong, Beate Rothe, Michael Reichelt, Maximilian Lehenberger, Guilin Hu, Julián D. Restrepo-Leal, Xingcong Jiang, Jonathan Gershenzon, Ruo Sun

Supplementary Methods

Fungi investigated included the saprotrophs *Coprinellus radians* UF-PT141 (Agaricales: Psathyrellaceae), *Chaetomium globosum* P7-2 (Sordariales: Chaetomiaceae), *Fomitopsis pinicola* P397 (Polyporales: Fomitopsidaceae), *Nectria pseudotrichia* UF-PT101 (Hypocreales: Nectriaceae), *Pleurotus ostreatus* P148 (Agaricales: Pleurotaceae), and *Trichoderma harzianum* P338 (Hypocreales: Hypocreaceae); the endophyte *Biscogniauxia nummularia* UF-PT69 (Xylariales: Graphostromataceae); and the pathogens *Heterobasidion annosum* P402 (Russulales: Bondarzewiaceae) and *Stereum sanguinolentum* P354 (CBS 116.71) (Russulales: Stereaceae). All of these isolates, except *P. ostreatus*, were originally obtained from Norway spruce, but are not necessarily considered to be associated exclusively with spruce in the literature. Fungal associates of the Eurasian spruce bark beetle (*Ips typographus*) studied included *Cylindrobasidium ipidophilum* (Agaricales: Physalacriaceae) isolates IFFF-BOKU KF/28/3, CBS 116786, CBS 119238, and CBS 120295, as well as *Sporothrix fusiformis* (Ophiostomatales: Ophiostomataceae) isolate CMW 43723, *Ophiostoma piceae* (Ophiostomatales: Ophiostomataceae) isolate CMW 25034, *Grosmannia penicillata* (Ophiostomatales: Ophiostomataceae) isolate CMW 43738, and *Endoconidiophora polonica* (Microascales: Ceratocystidaceae) isolate MPICE-2014-1. These isolates were maintained on potato dextrose agar (PDA) medium at 25 °C.

Genomic DNA of fungi was extracted using the DNeasy Blood & Tissue Kit (Qiagen, Hilden, Germany). Fungal strains were identified by sequencing the internal transcribed spacer (ITS) regions, following cloning and PCR amplification as previously described [1]. The primer pairs used for ITS amplification are listed in Table S1. The ITS rRNA gene sequences of the fungi provided in Supplementary File 1. Taxonomic assignment was performed using the UNITE 10.0 database. The ITS rRNA gene sequence of the *G. penicillata* isolate CMW43738 was obtained from the NCBI database. Phylogenetic analysis of the ITS rRNA gene regions from these isolates was conducted by aligning nucleic acid sequences using MUSCLE. A UPGMA tree was generated in Geneious Prime, with branch labels representing consensus support (%).

Bark beetle rearing system

A continuous rearing of Eurasian spruce bark beetles (*I. typographus*, Coleoptera: Curculionidae) was carried out in freshly cut Norway spruce (*Picea abies*) logs (approximately 28 cm in diameter and 25

cm in height) within a controlled environmental chamber set at 25°C, 60% relative humidity, and a 16:8 h light: dark photoperiod [2]. A healthy Norway spruce tree was felled monthly from a state-owned forest near Jena, Germany (50°54'28.3"N 11°39'28.9"E), and the logs cut into 25 cm sections. The ends of the logs were coated in paraffin to prevent desiccation. Each log was placed individually in a plastic box with a metallic mesh on the lid for ventilation. A group of 40 adult beetles was introduced to each log, which was then kept in the controlled environmental chamber.

Collection and extraction of Norway spruce phloem

Norway spruce phloem samples, including the uninfected inner bark, fungal infected inner bark (in the absence of bark beetles), the inner bark at the margin of fungal infection (with presence of bark beetle), and inner bark adjacent to beetle galleries (in the presence of bark beetles), were collected from the rearing system. The fungal-infected spruce bark was collected from logs with fungal infection but without bark beetle tunneling. Bark beetle-tunneled spruce bark included the inner bark at the margin of fungal infection and inner bark adjacent to beetle galleries. Uninfected bark samples were collected from the inner bark without fungi and bark beetles as a control. All samples had five replicates. The samples were immediately frozen in liquid nitrogen and subsequently freeze-dried using an ALPHA 1-4 LDplus freeze dryer (Martin Christ, Osterode am Harz, Germany) for four days. The freeze-dried samples were weighed and extracted in 1 mL of 80% methanol. Each sample was homogenized by shaking with 5–6 glass beads (2.85–3.3 mm diameter) for 10 minutes on a horizontal shaker. The supernatant was collected after centrifugation at 13,000 x g for 30 minutes at 4°C. The supernatant was then transferred to 0.2 mL vials for analyses. The extracts were analyzed in an untargeted fashion using an ultra-high performance liquid chromatography system coupled to a quadrupole time-of-flight mass spectrometer (UHPLC-qTOF-MS), and analyzed for targeted phenolic compounds using liquid chromatography-tandem mass spectrometry (LC-MS/MS).

Norway spruce phleom was collected from Kranichfeld (Thuringia, Germany) to analyze the phenolic content in healthy trees and bark beetle-infested trees. Bark from healthy trees was sampled in May 2023 and July 2023. Bark from spruce trees infested by bark beetles was collected at two stages: early infestation (characterized by maternal tunnels in the bark) in May 2023, and later infestation (dead trees with crowns devoid of needles) in July 2023. Each treatment, samples were collected from two trees. Bark samples from each tree were taken at heights of 130 cm and 160 cm, with three 1 cm^2 bark samples collected at each height. The samples were immediately frozen in liquid nitrogen and subsequently freeze-dried for four days. The freeze-dried samples were weighted, extracted, and analyzed as mentioned above.

To detect if the stilbene glucosides hydrolyzed by mechanical wounding of spruce trees, spruce phloem tissues were collected. Intact phloem (around 1.5 cm^2) was collected and immediately frozen in liquid nitrogen. Large pieces of phloem (around 1.5 cm^2), small pieces of phloem (around 0.25 cm^2), and phloem thoroughly ground using a pestle were kept at room temperature for 5 hours and then frozen and

freeze-dried together with the intact phloem samples. The freeze-dried samples were weighed and extracted in 1 mL of 80% methanol and subsequently analyzed using UHPLC-qTOF-MS.

Tandem mass spectrometry (LC-MS/MS)

Targeted analyses of phenolic compounds were conducted using an Agilent HP1260 series HPLC instrument (Agilent Technologies, Böblingen, Germany) coupled to a Triple Quad 6500+ system (SCIEX, MA, USA). Phenolic compounds were separated using an Agilent Zorbax Eclipse XDB-C18 column (50 × 4.6 mm × 1.8 µm, Agilent Technologies, Wilmington, DE, USA) with mobile phase A (0.05% formic acid in Milli-Q water) and mobile phase B (acetonitrile). The elution profile was as follows: 0–7 min, 10–52% B; 7–7.01 min, 52–100% B; 7.01–8 min, 100% B; 8–8.01 min, 100–10% B; 8.01–10 min, 10% B at a flow rate of 1.1 mL/min. Detection of each compound was achieved through multiple reaction monitoring (MRM) of specific parent-to-product ion transitions for each compound. Detailed parameters are described in Table S2. Analyst 1.6.3 (SCIEX) and MultiQuant 3.0.3 software (SCIEX) were used for data acquisition and processing, respectively. Quantification of individual compounds was based on external standard calibration curves, with the sources of external standards listed in Table S3.

Untargeted metabolomic analyses using UHPLC-qTOF-MS

Metabolite extracts were analyzed using an UHPLC system (Thermo Dionex Ultimate 3000, MA, USA) coupled to a qTOFMS system (Bruker timsTOF, Bremen, Germany). The UHPLC was equipped with a Zorbax Eclipse XDB-C18 reversed-phase column (1.8 µm, 2.1 mm × 100 mm; Agilent Technologies, Böblingen, Germany), maintained at 25°C. The flow rate was set to 0.3 mL/min with a gradient elution profile using 0.1% aqueous formic acid (solvent A) and acetonitrile (solvent B) with the following profile: 5% B from 0–0.5 min, 5–60% B from 0.5–11 min, 60–100% B from 11–11.1 min, and kept at 100% B until 12 min, then re-equilibrated to 5% B from 12.1–15 min. High-resolution mass spectrometry (HRMS) was performed in either negative or positive ionization modes with automatic MS2 scans (“autoMS”) enabled. The source end plate offset was kept at 500 V and the capillary voltage at 4500 V, with the nebulizer gas at 2.8 bar, dry gas at 8 L/min and the drying temperature at 280°C. Ion transfer was performed with a funnel 1 RF of 150 Vpp, funnel 2 RF of 200 Vpp, multipole RF of 50 Vpp, and a deflection delta of 70 V, with the quadrupole ion energy maintained at 4 eV (low mass 90 m/z). The mass scan range was 50–1500 *m*/*z* at an acquisition rate of 12 Hz. Collision energies were stepped in a 50:50 timing between a collision energy of 20 eV and 50 eV, respectively. For mass spectrometer recalibration, 10 µL of a sodium formate-isopropanol solution (10 mM solution of NaOH in 50:50 (v/v%) isopropanol-water containing 0.2% formic acid) was injected into the dead volume of the sample injection to calibrate using expected cluster ion m/z values. Data processing was performed using MetaboScape 5.3 software (Bruker, Bremen, Germany) and MetaboAnalyst 6.0 (https://dev.metaboanalyst.ca/). Automated peak picking and alignment were done within a retention time range of 0.5–11.5 minutes, with a signal intensity threshold of ≥ 1000 and a maximum deviation of 2 ppm. Feature groups, potentially representing single metabolites, were consolidated into a single bucket per metabolite using

MetaboScape software. For missing data, intensities were imputed using k-nearest neighbors based on similar features for significant calculations. The data were normalized by sample weight and log_{10}-transformed for further analysis.

Spruce bark agar

To prepare spruce bark agar, fresh bark from *Picea abies* was collected and processed [3]. The bark was cut into small pieces, ground into a fine powder using a vibratory micro mill Pulverisette 0 (Fritsch GmbH, Idar-Oberstein, Germany) with liquid nitrogen, and then freeze-dried using an ALPHA 1-4 LDplus freeze dryer (Martin Christ, Osterode am Harz, Germany) for 4 days. Spruce bark agar (SB-PDA) was prepared at concentrations of 1 to 5% (w/v) of spruce bark by supplementing a standard potato dextrose agar (PDA) preparation in Milli-Q water with the corresponding percentage of spruce bark powder.

The degradation of phenolic compounds in PDA medium

To evaluate the stability of stilbene compounds, PDA medium supplemented with 400 µM astringin, isorhapontin, piceid, piceatannol, isorhapontigenin, or resveratrol without fungi incubation was collected on the 1st, 3rd, and 5th days after incubation at 25°C. Five replicates were performed. Metabolites were extracted from the weighed medium, and stilbene contents were analyzed using an LC-MS/MS system.

Tracking fungal biotransformation using $^{13}C_6$-labelled resveratrol

To trace the metabolic fate of resveratrol in *C. radians* and *C. ipidophilum*, fungi were inoculated on PDA medium supplemented with 100 µM resveratrol or 100 µM ([$^{13}C_6$]resveratrol (Table S3), labeld on the monohydroxylated aromatic ring. Cellophane sheets were placed on the solidified medium to simplified the collection of fungal biomass post-incubation. Fungal mycelium was harvested into 2 mL Eppendorf tubes after eight days of incubation at 25°C. Fungal biomass was freeze-dried and weighed. Dried fungal biomass was homogenized. To each sample (fungi and medium), 80% methanol was added for metabolite extraction. The extracted compounds were analyzed using untargeted UHPLC-qTOFMS.

Metabolism of phenolic compounds by fungi

C. radians and *C. ipidophilum* were inoculated on PDA supplemented with 300 µM or 600 µM of the glucosides astringin, piceid or isorhapontin. Plates containing 0.1% DMSO served as negative controls. Five replicates were performed for each treatment. PDA plates were overlaid with cellophane sheets to facilitate fungal collection. Fungal inoculation was performed using plugs, and plates were incubated at 25°C for four days. Metabolites from both the fungal biomass and medium were extracted and analyzed using UHPLC-qTOF-MS and LC-MS/MS.

To further test the metabolism of the stilbene compounds by Norway spruce- and *I. typographus*-associated fungi, as well as additional basidiomycete species, fungi were incubated with 200 µM of piceid. This experiment is described in the Supplementary Method in more detail.

Metabolism of piceid by Norway spruce- and bark beetle- associated fungi

The metabolism of stilbene compounds was studied in Norway spruce- and bark beetle-associated fungi, as well as additional basidiomycete species. These fungi were incubated on PDA supplemented with 200 µM of piceid at 25°C for four days. Fungal biomass was collected, freeze-dried, and weighed. Metabolites were extracted using 80% methanol and analyzed through untargeted UHPLC-qTOFMS.

Compound isolation and purification

The structures of stilbene metabolites produced by *C. ipidophilum* were confirmed through isolation, chromatographical purification, and NMR analysis. Resveratrol-3-*O*-α-ribofuranoside, resveratrol-3,5-*O*-α-biribofuranoside, isorhapontigenin-3-*O*-α-ribofuranoside, and piceatannol-11-*O*-α-ribofuranoside produced by *C. ipidophilum* were isolated from fungal cultures as follows: More than fifty PDA plates supplemented with 1 mM resveratrol, 600 µM isorhapontigenin, or 600 µM piceatannol were inoculated with *C. ipidophilum* and incubated at 25°C for 20 days. Subsequently, the fungi, along with medium, were collected and freeze-dried. The fungal samples used for compound isolation were extracted with pure methanol. The methanol extracts were concentrated using a rotary evaporator R-114 (Büchi, Essen, Germany) and mixed with 5 g of C18 material LiChroprep RP-18 (40–63 µm) (Merck, Darmstadt, Germany). The mixture together with C18 material, was loaded onto a preparative C18 column (40 g LiChroprep RP-18) that had been preconditioned with methanol, followed by MilliQ water. Subsequently, the C18 column was washed with a stepwise gradient of aqueous methanol solutions (ranging from 10%, 20%, 30%, 40%, 50% to 60% methanol). Elution of targeted compounds was monitored using an HPLC (Agilent HP1100 series, Agilent Technologies) with an ion trap mass spectrometer (ESQUIRE-6000 system, Bruker) equipped with a Nucleodur Sphinx RP column (250 × 4.6 mm × 5 µm, Macherey-Nagel, Düren, Germany). Aqueous 0.2% formic acid and acetonitrile were employed as mobile phases A and B, respectively. The elution profile was as follows: 0–20 min, 10–60% B; 20–20.10 min, 60–100% B; 20.10–22 min, 100% B; 22-22.1 min, 100–10% B; and 22.1–26 min, 10% B at a flow rate of 1.0 mL/min. MS analyses were performed separately with positive and negative ionization as described [4].

Compounds to be purified were collected from the 50% methanol fraction eluted from the SPE C18 cartridge. This fraction was further concentrated using a rotary evaporator and purified by repeated injection on an HPLC-UV system (Agilent Technologies 1100 Series) equipped with a diode-array detector, using a Nucleodur Sphinx RP column (250 × 4.6 mm × 5 µm, Macherey-Nagel). Stilbene products were detected at 320 nm and collected. Aqueous 0.05% formic acid and acetonitrile were used

as mobile phases A and B, respectively. The elution profile was as follows: 0–12 min, 20–38% B; 12–12.10 min, 38–100% B; 12.10–17 min, 100% B; 17–17.1 min, 100–20% B and 17.1–21 min, 20% B at a flow rate of 1.0 mL/min. The system was equipped with a fraction collector (Advantec SF-2120), and the collected compounds were concentrated using a rotary evaporator. The identification of the piceatannol dimer (longusol C), and resveratrol dimers (*E*-δ-viniferin and *Z*-δ-viniferin) produced by fungi was performed by comparison of NMR spectral data to those in a previous reference [5-9].

NMR analyses

NMR measurements were carried out on a 700 MHz Bruker Avance III HD spectrometer (Bruker Biospin GmbH, Rheinstetten, Germany) and a 500 MHz Bruker Avance III HD spectrometer (Bruker Biospin GmbH, Rheinstetten, Germany) using standard pulse sequences as implemented in Bruker Topspin ver. 3.6.1. 700 MHz and 500 MHz NMR equipped with a TCI cryoprobe. Chemical shifts were referenced to the residual solvent signals of MeOH-d_3 (δ_H 3.31/δ_C 49.0), DMSO-d_6 (δ_H 2.50/δ_C 39.52). All spectra were recorded at 298 K. Compounds with a stilbene skeleton can easily be isomerized by light (including a UV detector in HPLC), which makes isolating them as pure stereoisomers difficult. Therefore, they were measured and structurally determined as mixtures. Structure elucidation were performed by analyzing ^{1}H, ^{13}C (DEPTQ), COSY, HSQC, and HMBC using Bruker Topspin ver. 3.6.1. Because the structure of resveratrol-3-*O*-α-ribofuranoside has been previously reported [10], it was measured in DMSO-d_6 for comparison. The chemical shifts agreed well with the previously published results. The piceatannol dimer (longusol C), and resveratrol dimers (*E*-δ-viniferin and *Z*-δ-viniferin) produced by fungi were identified by comparison of NMR spectral data to those in previous publications [5-9].

Astringin synthesis and NMR characterization

Approximately 7.2 g astringin was synthesized according to the reported method [11]. The spectroscopic data are in accordance with literature reported values [11]. NMR spectra were measured on a 400 MHz Bruker Avance III HD spectrometer (Bruker Biospin GmbH, Rheinstetten, Germany). CD_3OD and d6-acetone were used as solvents. NMR spectra were referenced to the residual solvent signals at δ_H 3.31 and δ_C 49.0 for CD_3OD and δ_H 2.09 and δ_C 30.6 for acetone-d_6. For spectrometer control and data processing Bruker TopSpin ver. 3.6.1 was used. A brief summary of the reported method for synthesis of astringin is shown in (Fig. S7), and NMR spectra is shown in (Fig. S10A).

^{1}H-NMR (400 MHz, CD_3OD) δ 6.99 (d, *J* = 2.0 Hz, 1H), 6.95 (d, *J* = 16.3 Hz, 1H), 6.87 – 6.83 (m, 1H), 6.81-6.77 (m, 2H), 6.74 (d, *J* = 8.1 Hz, 1H), 6.60 (s, 1H), 6.45 (t, *J* = 2.1 Hz, 1H), 4.89 (d, *J* = 7.3 Hz, 1H), 3.93 (dd, *J* = 12.0, 2.1 Hz, 1H), 3.72 (dd, *J* = 12.0, 5.8 Hz, 1H), 3.48-3.39 (m, 4H);

^{13}C-NMR (101 MHz, CD_3OD) δ 160.5, 159.6, 146.6, 146.5, 141.4, 130.9, 130.3, 126.6, 120.3, 116.4, 113.9, 108.3, 107.0, 104.1, 102.4, 78.2, 78.0, 75.0, 71.5, 62.6, 49.8.

3-Br-5-OMe phenol [12] and 4-vinylcatechol [13] were prepared according to reported methods (Fig. S8 and Fig. S10B). A mixture of 3-Br-5-OMe phenol (2.58 g, 12.8 mmol), 4-Vinylcatechol (1.74 g, 12.8 mmol), triethanolamine (5.1 mL, 38.5 mmol) and Pd (II) acetate (288 mg, 1.28 mmol) in dry DMF (43 mL) was stirred under N_2 at 100°C for 24 h. Then the reaction was cooled to room temperature, quenched by the addition of aq. hydrochloride acid (1 N, 50 mL), and extracted with ethyl acetate (3 x 50 mL). The combined organic layers were concentrated in vacuo, and the crude product was purified by a column chromatography (silica gel, n-hexane/ethyl acetate = 1/1) to obtain 3,3',4'-trihydroxy-5-methoxy-trans-stilbene (620 mg, 19% yield) as a brown oil.

^{1}H-NMR (400 MHz, CD_3OD) δ 6.99 (d, *J* = 1.7 Hz, 1H), 6.93 (d, *J* = 16.2 Hz, 1H), 6.88 – 6.69 (m, 3H), 6.54 (s, 2H), 6.24 (s, 1H), 3.77 (s, 1H);

^{13}C-NMR (101 MHz, CD_3OD) δ 162.5, 159.7, 146.6, 146.5, 141.3, 131.0, 130.0, 126.9, 120.2, 116.4, 113.9, 106.6, 104.3, 101.4, 55.6.

A mixture of 3-Br-5-OMe phenol (2.6 g, 12.93 mmol), 2-OMe-4-vinylcatechol (1.94 g, 12.93 mmol), triethanolamine (5.14 mL, 38.8 mmol) and Pd (II) acetate (290 mg, 1.29 mmol) in dry DMF (45 mL) was stirred under N_2 at 100 °C for 12 h. Then the reaction was cooled to room temperature, quenched by the addition of aq. hydrochloride acid (1 N, 50 mL), and extracted with ethyl acetate (3 x 50 mL). The combined organic layers were concentrated in vacuo, and the crude product was purified by a silica gel column chromatography (n-hexane/ethyl acetate=3/1 to 1/1) to obtain 3,4'-dihydroxy-3',5-dimethoxy-trans-stilbene (490 mg, 14% yield) as a brown oil. The spectroscopic data are in accordance with literature reported values [14]. Brief summary of the synthesis of 3,4'-diihydroxy-3',5-dimethoxy-trans-stilbene is shown in (Fig. S9), and NMR spectra are shown in (Fig. S10C).

^{1}H-NMR (400 MHz, d6-acetone) δ 7.27 (d, *J* = 2.0 Hz, 1H), 7.13 (d, *J* = 16.3 Hz, 1H), 7.09 – 7.04 (m, 1H), 7.01 (d, *J* = 16.3 Hz, 1H), 6.86 (d, *J* = 8.1 Hz, 1H), 6.67 (d, *J* = 2.2 Hz, 2H), 6.35 (t, *J* = 2.2 Hz, 1H), 3.94 (s, 3H), 3.82 (s, 3H);

^{13}C-NMR (101 MHz, d6-acetone) δ 163.5, 162.9, 160.3, 149.4, 148.4, 141.7, 131.2, 130.6, 127.7, 122.0, 116.7, 110.9, 107.5, 104.7, 102.1, 57.0, 56.2.

In vitro enzyme assay of crude extracted proteins

Proteins were extracted from *C. radians* and *C. ipidophilum* cultures grown for two weeks (*n*= 5). Samples were extracted in 200 µL of ice-cold phosphate buffer (50 mM, 10% glycerol, pH 6.4) with ceramic beads using a homogenizer for 3 minutes. Homogenized samples were centrifuged at 13,000 x g at 4°C for 20 minutes, after which the clean supernatant was transferred to 1.5 ml Eppendorf tubes. Protein concentrations were measured using 5 µL of each sample with the Quick Start Bradford 1× Dye Reagent (Bio-Rad, Hercules, CA) and bovine serum albumin (Thermo Fisher Scientific, Waltham, USA) as the standard. The bark beetle extract was prepared as described previously [2].

For deglycosylation enzyme assays, 20 μg of protein from each sample was incubated with 500 μM of stilbene glycosides (piceid or isorhapontin) or ribosylated phenolic compounds (resveratrol-3-*O*-α-ribofuranoside or isorhapontigenin-3-*O*-α-ribofuranoside) in 200 μL phosphate buffer (50 mM, pH 6.4) at 30°C for 2 hours. Negative controls were prepared by boiling protein samples at 95°C for 10 minutes to inactivate enzyme activity. Reactions were terminated by transferring 10 μL of the mixture into 190 μL of pure methanol and freezing at -20°C. The samples were centrifuged at 13,000 × g at 4 °C for 30 minutes, and the resulting supernatants were transferred to 2 mL vials with inserts. Reaction products were analyzed by LC-MS/MS.

Statistical analyses

Statistical analyses were performed using Origin 2023, with figures created in both Origin 2023 and Adobe Illustrator CS5. Metabolomic data were normalized using MetaboAnalyst 6.0 (https://dev.metaboanalyst.ca). Significant differences were determined using Fisher's LSD tests in conjunction with one-way or two-way ANOVA, or two-tailed t-tests, as appropriate for the data distribution and number of groups. Specific statistical tests used are described in the respective figure legends. Differences were considered significant at $P < 0.05$, with significant differences denoted by letters on graphs.

References:

1. Raja, H.A., A.N. Miller, C.J. Pearce, et al. Fungal identification using molecular tools: a primer for the natural products research community. *J. Nat. Prod.* 2017; **80**(3):756–770. DOI: 10.1021/acs.jnatprod.6b01085.
2. Sun, R., B. Hu, Y. Nakamura, et al. Detoxification of conifer antimicrobial defenses promotes entomopathogenic fungus infection of bark beetles. *Proc. Natl. Acad. Sci. U. S. A.* 2026; **123**(1):e2525513122. DOI: 10.1073/pnas.2525513122.
3. Kandasamy, D., R. Zaman, Y. Nakamura, et al. Conifer-killing bark beetles locate fungal symbionts by detecting volatile fungal metabolites of host tree resin monoterpenes. *PLoS Biol.* 2023; **21**(2):e3001887. DOI: 10.1371/journal.pbio.3001887.
4. Sun, R., B. Hong, M. Reichelt, et al. Metabolism of plant-derived toxins from its insect host increases the success of the entomopathogenic fungus *Beauveria bassiana*. *ISME J.* 2023; **17**(10):1693–1704. DOI: 10.1038/s41396-023-01480-3.
5. Natori, Y., M. Ito, M. Anada, et al. Catalytic asymmetric synthesis of (−)-E-δ-viniferin via an intramolecular C–H insertion of diaryldiazomethane using Rh2(S-TFPTTL)4. *Tetrahedron Lett.* 2015; **56**(29):4324–4327. DOI: 10.1016/j.tetlet.2015.05.072.
6. Wilkens, A., J. Paulsen, V. Wray, et al. Structures of two novel trimeric stilbenes obtained by horseradish peroxidase catalyzed biotransformation of trans-resveratrol and (−)-ε-viniferin. *J. Agric. Food Chem.* 2010; **58**(11):6754–6761. DOI: 10.1021/jf100606p.
7. Li, C., J. Lu, X. Xu, et al. pH-switched HRP-catalyzed dimerization of resveratrol: a selective biomimetic synthesis. *Green Chem.* 2012; **14**(12):3281–3284. DOI: 10.1039/c2gc36288k.
8. Morikawa, T., F. Xu, H. Matsuda, et al. Structures and radical scavenging activities of novel norstilbene dimer, longusone A, and new stilbene dimers, longusols A, B, and C, from Egyptian herbal medicine *Cyperus longus*. *Heterocycles*. 2002; **57**(11):1983–1988.
9. Hammerbacher, A., A. Schmidt, N. Wadke, et al. A common fungal associate of the spruce bark beetle metabolizes the stilbene defenses of Norway spruce *Plant Physiol.* 2013; **162**(3):1324–1336. DOI: 10.1104/pp.113.218610.
10. Zhao, G.-Y., J.-Y. Fan, C.-P. Hua, et al. Resveratrol improves fungal ribosylation capacity through a unique mechanism. *RSC Adv.* 2015; **5**(8):5657–5663. DOI: 10.1039/C4RA12851F.
11. Yamashita, Y., A. Biard, K. Hanaya, et al. Short-step syntheses of naturally occurring polyoxygenated aromatics based on site-selective transformation. *Biosci. Biotechnol. Biochem.* 2017; **81**(7):1279–1284. DOI: 10.1080/09168451.2017.1303362.
12. Guo, R., B.P. Witherspoon, and M.K. Brown. Evolution of a strategy for the enantioselective synthesis of (-)-cajanusine. *J. Am. Chem. Soc.* 2020; **142**(11):5002–5006. DOI: 10.1021/jacs.0c00359.
13. Takeshima, H., K. Satoh, and M. Kamigaito. Scalable synthesis of bio-based functional styrene: protected vinyl catechol from caffeic acid and controlled radical and anionic polymerizations

thereof. *ACS Sustain. Chem. Eng.* 2018; **6**(11):13681–13686. DOI: 10.1021/acssuschemeng.8b04400.

14. Mitra, P., B. Shome, S. Ranjan De, et al. Stereoselective synthesis of hydroxy stilbenoids and styrenes by atom-efficient olefination with thiophthalides. *Org. Biomol. Chem.* 2012; **10**(14):2742–2752. DOI: 10.1039/C2OB06991A.
