# Supplementary material for: Fungi associated with Norway spruce and its bark beetles detoxify spruce defense compounds by conjugation to α-ribose
Source: ISME J. 2026 Sep 4;20(1):wrag230. doi: 10.1093/ismejo/wrag230 (PMC13637821; doi:10.1093/ismejo/wrag230)
Supplement: Supplementary file 3 [file isme-supplementary_tables-r3_wrag230.pdf]

Supplementary Tables

Table S1 Primers for gene cloning and qPCR.

Name	Primer (5'-->3')	Function
ITS1	TCCGTAGGTGAACCTGCGG	Internal transcribed spacer (ITS) region cloning from gDNA
ITS1F2	CTTGGTCATTTAGAGGAAGTAA	
ITS4	TCCTCCGCTTATTGATATGC	
ITS5	GGAAGTAAAAGTCGTAACAAGG	
LR3	CCGTGTTTCAAGACGGG	
NS1	GTAGTCATATGCTTGTCTC	

Table S2 Parameters used for multiple reaction monitoring (MRM) analyses on the LC-MS/MS system (Agilent HP1260 series HPLC coupled to a Triple Quad 6500+ system) in negative ionization mode. Q1, selected *m/z* of the first quadrupole; Q3, selected *m/z* of the third quadrupole; DP, declustering potential; EP, entrance potential; CE, collision energy; and CXP, collision cell exit potential.

Metabolites	Ionization mode	Q1 *m/z*	Q3 *m/z*	Retention time (min)	DP (V)	EP (V)	CE (V)	CXP (V)
Astringin	Negative mode	405	243	3.5	-20	-8	-38	-15
piceatannol		243	159	4.3	-20	-8	-34	-15
Isorhapontin		419	257	4	-20	-8	-18	-15
Isorhapontigenin		257	241	5.5	-20	-8	-28	-15
Piceid		389	227	4	-20	-8	-38	-15
Resveratrol		227	185	6	-20	-8	-28	-15
Taxifolin-3‘-*O*-glucoside		465	285	3	-20	-8	-44	-15
Taxifolin		303	125	4	-20	-8	-28	-15
Quercetin-3‘-*O*-glucoside		463	301	3.6	-20	-8	-40	-15
Quercetin		301	179	5.4	-20	-8	-28	-15
Resveratrol-3-*O*-α-ribofuranoside		359	227	4	-20	-8	-22	-15
Resveratrol-3,5-*O*-α-diribofuranoside		491	269	4	-20	-8	-34	-15
Isorhapontigenin-3-*O*-α-ribofuranoside		389	299	5	-20	-8	-22	-15
Piceatannol-11-*O*-α-ribofuranoside		375	243	4.3	-20	-8	-28	-15

Table S3 Chemical standards and compound information

Compounds	Alternative name	Supplier	CAS number or reference
Astringin		Synthesized in this paper	29884-49-9
Piceatannol		TCI, Eschborn, Germany	10083-24-6
Isorhapontin		Polyphenols	32727-29-0
Isorhapontigenin		TCI	32507-66-7
Piceid		TCI	27208-80-6
Resveratrol		TCI	501-36-0
$[^{13}C_6]$resveratrol	Resveratrol-(4-hydroxyphenyl-$^{13}C_6$)	Sigma,Taufkirchen, Germany	1185247-70-4
Taxifolin-3'-*O*-glucoside	dihydroxyquercetin -3'-*O*-β-glucopyranoside	Isolated from spruce bark	[1]
Taxifolin		TCI	480-18-2
Quercetin-3'-*O*-glucoside		MedChemExpress, Sollentuna, Sweden	19254-30-9
Quercetin		Sigma	117-39-5
Catechin		Carl Roth, Karlsruhe, Germany	154-23-4
Longusol C	Piceatannol dimer	Isolated from fungi incubated on PDA plates with phenolic aglucones	[2, 3]
(*E*)-δ-viniferin	Resveratrol dimer		[4, 5]
(*Z*)-δ-viniferin	Resveratrol dimer		[6]
Piceatannol-11-*O*-α-ribofuranoside		Isolated from *Cylindrobasidium ipidophilum* incubated with phenolic aglucones	This paper
Resveratrol-3-*O*-α-ribofuranoside			1939026-18-2 [7]
Resveratrol-3,5-*O*-α-diribofuranoside			This paper
Isorhapontigenin-3-*O*-α-ribofuranoside			This paper

Table S4A NMR data for *E*-piceatannol-11-*O*-α-ribofuranoside (*E*-1c)

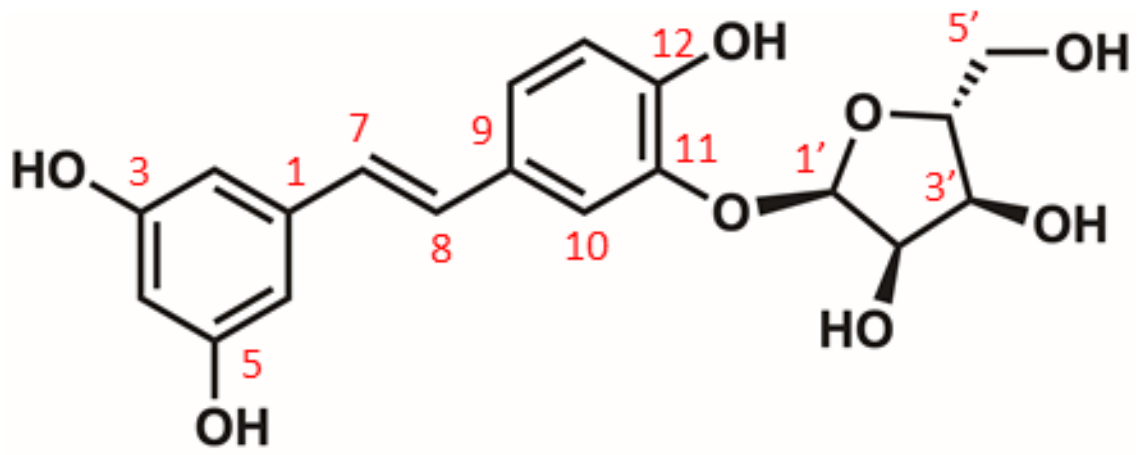

pos.	d_H	mult.	J_{HH}	d_C
1	-	-	-	141.0
2	6.43	*brs*	-	105.8
3	-	-	-	159.7
4	6.15	*brs*	-	102.8
5	-	-	-	159.7
6	6.43	*brs*	-	105.8
7	6.79	*d*	16.2	127.4
8	6.93	*d*	16.2	129.2
9	-	-	-	130.9
10	7.36	*brs*	-	115.3
11	-	-	-	146.2
12	-	-	-	148.6
13	6.81	*m**	-	116.9
14	7.04	*d*	7.3	122.7
1'	5.66	*m*	-	103.2
2'	4.25	*m*	-	73.8
3'	4.14	*m**	-	71.4
4'	4.20	*m*	-	88.2
5'a	3.72	*brd*	11.8	63.2
5'b	3.66	*brd*	11.8	63.2

* overlapped signals *J* unresolved, 700 MHz in MeOH-d_3

Table S4B NMR data for *Z*-piceatannol-11-*O*-α-ribofuranoside (*Z*-1c)

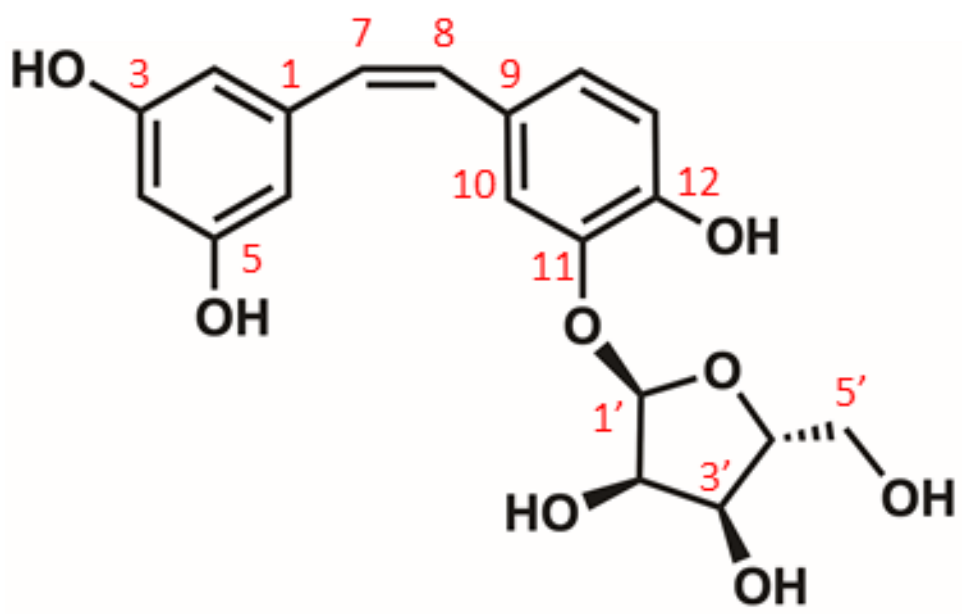

pos.	d_H	mult.	J_{HH}	d_C
1	-	-	-	140.8
2	6.22	*brs*	-	108.3
3	-	-	-	159.4
4	6.10	*brs*	-	102.4
5	-	-	-	159.4
6	6.22	*brs*	-	108.3
7	6.32	*d*	12.0	129.5
8	6.39	*d*	12.0	130.7
9	-	-	-	130.2
10	7.16	*brs*	-	118.3
11	-	-	-	145.4
12	-	-	-	147.9
13	6.69	*d*	7.7	116.3
14	6.78	*m**	-	125.0
1'	5.36	*m*	-	103.0
2'	4.14	*m*	-	73.7
3'	4.08	*m**	-	71.3
4'	4.09	*m**	-	88.0
5'a	3.64	*brd*	11.9	63.2
5'b	3.60	*brd*	11.9	63.2

* overlapped signals *J* unresolved, 700 MHz in MeOH-d_3

Table S4C NMR data for *E*-isorhapontigenin-3-*O*-α-ribofuranoside (*E*-2c)

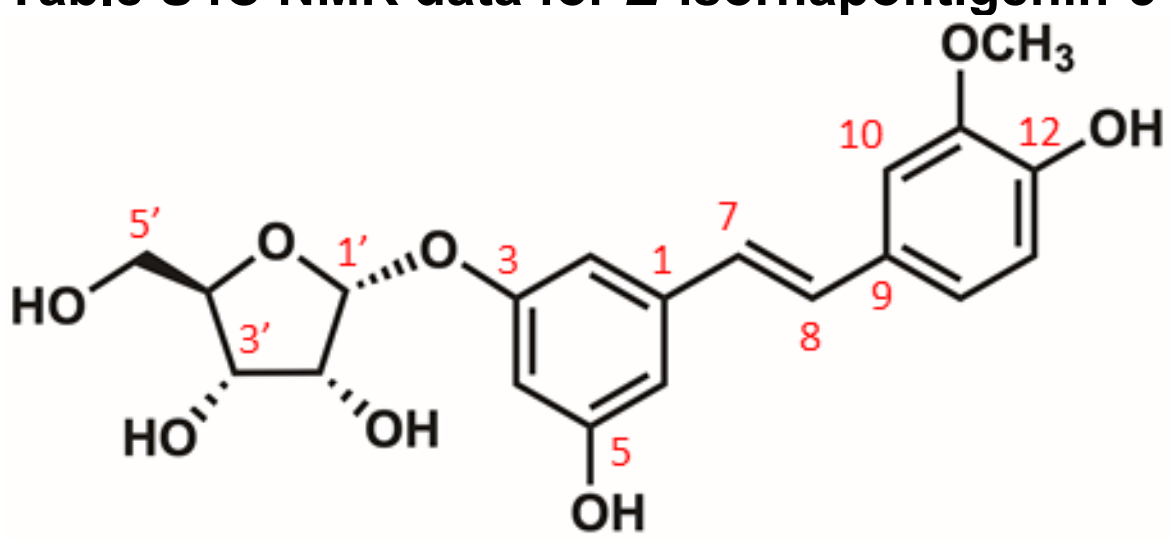

pos.	d_H	mult.	J_{HH}	d_C
1	-	-	-	141.2
2	6.80	*brs*	-	107.5
3	-	-	-	160.1
4	6.49	*brs*	-	104.8
5	-	-	-	159.6
6	6.62	*brs*	-	108.3
7	6.86	*d*	16.2	127.0
8	7.00	*d*	16.2	130.1
9	-	-	-	130.9
10	7.10	*brs*	-	110.4
11	-	-	-	149.3
12	-	-	-	147.8
13	6.76	*d*	8.1	116.4
14	6.95	*dd*	8.1/1.2	121.3
11-OMe	3.89	*s*	-	56.4
1'	5.62	*d*	4.2	102.4
2'	4.19	*dd*	6.2/4.2	73.4
3'	4.09	*dd*	6.2/3.3	71.4
4'	4.16	*ddd*	3.7/3.5/3.3	87.5
5'a	3.73	*dd*	11.9/3.5	63.3
5'b	3.66	*dd*	11.9/3.7	63.3

700 MHz in MeOH-d_3

Table S4D NMR data for *E*-resveratrol-3-*O*-α-ribofuranoside (*E*-3c)

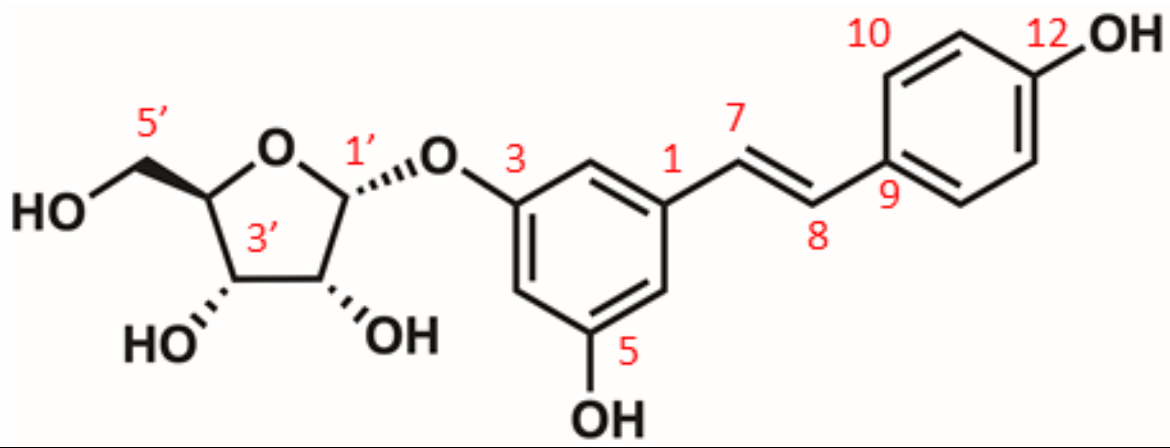

pos.	d_H	mult.	J_{HH}	d_C
1	-	-	-	139.2
2	6.66	*brs*	-	105.4
3	-	-	-	158.5
4	6.37	*dd*	2.0	103.2
5	-	-	-	158.4
6	6.56	*brs*	-	106.7
7	6.86	*d*	16.3	125.3
8	6.99	*d*	16.3	128.4
9	-	-	-	127.2
10	7.40	*brd**	8.2	127.9
11	6.75	*brd**	8.2	115.6
12	-	-	-	157.4
13	6.75	*brd**	8.2	115.6
14	7.40	*brd**	8.2	127.9
1'	5.53	*d*	4.6	103.3
2'	4.04	*dd*	6.2/4.6	71.5
3'	3.91	*dd*	6.2/3.0	69.3
4'	3.96	*ddd*	3.6/3.6/3.0	86.1
5'a	3.47	*m***	-	61.6
5'b	3.47	*m***	-	61.6
2'-OH	4.64	*brs*	-	-
3'-OH	4.88	*brs*	-	-
5'-OH	4.80	*brs*	-	-

*1,4-substituted aromatic ring, ** overlapped signals J unresolved, 700 MHz in DMSO-d_6

Table S4E NMR data for *E*-resveratrol-3,5-*O*-α-diribofuranoside (*E*-3d)

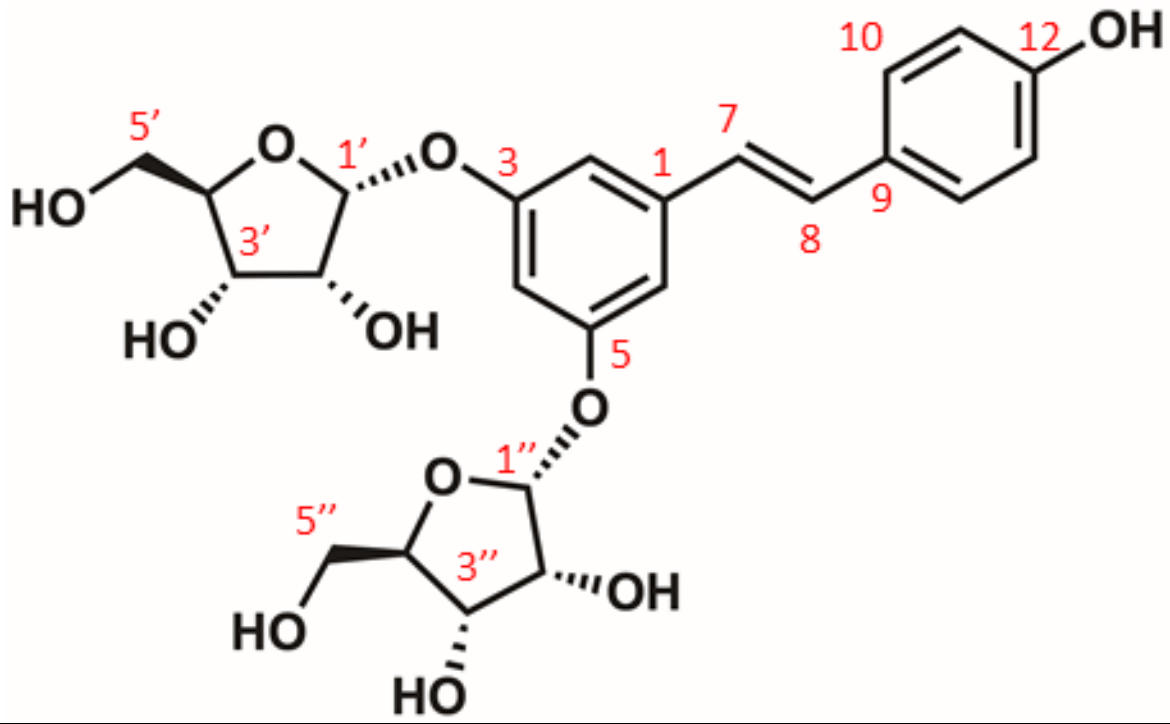

pos.	d_H	mult.	J_{HH}	d_C
1	-	-	-	141.2
2	6.96	*d*	2.1	109.7
3	-	-	-	159.7
4	6.80	*dd*	2.1/2.1	106.3
5	-	-	-	159.7
6	6.96	*d*	2.1	109.7
7	6.89	*d*	16.3	126.3
8	7.05	*d*	16.3	130.2
9	-	-	-	130.1
10	7.37	*brd**	8.6	128.8
11	6.76	*brd**	8.6	116.5
12	-	-	-	158.6
13	6.76	*brd**	8.2	116.5
14	7.37	*brd**	8.2	128.8
1'	5.65	*d*	4.3	102.28
2'	4.19	*m***	-	73.4
3'	4.11	*m***	-	71.24
4'	4.15	*m***	-	87.5
5'a	3.72	*m***	-	63.2
5'b	3.67	*m***	-	63.2
1"	5.65	*d*	4.3	102.28
2"	4.19	*m***	-	73.4
3"	4.11	*m***	-	71.24
4"	4.15	*m***	-	87.5
5"a	3.72	*m***	-	63.2
5"b	3.67	*m***	-	63.2

*1,4-substituted aromatic ring, ** overlapped signals J unresolved, 500 MHz in MeOH-d_3

Table S4F NMR data for *Z*-resveratrol-3,5-*O*-α-diribofuranoside (*Z*-3d)

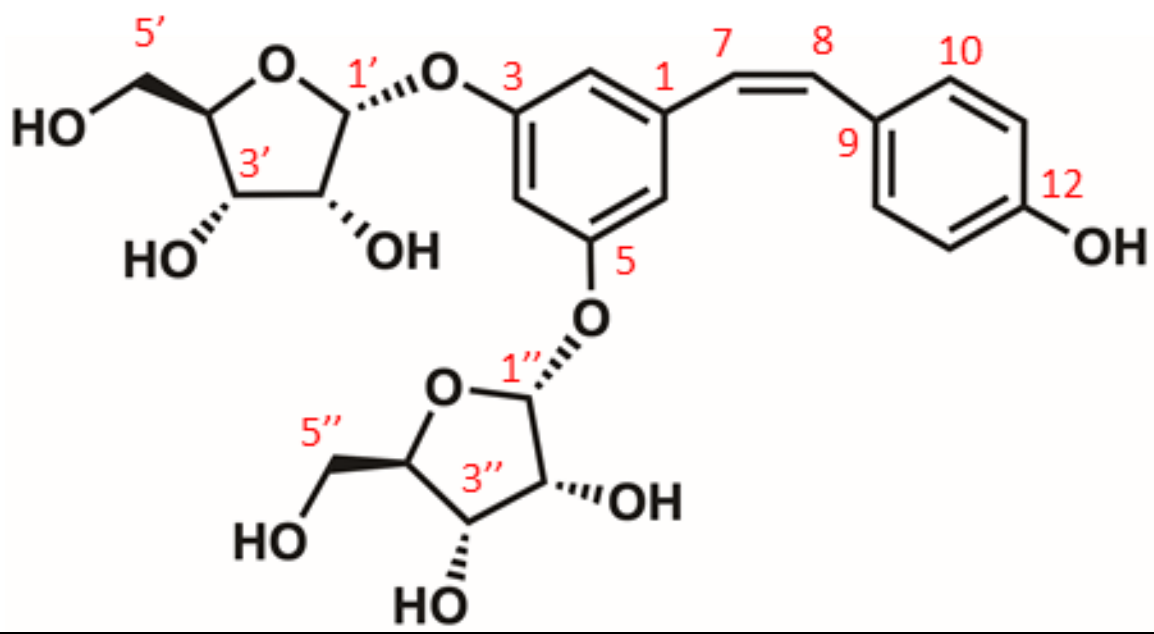

pos.	d_H	mult.	J_{HH}	d_C
1	-	-	-	141.0
2	6.72	*d*	2.1	112.2
3	-	-	-	159.4
4	6.76	*dd*	2.1/2.1	106.5
5	-	-	-	159.4
6	6.72	*d*	2.1	112.2
7	6.39	*d*	12.1	128.6
8	6.47	*d*	12.1	131.5
9	-	-	-	129.4
10	7.06	*brd**	8.6	131.3
11	6.61	*brd**	8.6	115.9
12	-	-	-	157.9
13	6.61	*brd**	8.2	115.9
14	7.06	*brd**	8.2	131.3
1'	5.48	*d*	4.4	102.26
2'	4.11	*m***	-	73.38
3'	4.05	*m***	-	71.18
4'	4.08	*m***	-	87.4
5'a	3.67	*m***	-	63.15
5'b	3.62	*m***	-	63.15
1"	5.48	*d*	4.4	102.26
2"	4.11	*m***	-	73.38
3"	4.05	*m***	-	71.18
4"	4.08	*m***	-	87.4
5"a	3.67	*m***	-	63.15
5"b	3.62	*m***	-	63.15

*1,4-substituted aromatic ring, ** overlapped signals J unresolved, 500 MHz in MeOH-d_3

References:

1. Sun, R., B. Hu, Y. Nakamura, et al. Detoxification of conifer antimicrobial defenses promotes entomopathogenic fungus infection of bark beetles. *Proc. Natl. Acad. Sci. U. S. A.* 2026; **123**(1):e2525513122. DOI: 10.1073/pnas.2525513122.
2. Hammerbacher, A., A. Schmidt, N. Wadke, et al. A common fungal associate of the spruce bark beetle metabolizes the stilbene defenses of Norway spruce *Plant Physiol.* 2013; **162**(3):1324–1336. DOI: 10.1104/pp.113.218610.
3. Morikawa, T., F. Xu, H. Matsuda, et al. Structures and radical scavenging activities of novel norstilbene dimer, longusone A, and new stilbene dimers, longusols A, B, and C, from Egyptian herbal medicine *Cyperus longus*. *Heterocycles*. 2002; **57**(11):1983–1988.
4. Natori, Y., M. Ito, M. Anada, et al. Catalytic asymmetric synthesis of (−)-E-δ-viniferin via an intramolecular C–H insertion of diaryldiazomethane using Rh2(S-TFPTTL)4. *Tetrahedron Lett.* 2015; **56**(29):4324–4327. DOI: 10.1016/j.tetlet.2015.05.072.
5. Wilkens, A., J. Paulsen, V. Wray, et al. Structures of two novel trimeric stilbenes obtained by horseradish peroxidase catalyzed biotransformation of trans-resveratrol and (−)-ε-viniferin. *J. Agric. Food Chem.* 2010; **58**(11):6754–6761. DOI: 10.1021/jf100606p.
6. Li, C., J. Lu, X. Xu, et al. pH-switched HRP-catalyzed dimerization of resveratrol: a selective biomimetic synthesis. *Green Chem.* 2012; **14**(12):3281–3284. DOI: 10.1039/c2gc36288k.
7. Zhao, G.-Y., J.-Y. Fan, C.-P. Hua, et al. Resveratrol improves fungal ribosylation capacity through a unique mechanism. *RSC Adv.* 2015; **5**(8):5657–5663. DOI: 10.1039/C4RA12851F.
