# Supplementary material for: Fungi associated with Norway spruce and its bark beetles detoxify spruce defense compounds by conjugation to α-ribose
Source: ISME J. 2026 Sep 4;20(1):wrag230. doi: 10.1093/ismejo/wrag230 (PMC13637821; doi:10.1093/ismejo/wrag230)
Supplement: Supplementary file 4 [file supplementary_file_1_wrag230.pdf]

Supplementary File 1: Internal transcribed spacer (ITS) of fungal isolates

>*Trichoderma harzianum* P338 Length: 1047bp

TCTCCCGCATATGTATATGAGCGATTCCCGCACGATAAATTCGAGTGGTTAGGGGGCCCGCGCCCG
TGGATGCATTGCGGAGGTGACCGCCTGTATTGAGAGCCACCGTAGGGGGCATCCCCAACGCGACC
CCGGAGTGTGAGTAAATGATGTGACAGCATGCCGCCAGAATACTGCGCGCAATGTGCGTCAAGATT
GATGATTCACGATTCTGCAATTCACATTACTATCGCATTCGTGCTTTTTCATCGATGCCAGAACCAAG
ATCCGTGTGAAAGTTTGATTCATTTCCAAAGGCATCGAGAGCAGGATAAGTCTCAGATTATAAATAAG
CAGCGGAGGGTATACAATGTGTTTAGTTGTGCGTCCGGGCCCACTCCGGGCTGTGACGCACCCGG
GGCAGAGATCCCGCCGAGGCAACAGTTTGTAACGTTCACATTAAGTTTGGGAGTGTAAATTTCCGTA
GGGGGCCTGCGGAGGGATCATTACCGAGTTTACAACTCCCAAACCCAATGTGAACGTTACCAAACT
GTTGCCTCGGCGGGATCTCTGCCCCGGGTGCGTCGCAGCCCCGGACCAAGGCGCCCGCCGGAGG
ACCAACCAAAACTCTTATTGTATACCCCCTCGCGGGTTTTTTTATAATCTGAGCCTTCTCGGCGCCTC
TCGTAGGCGTTTCGAAAATGAATCAAAACTTTCAACAACGGATCTCTTGGTTCTGGCATCGATGAAGA
ACGCAGCGAAATGCGATAAGTAATGTGAATTGCAGAATTCAGTGAATCATCGAATCTTTGAACGCACA
TTGCGCCCGCCAGTATTCTGGCGGGCATGCCTGTCCGAGCGTCATTTCAACCCTCGAACCCCTCCG
GGGGGTCGGCGTTGGGGATCGGCCCTCCCTTAGCGGGTGGCCGTCTCCGAAATACAGTGGCGGTC
TCGCCGCAGCCTCTCCTGCGCAGTAGTTTGCACACTCGCATCGGGAGCGCGGCGCGTCCACAGCC
GTTAAACACCCAACTTCTGAAATGTTGACCTCGGATCAGGTAGGAATGCCTC

Taxonomic assignment was performed using the UNITE 10.0 database, which identified the sequence as the following organism:

Original source

GenBank: KC403944 | ENA: KC403944

Organism

Trichoderma harzianum

Lineage

Eukaryota; Fungi; Dikarya; Ascomycota; Pezizomycotina; Sordariomycetes; Hypocreomycetidae; Hypocreales; Hypocreaceae; Trichoderma

>*Stereum sanguinolentum* P354 (CBS 116.71) Length: 905bp

CGATTCCCAAGAACCCCCGCAAAGGTGCACAGGTGTGTGGATTAGTGAAAGAGGTGTGCACATG
CCGTTTTAAAGGCCAGCTACAACCTCAGTCATAAATTTCATTAATGATCCTTCCGCAGGTTCACCTAC
GGAAAAGGATCATTAATGAAATTTATGACTGAGGTTGTAGCTGGCCTTTAAAACGGCATGTGCACAC
CTCTTTCACTAATCCACACACACCTGTGCACCTTTGCGGGGGTTCTTGGGAATCGGCGCAAGTCGTA
GTACCGGAGCTCGCGTCCCTTTACACACCCCTTTGTATGTCTTAAGAATGTCTACTCGATGTAATAAA
ACGCATCTAATACAACTTTCAACAACGGATCTCTTGGCTCTCGCATCGATGAAGAACGCAGCGAAAT
GCGATAAGTAATGTGAATTGCAGAATTCAGTGAATCATCGAATCTTTGAACGCACCTTGCGCCCTTTG
GTATTCCGAAGGGCACACCTGTTTGAGTGTCGTGAAATTCTCAACCCTCTTCACTTTTGTGAAGCTAG
GGCTTGGACTTGGAGGCTTTGCCGGCTTTGCTCGGCTCCTCTCAAATGCATTAGTACGTCTTGTTGC
GACGTGCGCCTCGGTGTGATAATTATCTACGCTGTGGTGCGCTTGCTTCTGTGGAGACGCGCTTTCA
AACCGTCCGAAAGGACAACTTTCATCGAACTTTGACCTCAAATCAGGTGGGACTACCCGCTGAACTT
AAGCATATCAATAAGCGGAGGAAAAGAAACTAACAAGGATTCCCCTAGTAACTGCGAGTGAAGCGGG
AAAAGCTCAAATTTAAAATCTGGCGGTCTTATGGCCGTCCGAGTTGTAGTCTGGAGAAGCGTTTTCC
GCGTTGGACCGTGTACAAGTTTCCTGGAACG

Taxonomic assignment was performed using the UNITE 10.0 database, which identified the sequence as the following organism:

Original source

GenBank: AF533962 | ENA: AF533962

Organism

Stereum sanguinolentum

Lineage

Eukaryota; Fungi; Dikarya; Basidiomycota; Agaricomycotina; Agaricomycetes; Russulales; Stereaceae; Stereum

>*Pleurotus ostreatus* P148 Length: 858bp

ACCTGCGGAAGGATCATTAATGAATTCACTATGGAGTTGTTGCTGGCCTCTAGGGGCATGTGCACGC
TTCACTAGTCTTTCAACCACCTGTGAACTTTTGATAGATCTGTGAAGTCGTCTCTCAAGTCGTCAGAC
TTGGTTGCTGGGATTTAAACGTCTCGGTGTGACTACGCAGTCTATTTACTTACACACCCCAAATGTAT
GTCTACGAATGTCATTTAATGGGCCTTGTGCCTTTAAACCATAATACAACTTTCAACAACGGATCTCTT
GGCTCTCGCATCGATGAAGAACGCAGCGAAATGCGATAAGTAATGTGAATTGCAGAATTCAGTGAAT
CATCGAATCTTTGAACGCACCTTGCGCCCCTTGGTATTCCGAGGGGCATGCCTGTTTGAGTGTCATT
AAATTCTCAAACTCACTTTGGTTTCTTTCCAATTGTGATGTTTGGATTGTTGGGGGCTGCTGGCCTTG
AACCAGGTCGGCTCCTCTTAAATGCATTAGCAGGACTTCTCATTGCCTCTGCGCATGATGTGATAATT
ATCACTCATCAATAGCACGCATGAATAGAGTCCAGCTCTCTAATCGTCCGCAAGGACAATTTGACAAT
TTGACCTCAAATCAGGTAGGACTACCCGCTGAACTTAAGCATATCAATAAGCGGAGGAAAAGAAACT
AACAAGGATTCCCCTAGTAACTGCGAGTGAAGCGGGAAAAGCTCAAATTTAAAATCTGGTGGTCTTT
GGCCATCCGAGTTGTAATCTAGAGAAGTGTTATCCGCGCTGGACCGTGTACAAGTCCCCTGGAATG
GGGCGTCATAGAGGGTGAGAATCCCGTCTTTGACACGGACTACCAGGG

Taxonomic assignment was performed using the UNITE 10.0 database, which identified the sequence as the following organism:

Original source

GenBank: PP594197 | ENA: PP594197

Organism

Pleurotus ostreatus

Lineage

Eukaryota; Fungi; Dikarya; Basidiomycota; Agaricomycotina; Agaricomycetes; Agaricomycetidae; Agaricales; Pleurotineae; Pleurotaceae; Pleurotus

> *Sporothrix fusiformis* Length: 604bp

CCGTTGGTGAACCAGCGGAGGGATCATTACAGAGTTTTCTAAACTCCCAACCCTTGCGAACCATACC
CATACTGTTCTCGTTGCTTCCGGCGGGGGACGGGTGGGGGGCTGCCCTCGGGCTGTCCCTCCCCT
CCCGCGCCGGGGGCGGCGGGCCCTACGAACCTTTACATCTCAACCACAAGAAAACCGTCTGAGTCT
AAAAACAAATAATCAAAACTTTCAACAACGGATCTCTTGGCTCTGGCATCGATGAAGAACGCAGCGAA
ATGCGATACGTAATGTGAATTGCAGAATTCAGCGAACCATCGAATCTTTGAACGCACATTGCGCCCG
CCAGTATTCTGGCGGGCATGCCTGTCCGAGCGTCATTTCCCCCCTCACGCGCCCCGTTGCGCGCTG
GTGTTGGGGCTCCTCCGCCTGGCGGAGGGCCCCTAAAGCGAGTGGCGGGCCCGTCTGGCGGCTC
CGAGCGCAGTACCGAACGCAAGTTCTCCTCTCGCCCGGACGCCCCGTCGGCGCCTTGCCAGCCCA
AATCGCGCATGACGTGCAACTCTTTTTTACAAGGTTGACCTCGGATCAGGTAGGACTACCCGCTGAA
CTTAAGCAT

Taxonomic assignment was performed using the UNITE 10.0 database, which identified the sequence as the following organism:

Original source

GenBank: PV350337 | ENA: PV350337

Organism

Sporothrix fusiformis

Lineage

Eukaryota; Fungi; Dikarya; Ascomycota; Pezizomycotina; Sordariomycetes; Sordariomycetidae; Ophiostomatales; Ophiostomataceae; Sporothrix

>*Ophiostoma piceae* CMW 25034 Length: 817bp

TCATTAGCGAGTTTTCAACTCCCAACCCTTGCGAACCGTACCCCGTTCTGTTCTCGTTGCTTCTGGC
GGGAGGGGGGGGCGCGTCCTTCGGGGCGCGCCTCTCTCTCCCAGGTCCCTTCGGGGCGCCCGC
CAGCGGCCGCGAGCCGCCTGAACTTTTTTTAAACCGTAACGAAACGTCTGAGAAACAAACAAAAACA
GCCAAAACTTTCAACAACGGATCTCTTGGCTCTGGCATCGATGAAGAACGCAGCGAAATGCGATACG
TAATGCGAATTGCAGAATTCAGCGAGTCATCGAATCTTTGAACGCACATTGCGCCCGCCAGCATTCT
GGCGGGCATGCCTGTCCGAGCGTCATTTCCCCCCTCAGCACGCCCTTTGGGTGCGCTGGCGTTGG
GGCTCCTCCGCCCTCTGTGGCGGCAGGGCCCTCAAATCCAGTGGCGGGCCCGTCTGGTTGGCTCC
GAGCGCAGTACCGAACGCAAGTTCTCTCTCTCGCTCTGCAGCCCCGGCCGGCGCCCAGCCGTCAA
GCCGCGCAGGCGACCCTCTCGAGGGCCGCCTCGCATTTTTACAAGGTTGACCTCGGATCAGGTAGG
ATTACCCGCTGAACTTAAGCATATCAATAAGCGGAGGAAAAGAAACCAACAGGGATTGCCCCAGTAA
CGGCGAGTGAAGCGGCAACAGCTCAAATTTGAAATCTGGCCCTCGGGTCCGAGTTGTAATTTGGAG
AGGATGCTTTTGGCGCGGCGCCGTCCGAGTTCCTTGGAACAGGACGCCATAGAGGGTGAGAGCCC
CGTACGGGCGGCCGCCTAGCCTTTGT

Taxonomic assignment was performed using the UNITE 10.0 database, which identified the sequence as the following organism:

Original source

GenBank: AM944646 | ENA: AM944646

Organism

Ophiostoma piceae

Lineage

Eukaryota; Fungi; Dikarya; Ascomycota; Pezizomycotina; Sordariomycetes; Sordariomycetidae; Ophiostomatales; Ophiostomataceae; Ophiostoma

>*Nectria_pseudotrichia* UF-PT101 Length: 1261bp

CACACCGCCCGTCGCTACTACCGATTGAATGGCTCAGTGAGGCGTCCGGACTGGCCCAGAGAGGT
GGGCAACTACCACTCAGGGCCGGAAAGCTCTCCAAACTCGGTCATTTAGAGGAAGTAAAAGTCGTAA
CAAGGTCTCCGTTGGTGAACCAGCGGAGGGATCATTACCGAGTTTACAACTCCCAAACCCCTGTGAA
CATACCTATAATTGTTGCTTCGGCGGGACCGCCCCGGCGCCCAACTCCGGGCCCGGACCCAGGCG
CCCGCCGGAGGCCCCAAACTCTTTGTTTTTACGTTGTGTATCTTCTGAGTTAAACAAGCAAATAAATC
AAAACTTTCAACAACGGATCTCTTGGTTCTGGCATCGATGAAGAACGCAGCGAAATGCGATAAGTAA
TGTGAATTGCAGAATTCAGTGAATCATCGAATCTTTGAACGCACATTGCGCCCGCTAGTATTCTAGCG
GGCATGCCTGTTCGAGCGTCATTTCAACCCTCAAGCCCCCGTGGCTTGGTGTTGGGGATCGGCCGC
AAGCCTTCACGGCAGGCGGCCGGCCCCGAAATCTAGTGGCGGTCTCGCTGTAGTCCCCCTCTGCG
TAGTAATACACCTCGCACTGGAAGCTCGGCCTGACCACGCCGTAAAACCCCCCACTTCTGAAGGTTG
ACCTCGGATCAGGTAGGAATACCCGCTGAACTTAAGCATATCAATAAGCGGAGGAAAAGAAACCAAC
AGGGATTGCCCTAGTAACGGCGAGTGAAGCGGCAACAGCTCAAATTTGAAATCTGGCCCTCGTGGT
CCGAGTTGTAATTTGTAGAGGATGCTTTTGGTTAGGTGCCTTCCGAGTTCCCTGGAACGGGACGCCA
TAGAGGGTGAGAGCCCCGTCTGGTTGGACACCGAGCCTCTGTAAAGCTCCTTCGACGAGTCGAGTA
GTTTGGGAATGCTGCTCTAAATGGGAGGTATATGTCTTCTAAAGCTAAATATTGGCCAGAGACCGATA
GCGCACAAGTAGAGTGATCGAAAGATGAAAAGCACTTTGAAAAGAGGGTTAAAAAGTACGTGAAATT
GTTGAAAGGGAAGCGCTTGTGACCAGACTTGGGCCCGGTCAATCATCCAGGGTTCTCCCTGGTGCA
CTTGGCCGGCCCAGGCCAGCATCAGTTTGTCGCGGGGGATAAAGACGTTGGGAATGTGGCTCCTCC
GGGAGTGTTATAGCCCTTCGTGTAATACCCTGCTTCGGACTGAGGTTCGCGCATCTGCAAGGA

Taxonomic assignment was performed using the UNITE 10.0 database, which identified the sequence as the following organism:

Original source

GenBank: HM534899 | ENA: HM534899

Organism

Nectria pseudotrichia

Lineage

Eukaryota; Fungi; Dikarya; Ascomycota; Pezizomycotina; Sordariomycetes; Hypocreomycetidae; Hypocreales; Nectriaceae; Nectria

>*Heterobasidion annosum* P402 Length: 859bp

TCCGTAGGTGAACCTGCGGAAGGATCATTAACGAATATCGTGCAAGGTTGAAGCTGGCCTCTCGGG
GCATGTGCTCGCCTTGTTCATATCCATCTCACACCTGTGCACACTCGCGTGGGTCGGTCGGGTTCTT
TTGACCCCTTCCGAGCCACGTCTTCTCACAAACTCTTCGTATGTCTTTAGAATGGTATCAATGCTATA
AAACGCATCTTATACAACTTTCAACAATGGATCTCTCGGTTCTCGCATCGATGAAGAACGCAGCGAAA
TGCGATAAGTAATGTGAATTGCAGAATTCAGTGAATCATCGAATCTTTGAACGCACCTTGCGCCCTTT
GGTATTCCGAAGGGCACGCCTGTTTGAGTGTCGTGAAATTCTCAACCCTGTGCTTTTCTTGTGAAAG
CGCGTGGGCTTGGACTTGGAGGCTTTGCTGGTCCTCGCGGATCGGCTCCTCTCAAATGCATTAGCG
AGACCCTTGTGGTGCCGCCCCCGGTGTGATAATTGTCTACGCCGTGGTGGTACGCCACGATTGTGG
GGGACCTGCTTCCAACCGTCGAAAGACAAACTTTATCGAAACTTGACCTCAAATCAGGCGGGACTAC
CCGCTGAACTTAAGCATATCAATAAGCGGAGGAAAAGAAACTAACAAGGATTCCCCTAGTAACTGCG
AGTGAAGCGGGAAAAGCTCAAATTTAAAATCTGGCGGCCTCCGGTCGTCCGAGTTGTAGTCTGGAG
AAGTGTTTTCCGCGTCGGACCGTGTATAAGTTTCCTGGAACGGAGCGTCATAGAGGGTGAGAATCC
CGTCTTTGACACGGACTGCCGATGCTTTGTGATGCGCTCTCGAAGAGTCGAGTTGTT

Taxonomic assignment was performed using the UNITE 10.0 database, which identified the sequence as the following organism:

Original source

GenBank: DQ384592 | ENA: DQ384592

Organism

Heterobasidion annosum

Lineage

Eukaryota; Fungi; Dikarya; Basidiomycota; Agaricomycotina; Agaricomycetes; Russulales; Bondarzewiaceae; Heterobasidion; Heterobasidion annosum species complex

>*Endoconidophora polonica* MPICE-2014-1 Length: 760bp

TTCTTCATCGATGCAGAGCCAAGAGATCCGTTGGAAAGTTTTAACTTATTATGTTAATGCCACTCAGC
AATGAAAATAAATTCTCTAGAAATATTAAAGAGTTTTTGTAAATACTACCGGCAGGACTTGTTTTACCA
AGACCTGCCAAAGCAGCTAAAAAAGGTATGTTCACATATGGTTTAAGAGTTAAAAACTCAGTAATGAT
CCCTCCGCAGGTTCACCTACGGAAATATCATTACTGAGTTTTTAACTCTTAAACCATATGTGAACATA
CCTTTTTTAGCTGCTTTGGCAGGTCTTGGTAAAACAAGTCCTGCCGGTAGTATTTACAAAAACTCTTT
AATATTTCTAGAGAATTTATTTTCATTGCTGAGTGGCATTAACATAATAAGTTAAAACTTTCAACAACG
GATCTCTTGGCTCTAGCATCGATGAAGAACGCAGCGAAATGCGATACGTAATGTGAATTGCAGAATT
CAGTGAATCATCGAATCTTTGAACGCACATTGCGCCTGGCAGTATTCTGCCAGGCATGCCTGTCCGA
GCGTCATTTCACCACTCAAGCTCTGCTTGGTGTTGGAGGACCCGCATCTTGCGGGCCGCCGAAATG
CATCGGCTGTTGAATTTGCAGCTTCCCTGTGTAGTAATATTTATTTTACGCTTTGAAACTCTTTTACAA
CATCGCCGTAAAACCCCCCTCAATTATATTTGAAAGGTTGACCTCGGATCAGGTAGGAATACCCGCT
GAACTTAAGCATAT

Taxonomic assignment was performed using the UNITE 10.0 database, which identified the sequence as the following organism:

Original source

GenBank: MW256648 | ENA: MW256648

Organism

Endoconidiophora polonica

Lineage

Eukaryota; Fungi; Dikarya; Ascomycota; Pezizomycotina; Sordariomycetes; Hypocreomycetidae; Microascales; Ceratocystidaceae; Endoconidiophora

> *Coprinellus radians* UF-PT141 Length: 637bp

GAATAACTATGGTGTCTCAGGTTGTAGCTGGCTCCTCGGAGCAATGTGCACGCCCGCCATTTTTATC
TTTCCACCTGTGCACCGACTGTAGGTCTGGATACCTCTCGCCGCAAGGCGGATGCGAGGGTTGCTC
GTAAAGGGCTCCCCTCGAACTTCCAGGCTCTACGTCTTTTTACACACCCCAATAGTATGATGCAGAAT
GTAGTCAATGGGCTTCACAGCCTATAAAACACTATACAACTTTCAGCAACGGATCTCTTGGCTCTCGC
ATCGATGAAGAACGCAGCGAAATGCGATAAGTAATGTGAATTGCAGAATTCAGTGAATCATCGAATCT
TTGAACGCACCTTGCGCTCCTTGGTATTCCGAGGAGCATGCCTGTTTGAGTGTCATTAAATTCTCAAC
CTCACCCGTTTTCTGAACGGGGTCGAGGCTTGGATGTGGGGGTTTGTGCAGGCTGCCTTCACTGGC
GGTCTGCTCCCCTGAAATGCATTAGCGAGGTTCATGCTGAACTTCCGTCTATTGGTGTGATAATTATC
TACGCCGTGGACGAGGATCAGACTCGCTTCTAACCGTCCGCGAGGACAATACCTTGACAATTTGACC
TCAAATCAGGTAGGACTACCCGCTGAACTTA

Taxonomic assignment was performed using the UNITE 10.0 database, which identified the sequence as the following organism:

Original source

GenBank: MK887319 | ENA: MK887319

Organism

Coprinellus radians

Lineage

Eukaryota; Fungi; Dikarya; Basidiomycota; Agaricomycotina; Agaricomycetes; Agaricomycetidae; Agaricales; Psathyrellaceae; Coprinellus

>*Chaetomium globosum* P7-2 Length: 529bp

ATTACAGAGTTGCAAAACTCCCTAAACCATTGTGAACGTTACCTATACCGTTGCTTCGGCGGGCGGC
CCCGGGGTTTACCCCCCGGGCGCCCCTGGGCCCCACCGCGGGCGCCCGCCGGAGGTCACCAAAC
TCTTGATAATTTATGGCCTCTCTGAGTCTTCTGTACTGAATAAGTCAAAACTTTCAACAACGGATCTCT
TGGTTCTGGCATCGATGAAGAACGCAGCGAAATGCGATAAGTAATGTGAATTGCAGAATTCAGTGAA
TCATCGAATCTTTGAACGCACATTGCGCCCGCCAGCATTCTGGCGGGCATGCCTGTTCGAGCGTCAT
TTCAACCATCAAGCCCCCGGGCTTGTGTTGGGGACCTGCGGCTGCCGCAGGCCCTGAAAAGCAGT
GGCGGGCTCGCTGTCGCACCGAGCGTAGTAGCATACATCTCGCTCTGGTCGCGCCGCGGGTTCCG
GCCGTTAAACCACCTTTTAACCCAAGGTTGACCTCGGATCAGGTAGGAAGACCCGCTGAACTTAA

Taxonomic assignment was performed using the UNITE 10.0 database, which identified the sequence as the following organism:

Original source

GenBank: PV453791 **|** ENA: PV453791

Organism

Chaetomium globosum

Lineage

Eukaryota; Fungi; Dikarya; Ascomycota; Pezizomycotina; Sordariomycetes; Sordariomycetidae; Sordariales; Chaetomiaceae; Chaetomium

>*Biscogniauxia_nummularia* UF-PT69 Length: 1022bp

AGGTCTCCGTTGGTGAACCAGCGGAGGGATCATTAGCGAGTTAAACAAACTCCAAACCCATGTGAAC
ATACCTACTGTTGCCTCGGCAGGTCGTGCTGTGCGGTGGTGATACCTACCCTGGAGTAGGAACCTA
CCCTGTAGTGACCTACCCTGGAGTGGAGCTACCCTGCAGCACGCAACCAGGCCTGCCAAAGGACCC
CTAAACTCTGTTATAACGTACCTCTGAGTCAACTATACAAATAAGTTAAAACTTTCAACAACGGATCTC
TTGGTTCTGGCATCGATGAAGAACGCAGCGAAATGCGATAAGTAATGTGAATTGCAGAATTCAGTGA
ATCATCGAATCTTTGAACGCACATTGCGCCTAATAGTATTCTGTTAGGCATGCCTGTTCGAGCGTCAT
TTCAACCCCCAAGCGCTATTTAGCTTGACGTTGGGAATTTACAGCTGCTGTAATTCCTCAAATTTAGT
GGCGGAGCTAGGTCATGCTCTGAGCGTAGTAATTCTGTTCTCGCTCCTGAAGCTGCCCTATATCCTG
CCGTAAAACCCCCTAAAATTATAATCTGGTTGACCTCGGATCAGGTAGGAATACCCGCTGAACTTAA
GCATATCAATAAGCGGAGGAAAAGAAACCAACAGGGATTGCCCTAGTAACGGCGAGTGAAGCGGCA
ACAGCTCAAATTTGAAATCTGGCCTTCGGGTCCGAGTTGTAATTTGCAGAGGATGCTTTTGGCGAGG
TGCCTTCCGAGTTCCCTGGAACGGGACGCCTTAGAGGGTGAGAGCCCCGTACGGTTGGACACCGA
GCCTCTGTAAAGCTCCTTCGACGAGTCGAGTAGTTTGGGAATGCTGCTCTAAATGGGAGGTAAATTT
CTTCTAAAGCTAAATACCGGCCAGAGACCGATAGCGCACAAGTAGAGTGATCGAAAGATGAAAAGCA
CTTTGAAAAGAGGGTTAAATAGCACGTGAAATTGTTGAAAGGGAAGCGTTTACGGCCAGACCTTCTC
CTGGCGGATCATCCGGTG

Taxonomic assignment was performed using the UNITE 10.0 database, which identified the sequence as the following organism:

Original source

GenBank: LN714525 | ENA: LN714525

Organism

Biscogniauxia nummularia

Lineage

Eukaryota; Fungi; Dikarya; Ascomycota; Pezizomycotina; Sordariomycetes; Xylariomycetidae; Xylariales; Xylariaceae; Biscogniauxia

>*Cylindrobasidium ipidophilum* CBS_116786 Length: 776bp

AAGGTTTCCGTAGGTGAACCTGCGGAAGGATCATTAATGAAATAATACTTTGCAGCTTCAAGTTGTTG
CTGGTCTCTAAAAAGAGACATGTGCACACTTGTTGTTGTGAATATTCATCCACCTGTGCACTTCTGTA
AGTCTGGATAACTATCACAAAATTTATTTTGTGGTTTGAAAGGTTGCTTTACAAGCTCCTTTTGACTTT
CCAGACTTATGAATTCATATACTCTTGTATGTCTTGAATGTCTTGTTTACTGGACGTATGTCCTTTAAA
ATTATATACAACTTTCAACAACGGATCTCTTGGCTCTCGCATCGATGAAGAACGCAGCGAAATGCGAT
AACTAATGTGAATTGCAGAATTCAGTGAATCATCGAGTCTTTGAACGCACCTTGCGCCCTCTGGTATT
CCGGAGGGCATGCCTGTTTGAGTGTCAGTAAATTCTCAACCCTCTCATGCTTTGTTGGCATTGAGTG
GATTGGATATGGAGGTTTGCTGGATTTTATTCAGCTCCTCTAAAATATATTAGCAGAAACCATTGCTAA
GGTTTACTGCAGCCGTGTGATAATTATCTTACGGTTGAAGTAAATGTTTAGTTTGTCTCACTTTTGGTT
TGAAGAGATGCTCTTTGAGCTCCCTTTGCTTTTCTCTCTTTGGAGTGATACTTATCCAAGTGTAGATTT
TAATGGGTCTTATTCAGCTTCTAATCATCTGGAAACAGATAATACTTGACCATTTGGCCTCAAATCAG
GTAGGACTACCCGCTGAACTTAAG

Taxonomic assignment was performed using the UNITE 10.0 database, which identified the sequence as the following organism:

Original source

GenBank: MZ297304 | ENA: MZ297304

Organism

Cylindrobasidium ipidophilum

Lineage

Eukaryota; Fungi; Dikarya; Basidiomycota; Agaricomycotina; Agaricomycetes; Agaricomycetidae; Agaricales; Marasmiineae; Physalacriaceae; Cylindrobasidium

>*Cylindrobasidium ipidophilum* CBS _120295 Length: 780bp

TATGCTTAAGTTCAGCGGGTAGTCCTACCTGATTTGAGGCCAAATGGTCAAGTATTATCTGTTTCCAG
ATGATTAGAAGCTGAATAAGACCCATTAAAATCTACACTTGGATAAGTATCACTCCAAAGAGAGAAAA
GCAAAGGGAGCTCAAAGAGCATCTCTTCAAACCAAAAGTGAGACAAACTAAACATTTACTTCAACCGT
AAGATAATTATCACACGGCTGCAGTAAACCTTAGCAATGGTTTCTGCTAATATATTTTAGAGGAGCTG
AATAAAATCCAGCAAACCTCCATATCCAATCCACTCAATGCCAACAAAGCATGAGAGGGTTGAGAATT
TACTGACACTCAAACAGGCATGCCCTCCGGAATACCAGAGGGCGCAAGGTGCGTTCAAAGACTCGA
TGATTCACTGAATTCTGCAATTCACATTAGTTATCGCATTTCGCTGCGTTCTTCATCGATGCGAGAGC
CAAGAGATCCGTTGTTGAAAGTTGTATATAATTTTAAAGGACATACGTCCAGTAAACAAGACATTCAA
GACATACAAGAGTATATGAATTCATAAGTCTGGAAAGTCAAAAGGAGCTTGTAAAGCAACCTTTCAAA
CCACAAAATAAATTTTGTGATAGTTATCCAGACTTACAGAAGTGCACAGGTGGATGAATATTCACAAC
AACAAGTGTGCACATGTCTCTTTTTAGAGACCAGCAACAACTTGAAGCTGCAAAGTATTATTTCATTAA
TGATCCTTCCGCAGGTTCACCTACGGAAACCTT

Taxonomic assignment was performed using the UNITE 10.0 database, which identified the sequence as the following organism:

Original source

GenBank: MZ297304 | ENA: MZ297304

Organism

Cylindrobasidium ipidophilum

Lineage

Eukaryota; Fungi; Dikarya; Basidiomycota; Agaricomycotina; Agaricomycetes; Agaricomycetidae; Agaricales; Marasmiineae; Physalacriaceae; Cylindrobasidium

>*Cylindrobasidium ipidophilum* IFFF-BOKU KF/28/3 Length: 746bp

TTGAGGCCAATGGTCAAGTATTATCTGTTTCCAGATGATTAGAAGCTGAATAAGACCCATTAAAATCT
ACACTTGGATAAGTATCACTCCAAAGAGAGAAAAGCAAAGGGAGCTCAAAGAGCATCTCTTCAAACC
AAAAGTGAGACAAACTAAACATTTACTTCAACCGTAAGATAATTATCACACGGCTGCAGTAAACCTTA
GCAATGGTTTCTGCTAATATATTTTAGAGGAGCTGAATAAAATCCAGCAAACCTCCATATCCAATCCA
CTCAATGCCAACAAAGCATGAGAGGGTTGAGAATTTACTGACACTCAAACAGGCATGCCCTCCGGAA
TACCAGAGGGCGCAAGGTGCGTTCAAAGACTCGATGATTCACTGAATTCTGCAATTCACATTAGTTAT
CGCATTTCGCTGCGTTCTTCATCGATGCGAGAGCCAAGAGATCCGTTGTTGAAAGTTGTATATAATTT
TAAAGGACATACGTCCAGTAAACAAGACATTCAAGACATACAAGAGTATATGAATTCATAAGTCTGGA
AAGTCAAAAGGAGCTTGTAAAGCAACCTTTCAAACCACAAAATAAATTTTGTGATAGTTATCCAGACTT
ACAGAAGTGCACAGGTGGATGAATATTCACAACAACAAGTGTGCACATGTCTCTTTTTAGAGACCAG
CAACAACTTGAAGCTGCAAAGTATTATTTCATTAATGATCCTTCCGCAGGTTCACCTACGGAAACCTT

Taxonomic assignment was performed using the UNITE 10.0 database, which identified the sequence as the following organism:

Original source

GenBank: MZ297304 | ENA: MZ297304

Organism

Cylindrobasidium ipidophilum

Lineage

Eukaryota; Fungi; Dikarya; Basidiomycota; Agaricomycotina; Agaricomycetes; Agaricomycetidae; Agaricales; Marasmiineae; Physalacriaceae; Cylindrobasidium

>*Cylindrobasidium ipidophilum* CBS_119238 Length: 781bp

AAGGTTTCCGTAGGTGAACCTGCGGAAGGATCATTAATGAAATAATACTTTGCAGCTTCAAGTTGTTG
CTGGTCTCTAAAAAGAGACATGTGCACACTTGTTGTTGTGAATATTCATCCACCTGTGCACTTCTGTA
AGTCTGGATAACTATCACAAAATTTATTTTGTGGTTTGAAAGGTTGCTTTACAAGCTCCTTTTGACTTT
CCAGACTTATGAATTCATATACTCTTGTATGTCTTGAATGTCTTGTTTACTGGACGTATGTCCTTTAAA
ATTATATACAACTTTCAACAACGGATCTCTTGGCTCTCGCATCGATGAAGAACGCAGCGAAATGCGAT
AACTAATGTGAATTGCAGAATTCAGTGAATCATCGAGTCTTTGAACGCACCTTGCGCCCTCTGGTATT
CCGGAGGGCATGCCTGTTTGAGTGTCAGTAAATTCTCAACCCTCTCATGCTTTGTTGGCATTGAGTG
GATTGGATATGGAGGTTTGCTGGATTTTATTCAGCTCCTCTAAAATATATTAGCAGAAACCATTGCTAA
GGTTTACTGCAGCCGTGTGATAATTATCTTACGGTTGAAGTAAATGTTTAGTTTGTCTCACTTTTGGTT
TGAAGAGATGCTCTTTGAGCTCCCTTTGCTTTTCTCTCTTTGGAGTGATACTTATCCAAGTGTAGATTT
TAATGGGTCTTATTCAGCTTCTAATCATCTGGAAACAGATAATACTTGACCATTTGGCCTCAAATCAG
GTAGGACTACCCGCTGAACTTAAGCATAT

Taxonomic assignment was performed using the UNITE 10.0 database, which identified the sequence as the following organism:

Original source

GenBank: MZ297304 | ENA: MZ297304

Organism

Cylindrobasidium ipidophilum

Lineage

Eukaryota; Fungi; Dikarya; Basidiomycota; Agaricomycotina; Agaricomycetes; Agaricomycetidae; Agaricales; Marasmiineae; Physalacriaceae; Cylindrobasidium

>*Formitopsis pinicola* Length: 788bp

CTGCGGAAGGATCATTAATGAATTCTGAAAGGGGTTGTAGCTGGCTGTTTCGTTTGAGCGGCATGTG
CACGCCCTGATCATTATCCATCTCACACACCTGTGCACATACTGTAGGTCGGCTTTTGATTGGAGTG
GGGTCTTCATCGACTCTGCTTTTTAGTTGGGGCCTTCCTATGTTTTATCACACACTACTTCAGTTTAAA
GAATGTCCTCTTGCGTCTAACGCATTTAAATACAACTTTCAGCAACGGATCTCTTGGCTCTCGCATCG
ATGAAGAACGCAGCGAAATGCGATAAGTAATGTGAATTGCAGAATTCAGTGAATCATCGAATCTTTGA
ACGCACCTTGCGCTCCTTGGTATTCCGAGGAGCATGCCTGTTTGAGTGTCATGGAATTCTCAACTCT
ATTTGCTTTTGTGAATAGCAGCTTGGACTTGGAGGTTTATTGCCGGTACCTGTAGATCGGCTCCTCTT
GAATGCATTAGCTCGAACCTTTTGTGGATCAGCTTATCGGTGTGATAAAATGTCTACGCCGTTACTGT
GAAGCATATTATTCGGCTTCCAATCGTCCTTCACGGGACAATAACTTTGACCTTTGACCTCAAATCAG
GTAGGATTACCCGCTGAACTTAAGCATATCAATAAGCGGAGGAAAAGAAACTAACAAGGATTCCCCT
AGTAACTGCGAGTGAAGCGGGAAAAGCTCAAATTTAAAATCTGGCGGTCTTTGGCCGTCCGAGTTGT
AGTCTGGAGAAGTGCTTTCCGCGCTGGACCGTGTACAAGTCTCT

Taxonomic assignment was performed using the UNITE 10.0 database, which identified the sequence as the following organism:

Original source

GenBank: FJ608588 | ENA: FJ608588

Organism

Fomitopsis pinicola

Lineage

Eukaryota; Fungi; Dikarya; Basidiomycota; Agaricomycotina; Agaricomycetes; Polyporales; Fomitopsis

> *Grosmannia penicillata* KT896638.1 Length:545bp

GGGATCATTAGCGAGTTCAAGCAACTCCCAACCCGTGCATACATACCGCATTTCTTGCGCCCGTTGC
CTTCCGGCGGGCGGCGCGCGCCTCCAGCTTCCGGCTGGCGGCGCCCGCCCGCCGGAGGACCAGC
GGCCGCACTCTGAACCTCTTCGTATACTGAGCAGTCTGAGAAAAACCATGAATCGTTAAAACTTTCAA
CAACGGATCTCTTGGTTCTGGCATCGATGAAGAACGCAGCGAACTGCGATAAGTAATGTGAATTGCA
GAATTCAGTGAACCATCGAATCTTTGAACGCACATTGCGCCCGCCAGCATTCTGGCGGGCATGCCT
GTCCGAGCGTCATTTCCACCCTCACGCGCCCCTTTAGTGCGTGGTGTTGGGGCATACTGCGGCTGC
CGCGAGGCACCGCAGGCCCTGAAACCCAGTGGCGGGCCGGCAGAAGGCTCCGAGCGCAGTAAGC
ATCACGCCCTCGCTCCGGACGCCCCTGCTGGCAGCTCGCCCTGCGGCGGAAACGCTTGCAGCAAC
CATCTTAAAGGTTGACCT

Taxonomic assignment was performed using the UNITE 10.0 database, which identified the sequence as the following organism:

Original source

GenBank: KT896638 | ENA: KT896638

Organism

Grosmannia penicillata

Lineage

Eukaryota; Fungi; Dikarya; Ascomycota; Pezizomycotina; Sordariomycetes; Sordariomycetidae; Ophiostomatales; Ophiostomataceae; Grosmannia
