# Supplementary material for: Fungi associated with Norway spruce and its bark beetles detoxify spruce defense compounds by conjugation to α-ribose
Source: ISME J. 2026 Sep 4;20(1):wrag230. doi: 10.1093/ismejo/wrag230 (PMC13637821; doi:10.1093/ismejo/wrag230)

Supplementary File 2: Mass spectra of fungal products and phenolic standards.

Ion chromatograms and mass spectra from UHPLC-qTOF-MS in the negative ionization mode.

Piceatannol-11-*O*-α-ribofuranoside

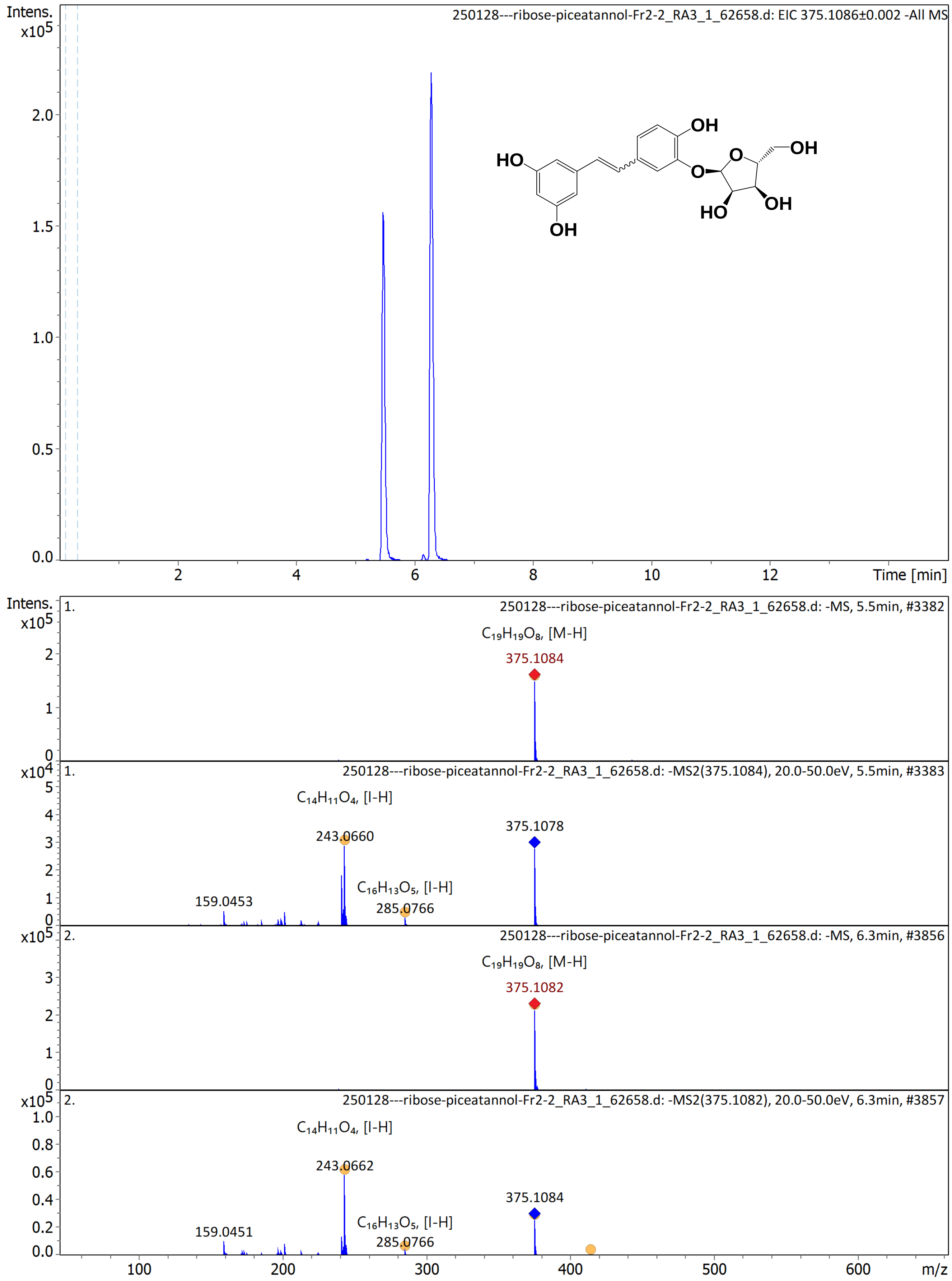

Isorhapontigenin-3-*O*-α-ribofuranoside

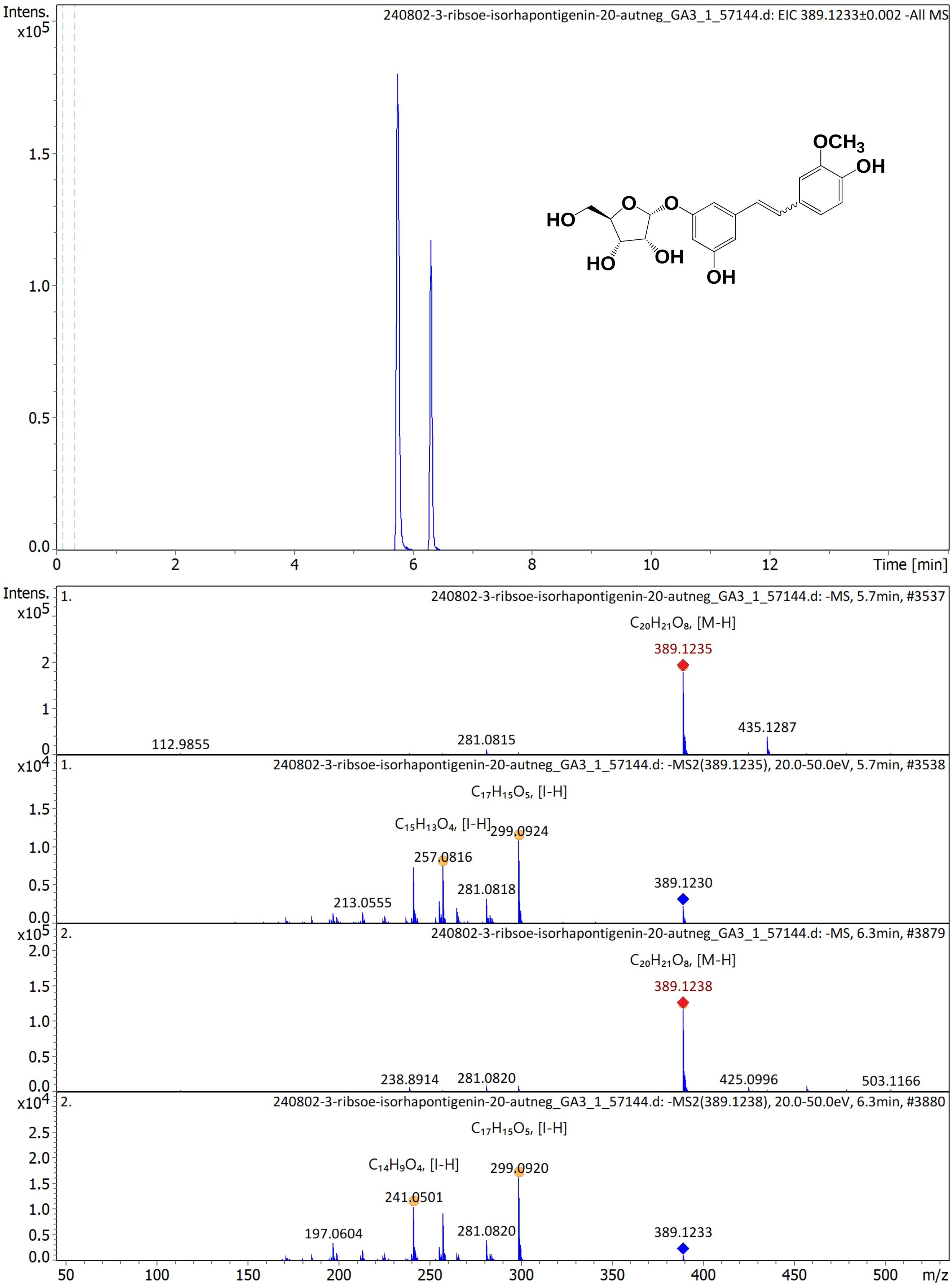

Resveratrol-3-*O*-α-ribofuranoside

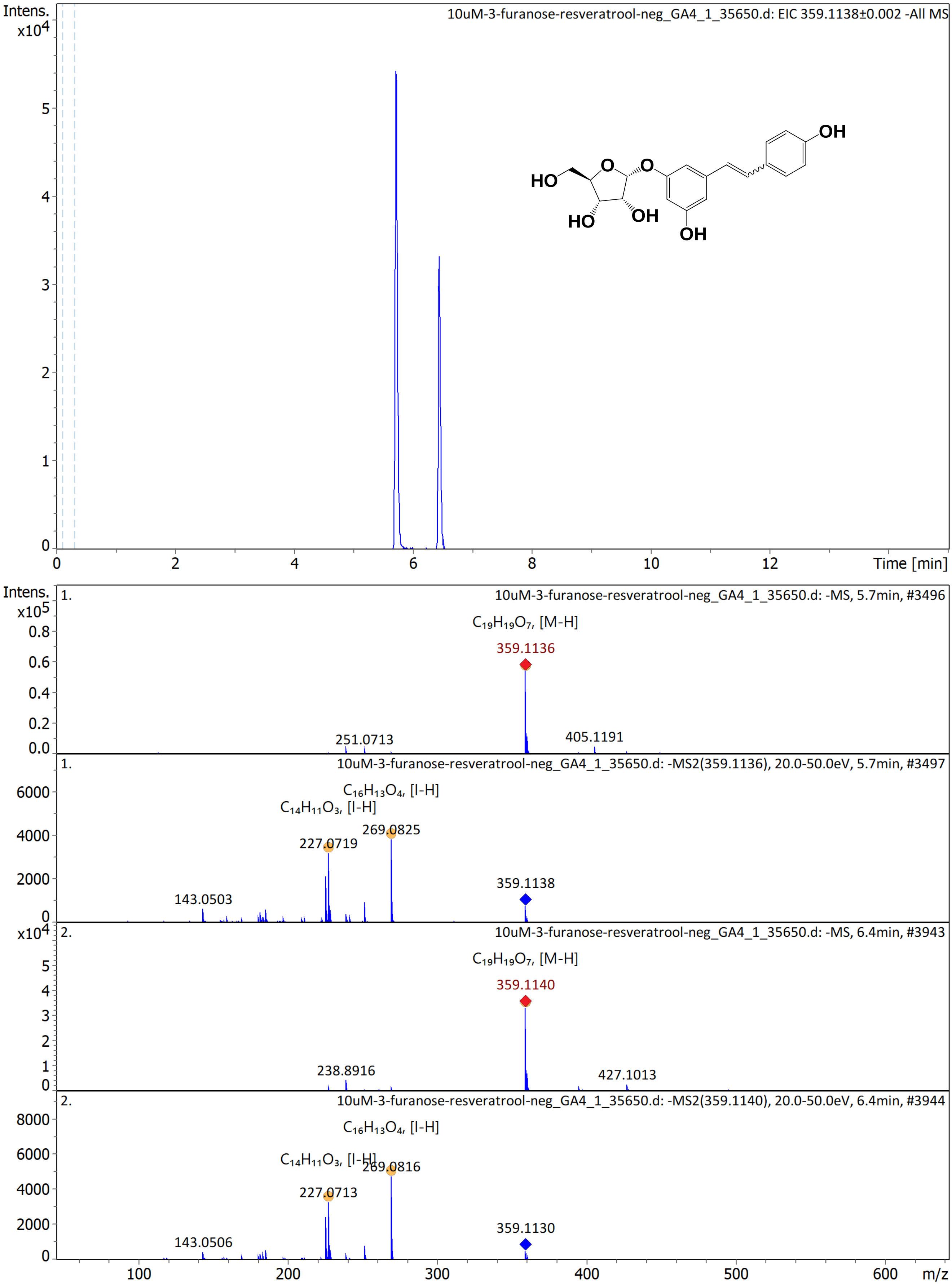

Resveratrol-3,5-*O*-α-ribofuranoside

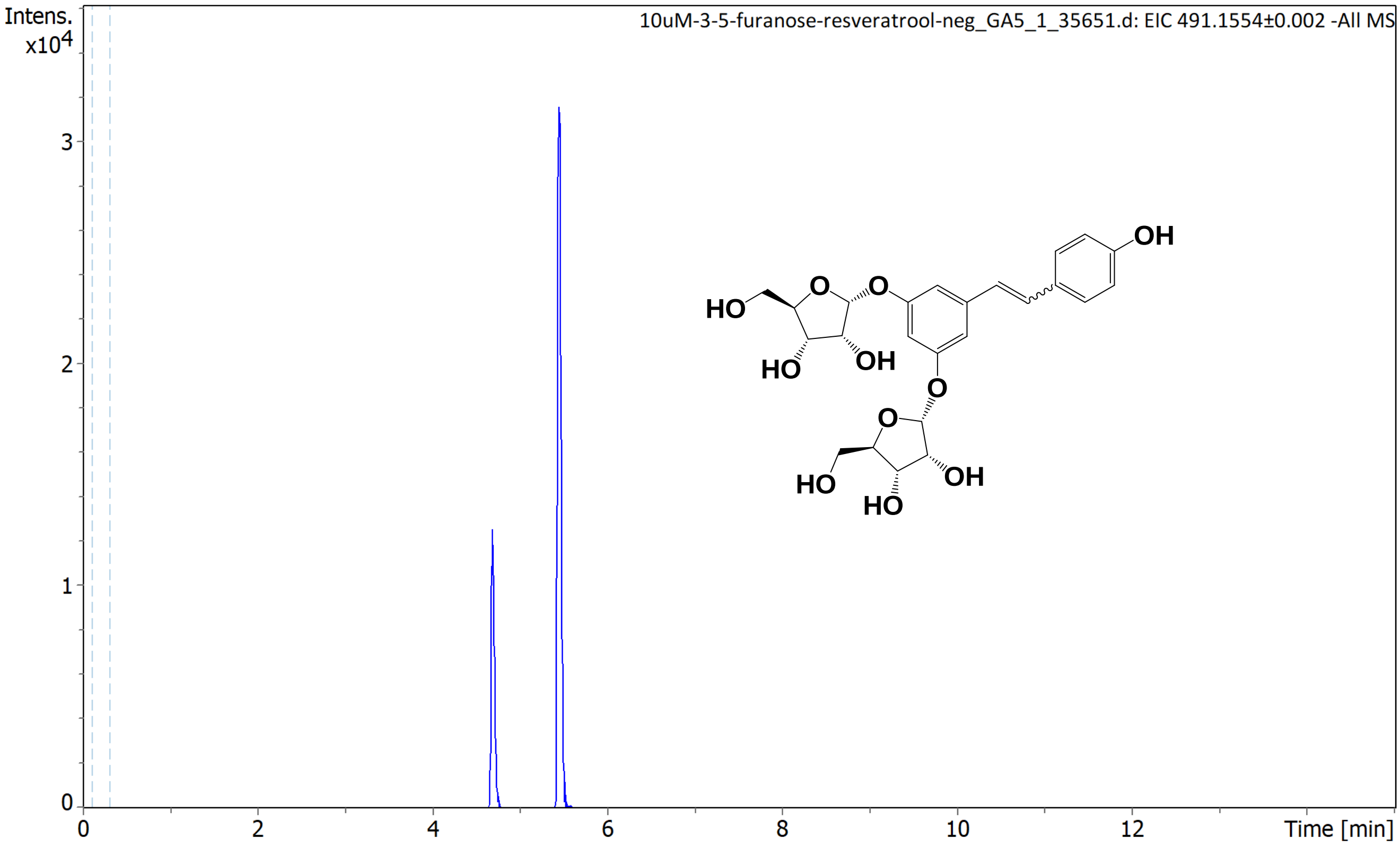

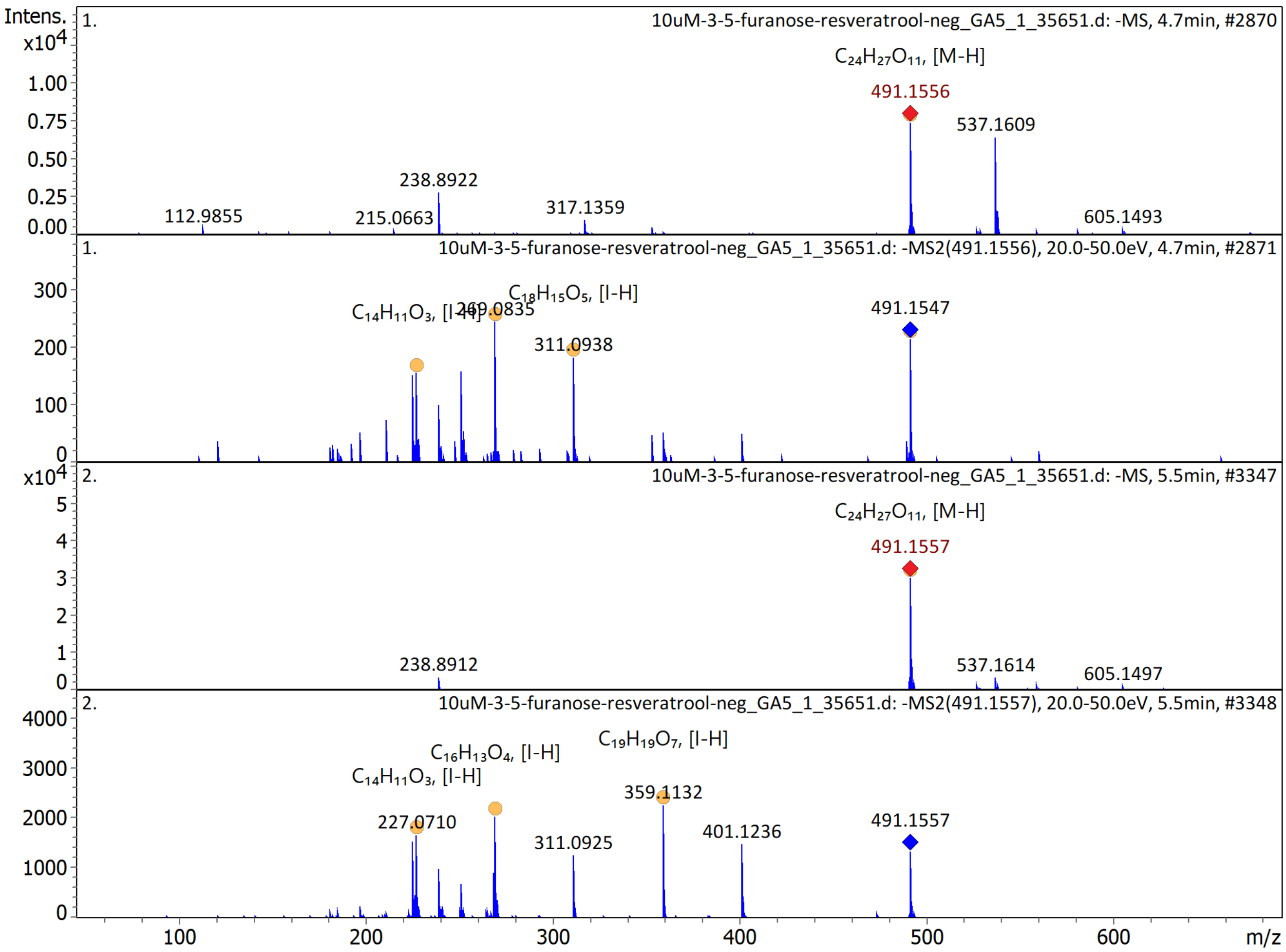

Astringin

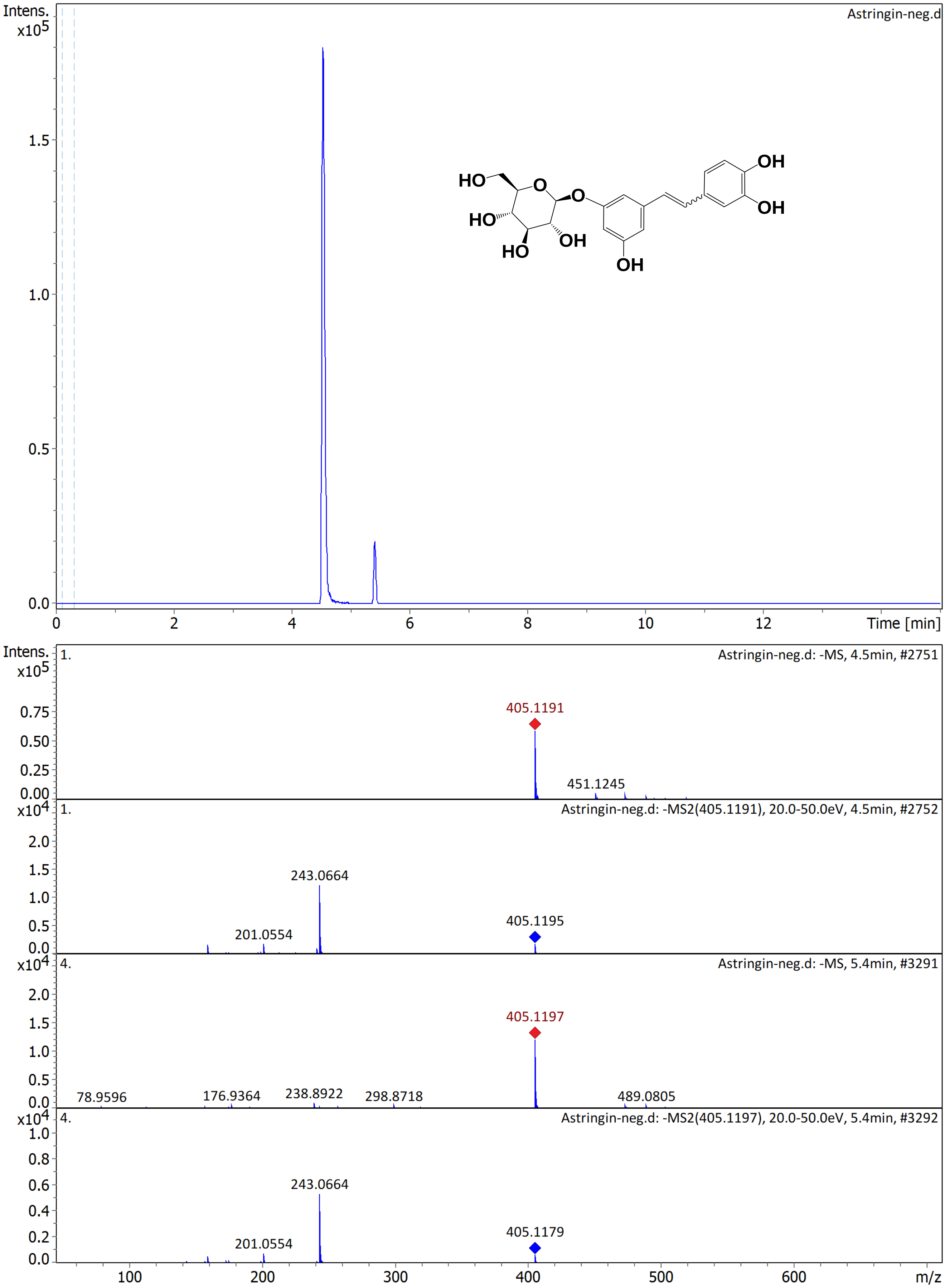

It has been shown in the Sun, 2026 (PNAS)

Piceatannol

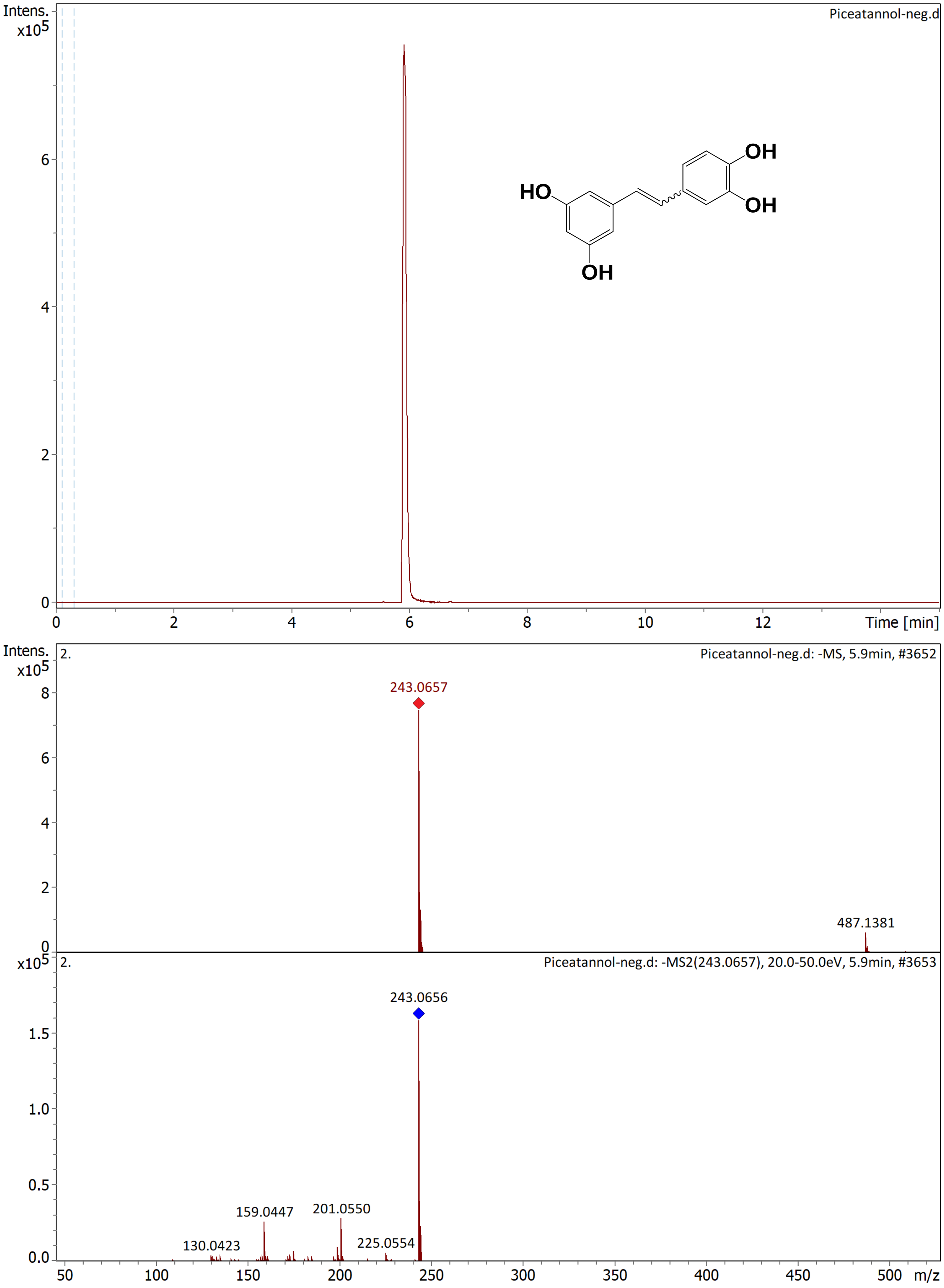

It has been shown in the Sun, 2026 (PNAS)

Longusol C

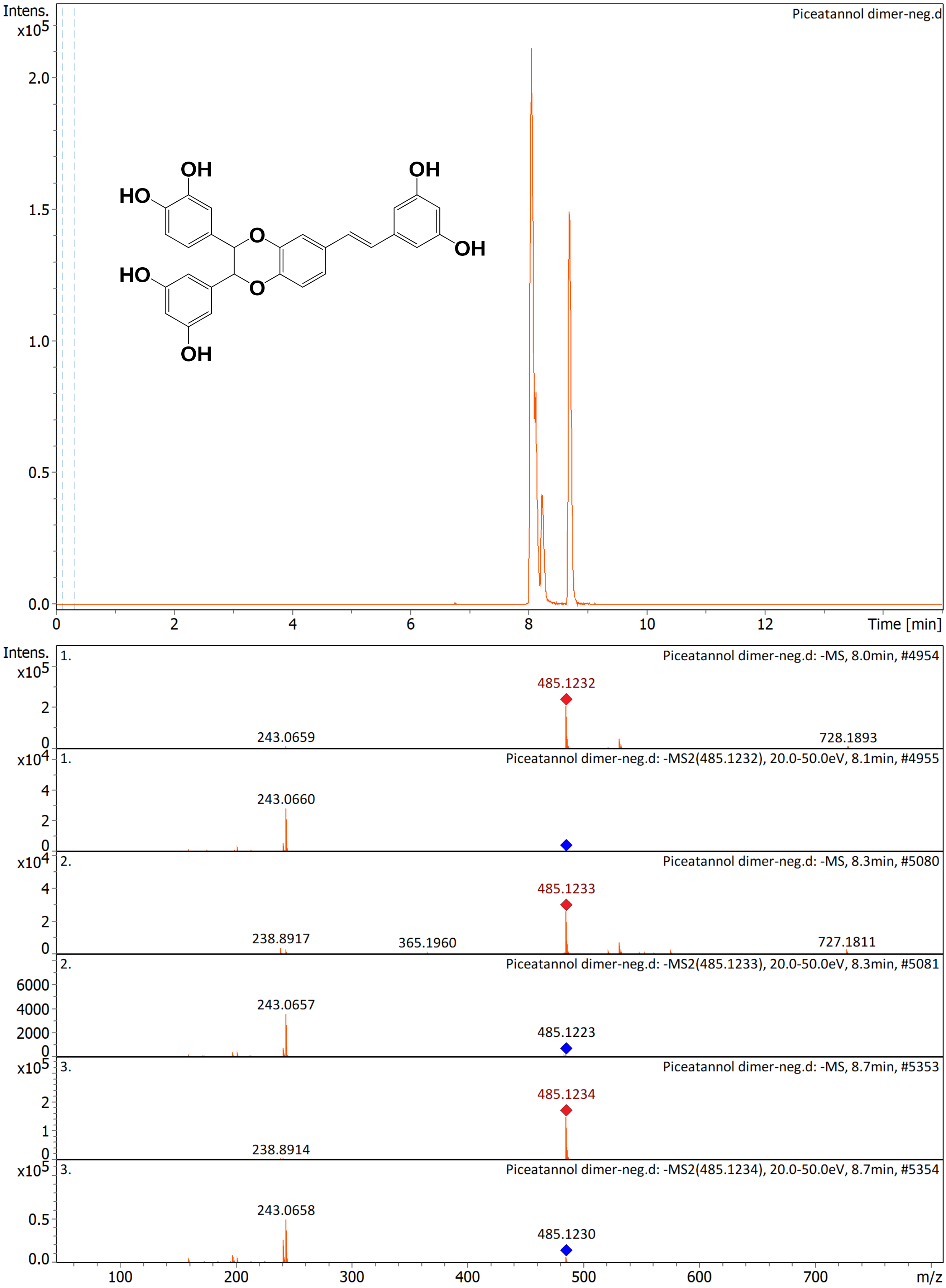
Intens.
x10^5
2.0
1.5
1.0
0.5
0.0
Piceatannol dimer-neg.d
OH
HO
O
O
HO
OH
OH
OH
0
2
4
6
8
10
12
Time [min]
Intens.
x10^5
1.
Piceatannol dimer-neg.d: -MS, 8.0min, #4954
485.1232
243.0659
728.1893
x10^4
1.
Piceatannol dimer-neg.d: -MS2(485.1232), 20.0-50.0eV, 8.1min, #4955
243.0660
x10^4
2.
Piceatannol dimer-neg.d: -MS, 8.3min, #5080
485.1233
238.8917
365.1960
727.1811
2.
Piceatannol dimer-neg.d: -MS2(485.1233), 20.0-50.0eV, 8.3min, #5081
6000
4000
2000
243.0657
485.1223
x10^5
3.
Piceatannol dimer-neg.d: -MS, 8.7min, #5353
485.1234
238.8914
x10^5
3.
Piceatannol dimer-neg.d: -MS2(485.1234), 20.0-50.0eV, 8.7min, #5354
243.0658
485.1230
100
200
300
400
500
600
700
m/z

Isorhapontin

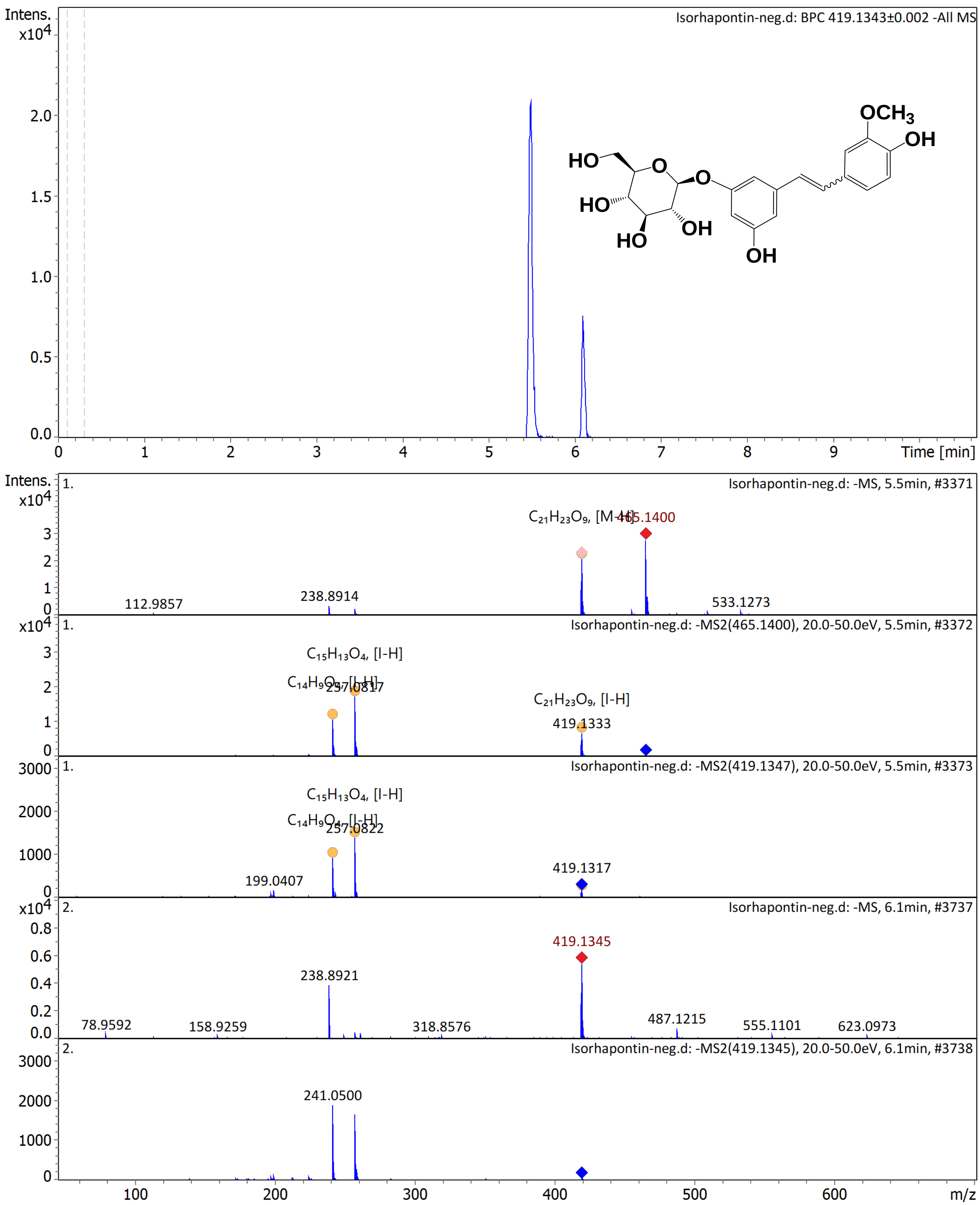

It has been shown in the Sun, 2026 (PNAS)

Isorhapontigenin

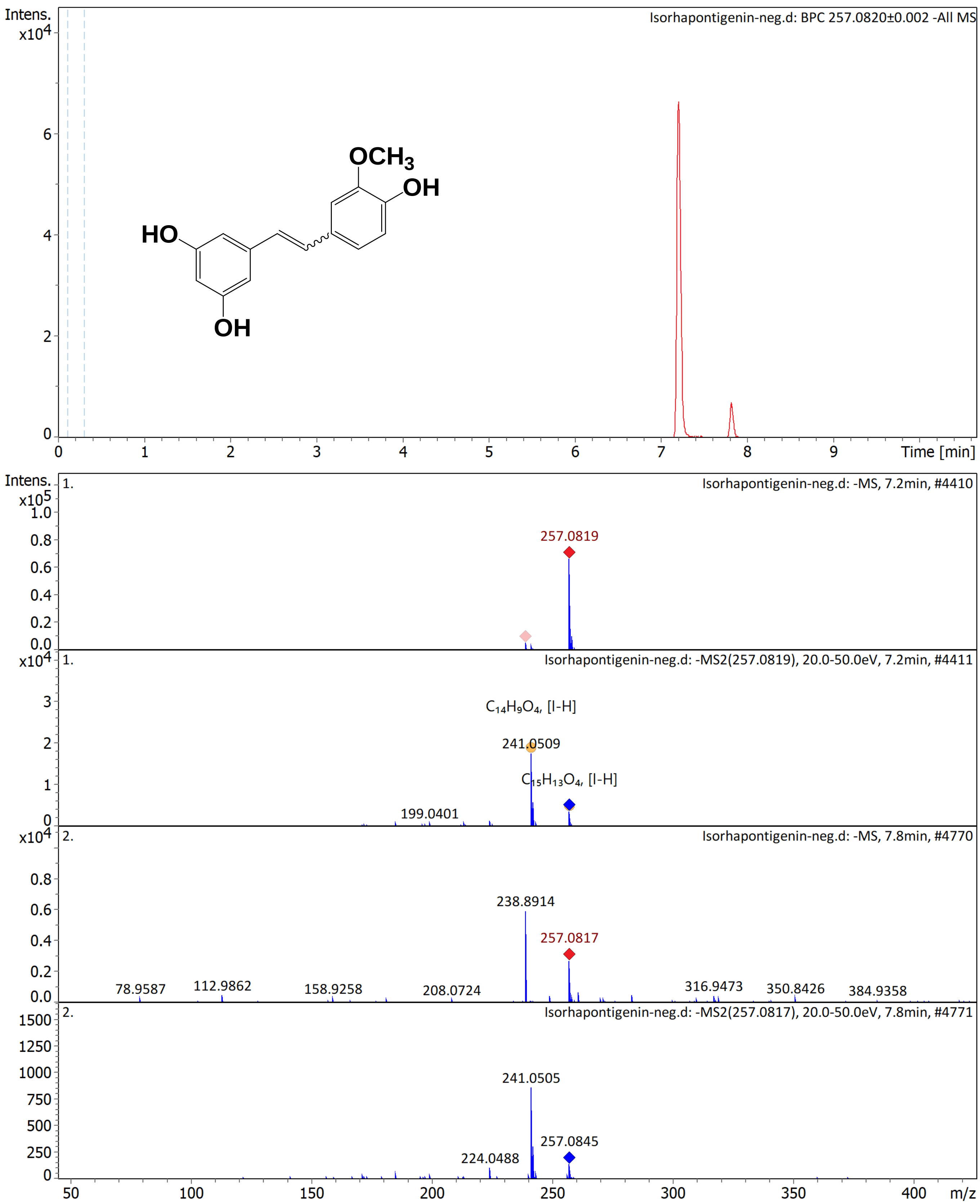

It has been shown in the Sun, 2026 (PNAS)

Piceid

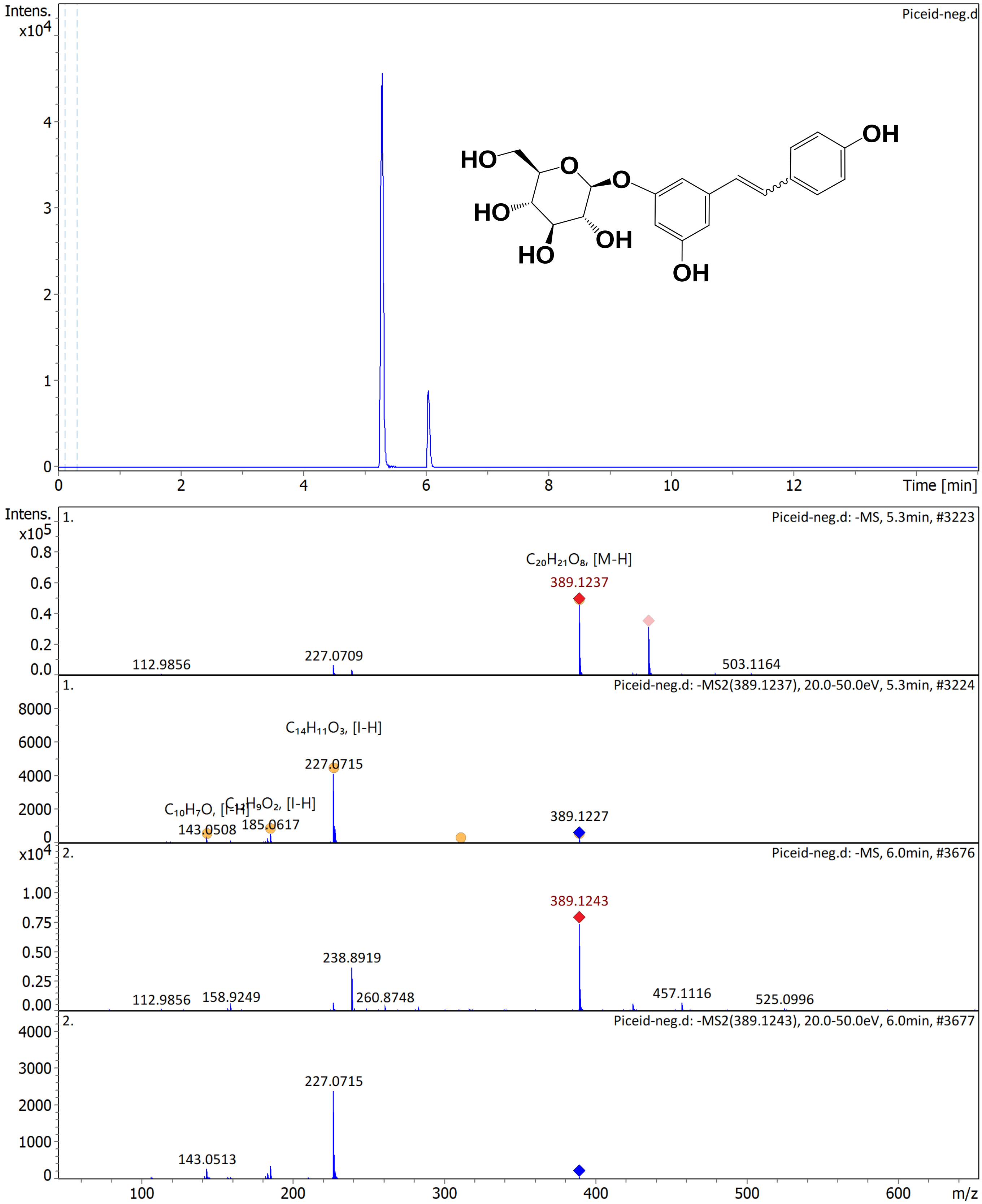

It has been shown in the Sun, 2026 (PNAS)

Resveratrol

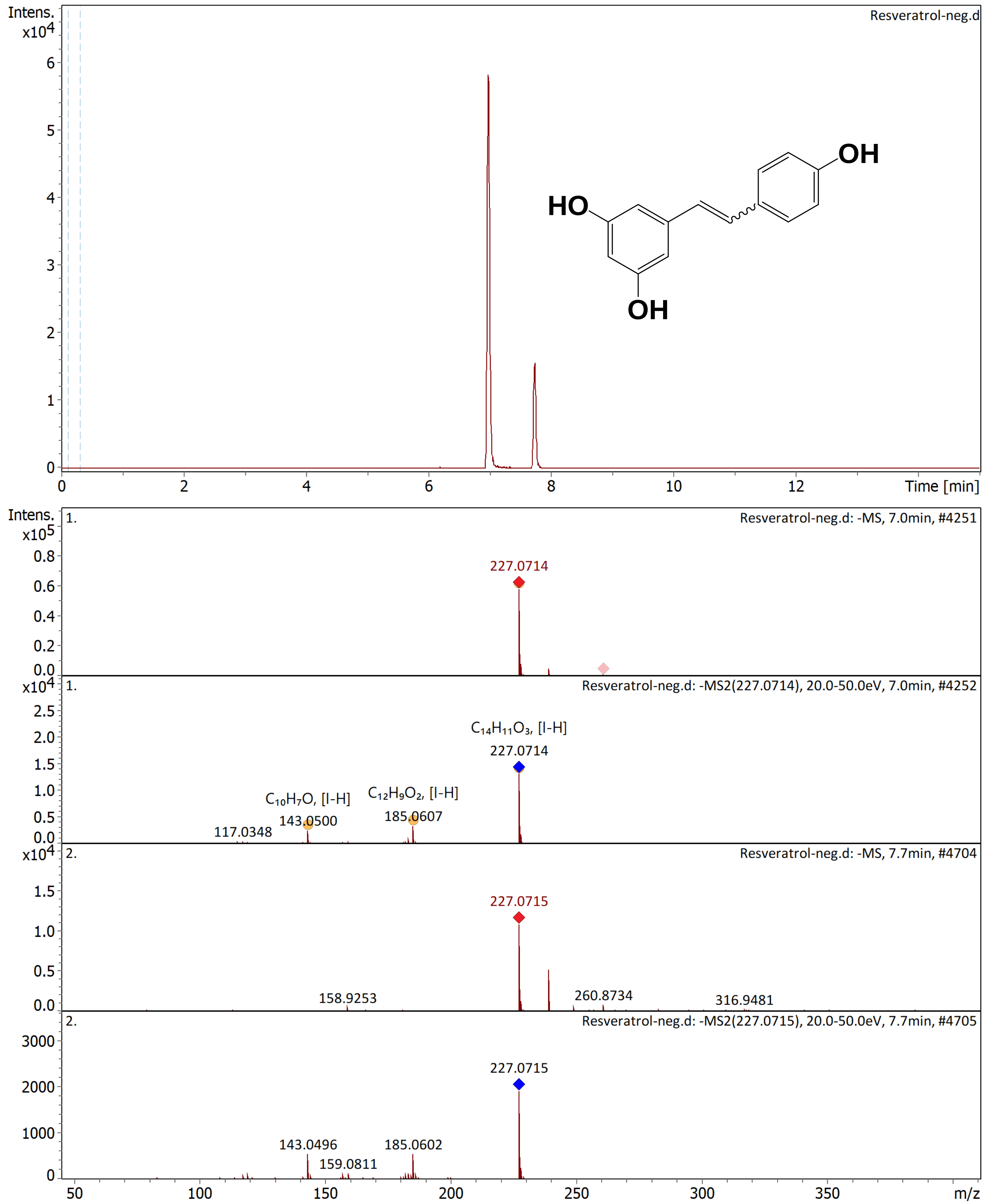

It has been shown in the Sun, 2026 (PNAS)

δ-viniferin

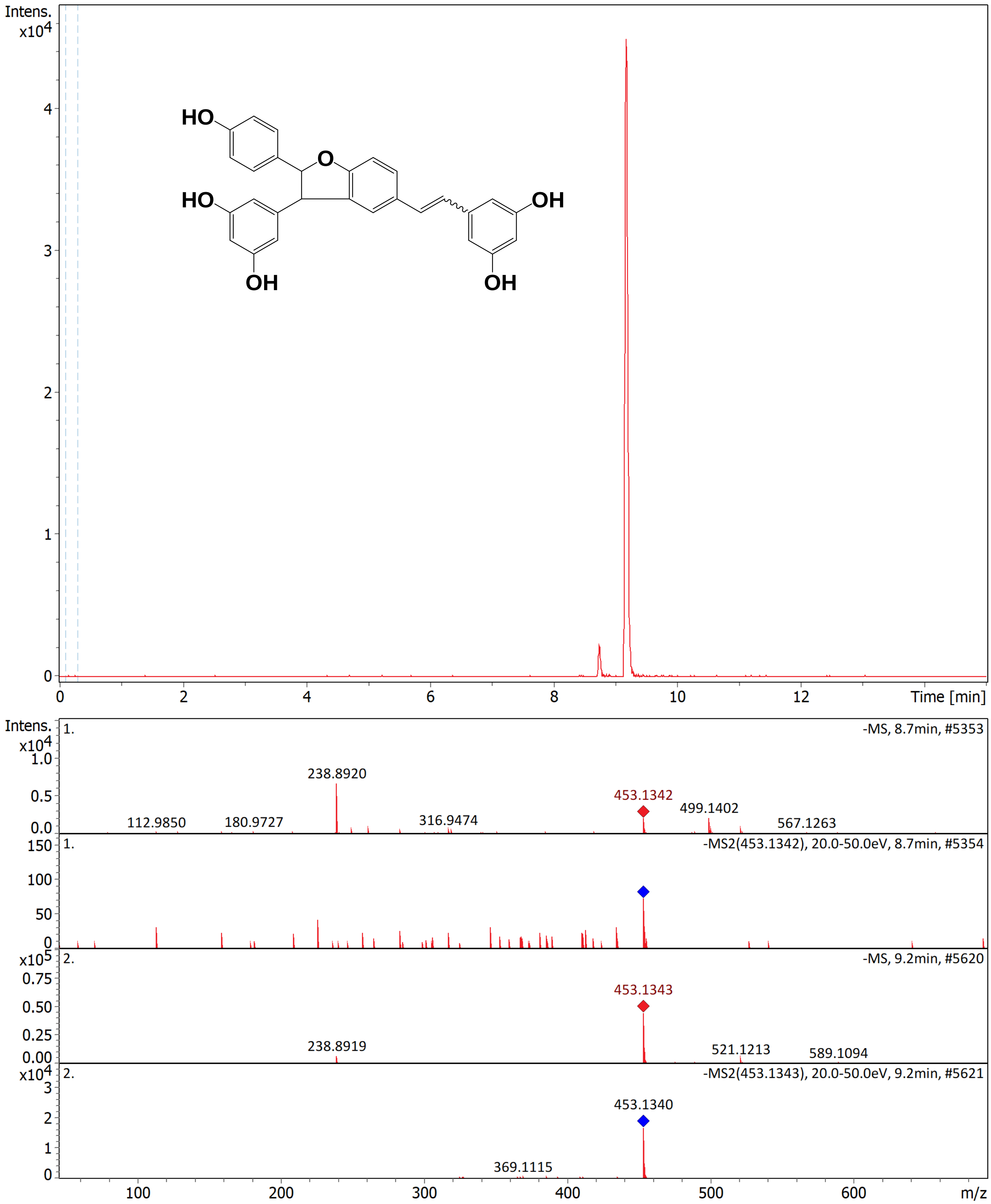
Intens.
x10^4
4
3
2
1
0
0
2
4
6
8
10
12
Time [min]
HO
O
HO
OH
OH
OH
Intens.
x10^4
1.0
0.5
0.0
1.
-MS, 8.7min, #5353
238.8920
453.1342
499.1402
112.9850
180.9727
316.9474
567.1263
150
100
50
0
1.
-MS2(453.1342), 20.0-50.0eV, 8.7min, #5354
x10^5
0.75
0.50
0.25
0.00
2.
-MS, 9.2min, #5620
453.1343
238.8919
521.1213
589.1094
x10^4
3
2
1
0
2.
-MS2(453.1343), 20.0-50.0eV, 9.2min, #5621
453.1340
369.1115
100
200
300
400
500
600
m/z

Taxifolin

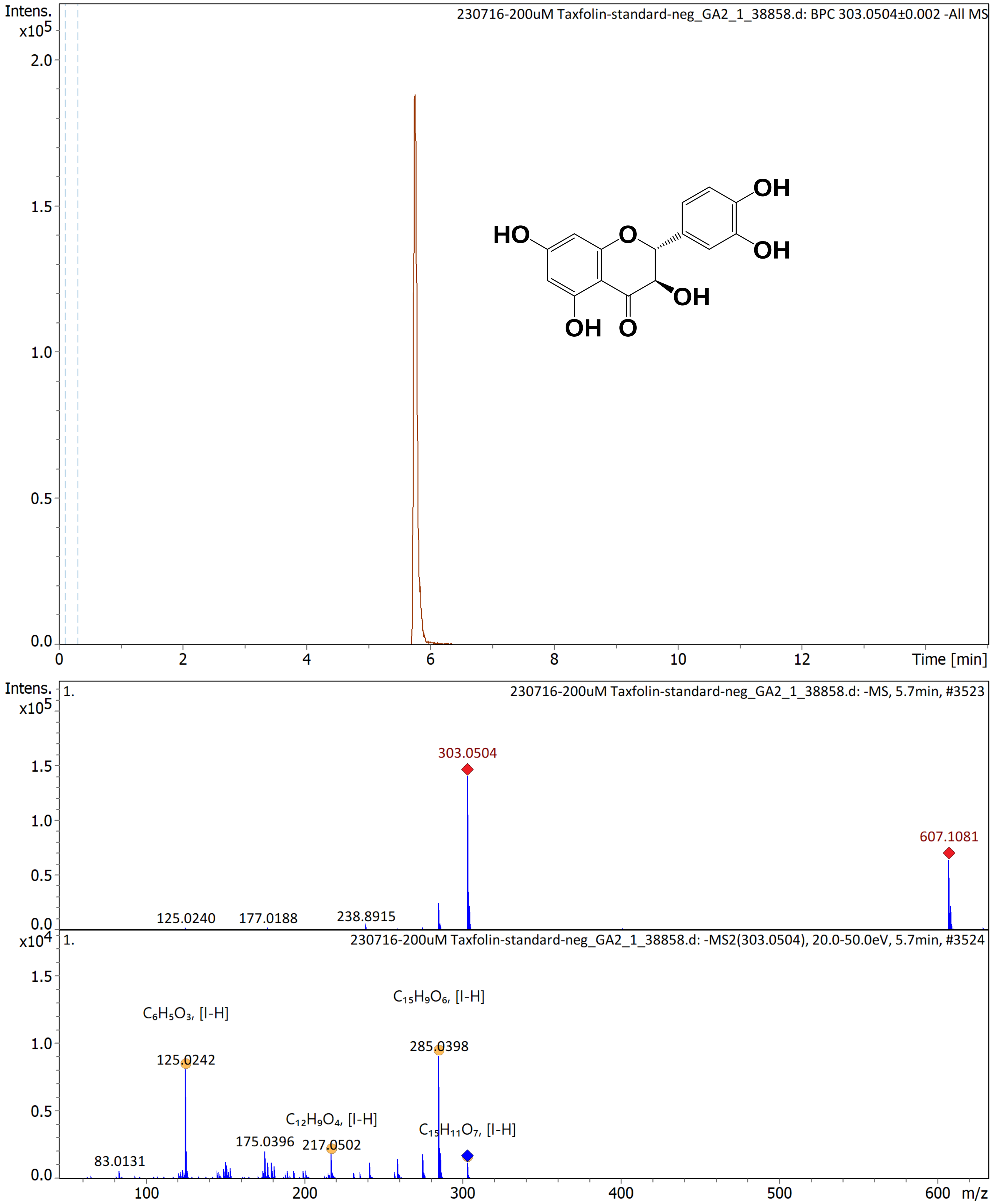

It has been shown in the Sun, 2026 (PNAS)

Taxifolin-3'-*O*-glucoside

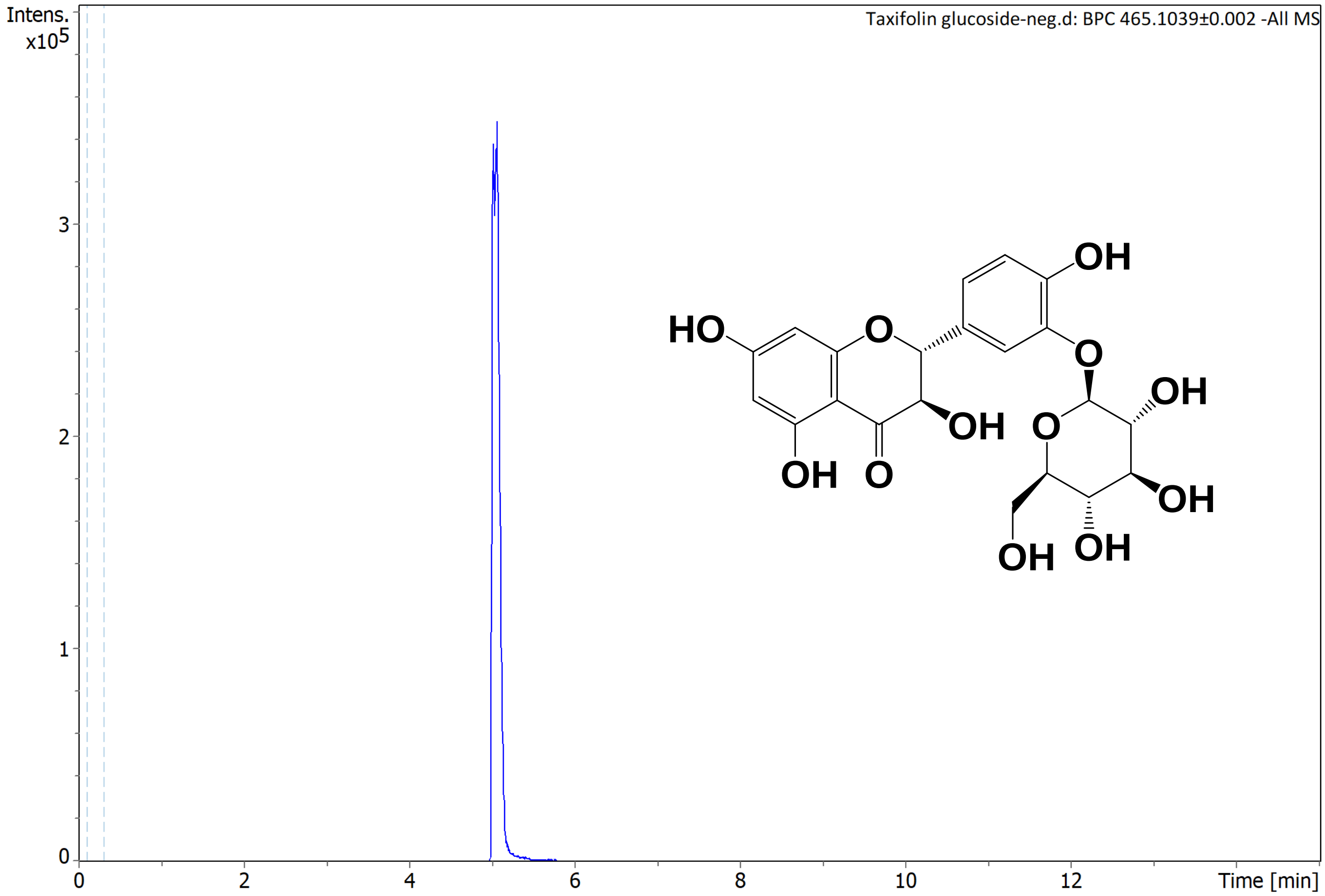

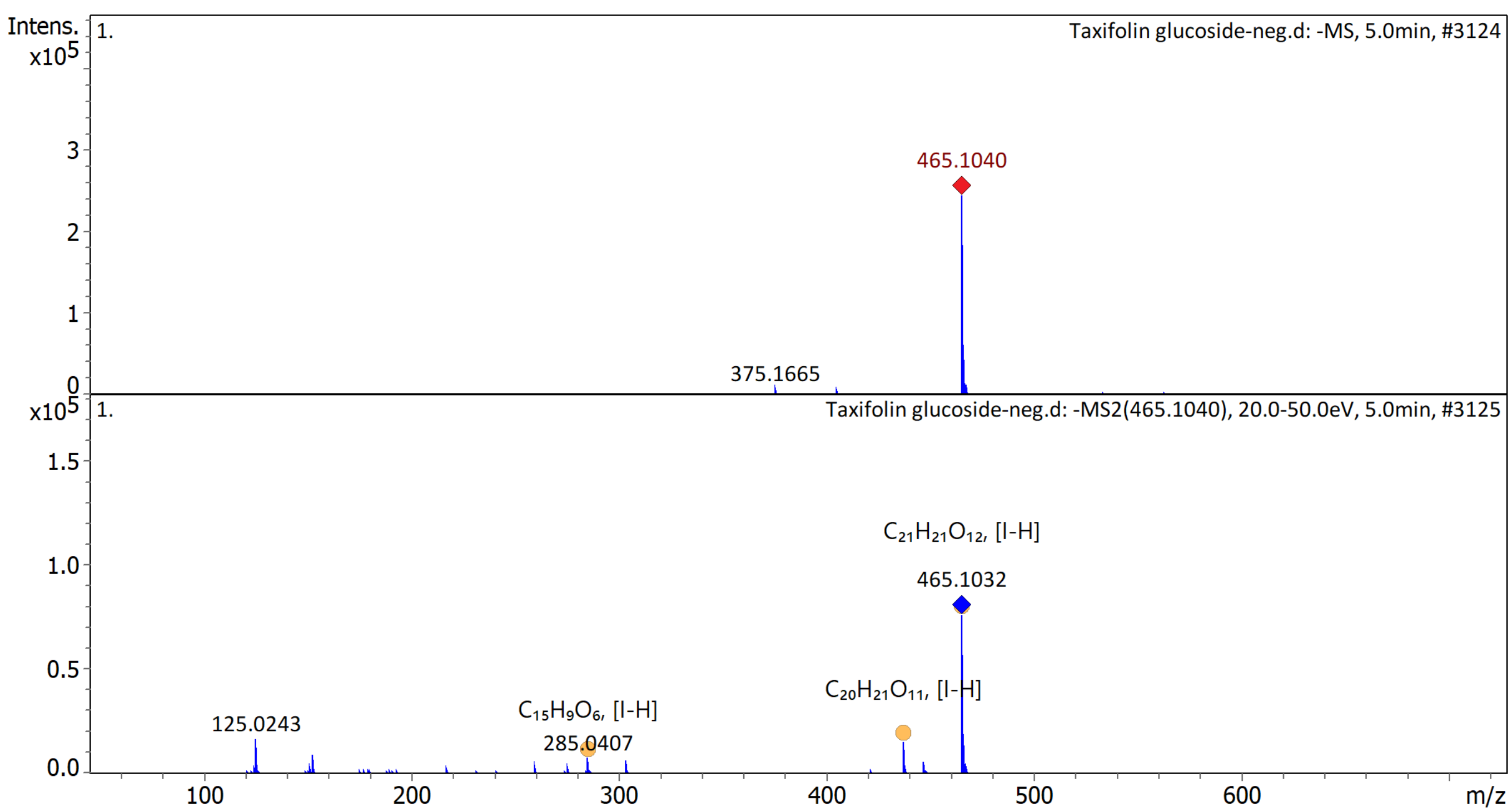

It has been shown in the Sun, 2026 (PNAS)

Quercetin

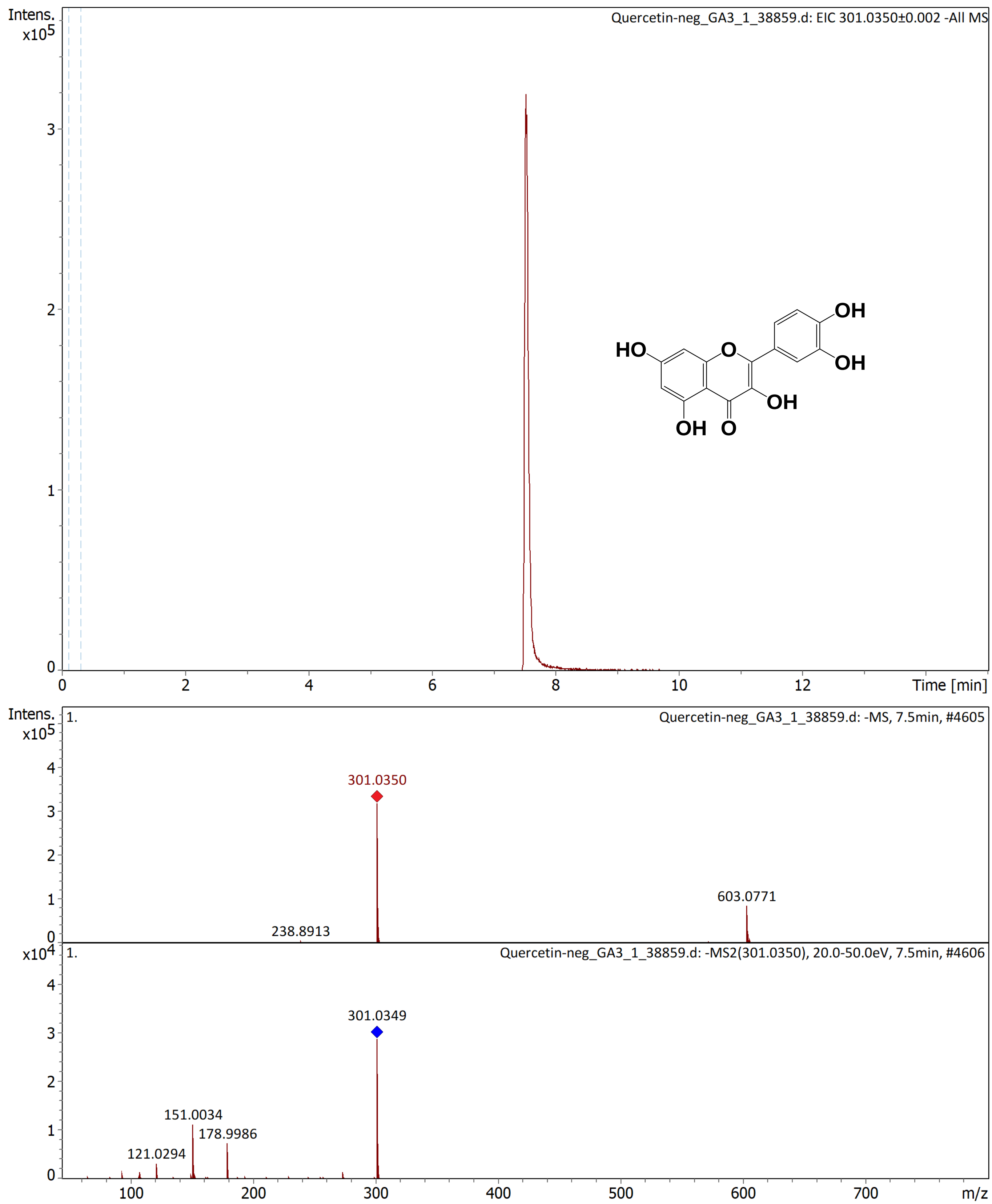

It has been shown in the Sun, 2026 (PNAS)

Quercetin-3'-O-glucoside

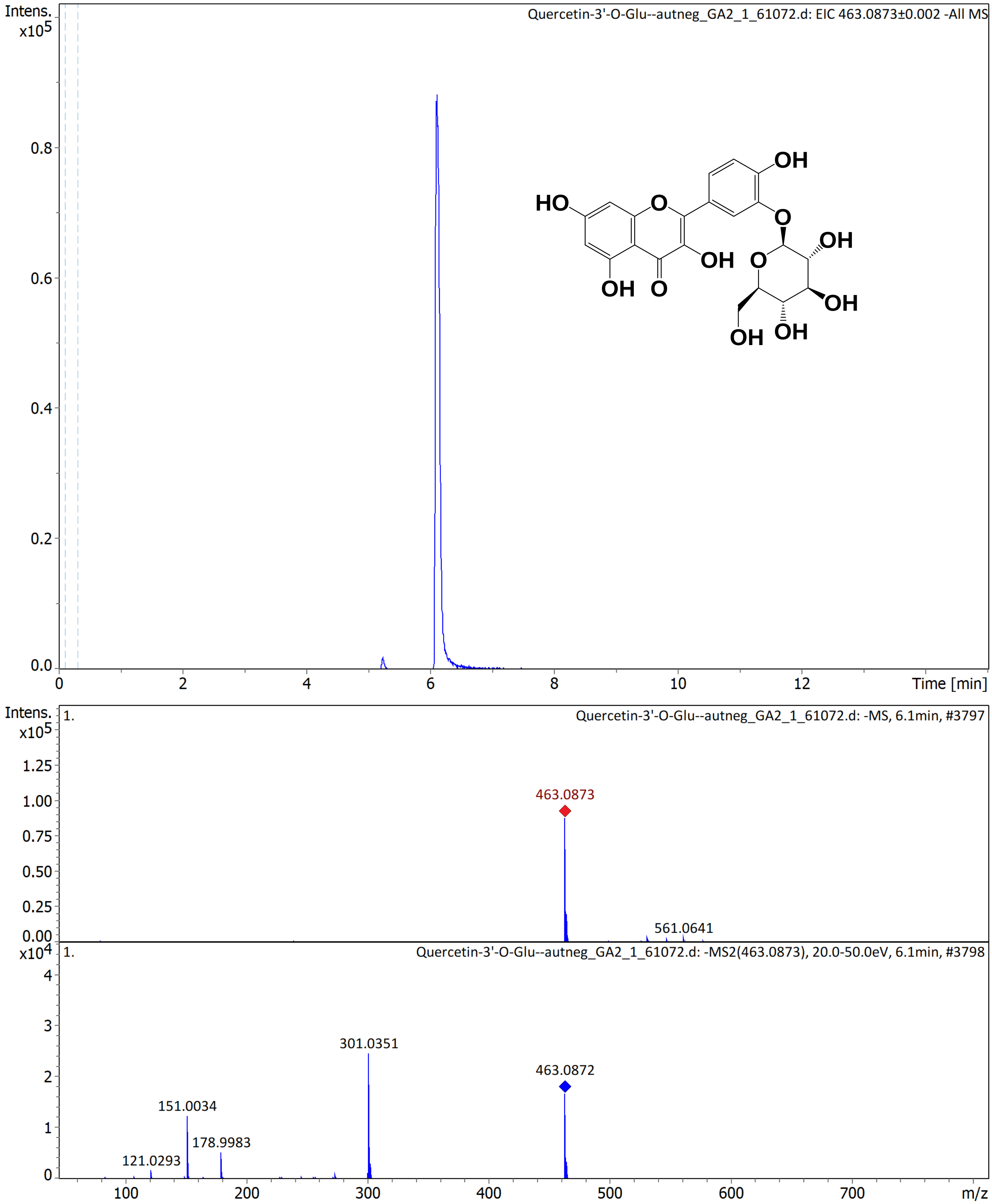

Supplement: Supplementary file 5 [file supplementary_file_2_wrag230.pdf]
